# Effectiveness of trained religious leaders’ engagement in maternal health education on improving maternal health service utilizations: Protocol of cluster randomized controlled trial in Hadiya Zone, Southern Ethiopia

**DOI:** 10.1371/journal.pone.0296173

**Published:** 2024-04-10

**Authors:** Abinet Arega Sadore, Yohannes Kebede, Zewdie Birhanu

**Affiliations:** 1 Department of Health, Behaviour and Society, Faculty of Public Health, Institute of Health, Jimma University, Jimma, Ethiopia; 2 School of Public Health, College of Medicine and Health Sciences, Wachemo University, Hossana, Ethiopia; Addis Ababa University School of Public Health, ETHIOPIA

## Abstract

**Background:**

Despite the many supply- and demand-side interventions aimed at increasing uptake of maternal health service utilizations, the maternal and new-born health service utilizations remains low. Religious leaders have the power to inhibit or facilitate effective adoption of maternal health service utilizations to promote maternal health. However, evidence on the roles of religious leaders in promoting maternal health in developing world is not fully known. Therefore this cluster-randomized trial is designed to evaluate the effects of trained religious leaders’ engagement in maternal health education in improving maternal health service utilization and knowledge of obstetric danger signs.

**Methods:**

A community based cluster randomized control trial in which the study kebeles are randomly assigned into intervention and control groups will be conducted. The sample size is calculated using stata software. Three hundred six pregnant mothers will be enrolled in each group. A baseline study will be conducted before the intervention and post-intervention evaluation will be conducted after four months of intervention. Religious leaders will be selected and trained to lead participatory sessions on maternal health. Data on maternal health service utilizations, knowledge about obstetric danger signs, attitude towards skilled delivery service utilization and perception of pregnancy risk will be collected from a repeated cross sectional household survey. Effect of intervention will be assessed using multivariable logistic regression with generalized estimating equation model. Data will be analyzed using STATA software. For qualitative study, coded transcripts will be further analyzed and summarized in narratives for each theme and sub-themes.

**Discussion:**

This is one of the first trials to evaluate the effectiveness of trained religious leaders’ engagement in maternal health education and will provide much needed evidence to policy makers about aspects of functionality and the religious leaders engagement required as they scale-up this programme in Ethiopia.

## Background

The number of women dying due to pregnancy and childbirth is way too high. In 2017, nearly 300,000 women passed away from complications during and after giving birth. The vast majority of these deaths (94%) occurred in low-resource regions and most of them could have been prevented. Sub-Saharan Africa and Southern Asia represented 86 percent (254,000) of the total number of maternal deaths, with Sub-Saharan Africa accounting for two-thirds (196,000) and Southern Asia for roughly one-fifth (58,000) [1, 2].

Socio-cultural influences are really important when it comes to maternal and child health behaviours in SSA, like going to antenatal check-ups [3–7]. The dynamics of power in the family and in the community can have an effect on whether or not people will choose to get health care, which is a finding of a research project done in northern Ghana. Basically, it seems like someone usually needs to give the okay for someone to get medical care, so that could be holding people back from getting the health care they need [8].

In developing countries, there are plenty of obstacles that prevent women and newborns from accessing maternal and neonatal health services. People aren’t aware of the services, their cultures and traditions make them hesitant, their families don’t always support them, they prefer to do things at home, transportation and costs can be an issue, and religious or traditional leaders may oppose them [9–11].

In Ethiopia, there are high rates of maternal deaths and poor health outcomes for women, which could be due to the fact that too many women aren’t using the available maternal health services like antenatal, skilled birth and postnatal care [12, 13]. Having access to health facilities is a big factor in how healthy and safe a mother is during her pregnancy and childbirth, especially for women living in places with limited resources [14].

Having a skilled professional present at childbirth and having care available in an emergency can help lower the risk of a mother dying during childbirth. Furthermore, simple and inexpensive measures like breastfeeding, keeping babies warm and dry, and providing treatment for any infections can help lower the number of new-born deaths. Also, it’s important to put effort into strengthening health systems and making treatments more accessible, especially through local clinics. Research has shown that a comprehensive plan that includes all these interventions is most effective [15].

Getting family members, traditional and religious leaders involved in helping out with maternal health behaviours is a great way to help spread awareness and generate interest. Religious organizations can be used as a way to increase knowledge about maternal and neonatal health services.

Faith-based organizations have the potential to help bridge the health gap, and religious institutions are highly trusted and respected, making them great for supporting public health initiatives [16]. Similarly, [17] religious figures, as well as medical professionals and healthcare workers, are key influencers when it comes to how people think, feel, and act, according to studies. Religious leaders have the power to influence people’s health habits on all levels, from individual to global, which can have a huge effect on the health of a community. This is done through providing health education and helping people make healthy choices [18]. Additionally, faith leaders have an impact on health habits, which is consistent with the goals set out in the Ottawa Charter for Health Promotion as they are seen as helping to increase community involvement [18]. Communities need to be given the power to take part in and manage their own matters. A religious leader is in an excellent spot to motivate people to change their behavior since they’re an important part of the community. Reports on programs that involve faith-based groups show that clergy members were successful in helping bring about changes in behaviour, especially with people who are hard to reach [17, 19]. Other research has highlighted the significance of faith leader’s effect on health habits [20]. Similarly, faith leader’s impact on behaviour has been associated with Bible-based teachings that support healthy living [17, 21]. Although some studies have proposed that spiritual leaders have a part to play in impacting congregant’s health behaviour, there is not much information out there on how much of an influence this is, and what the mechanisms behind it are [22].

Most people in Ethiopia are part of a religious group, so religion is a major part of the culture. Religious institutions have access to many people because of their places of worship. Plus, religious leaders are often leaders in the community and are the people who provide people with information. They can help spread the word about maternal and neonatal health services that aren’t being used as much as they should be. Therefore this cluster-randomized trial is designed to evaluate the effects of trained religious leaders’ engagement in maternal health education in improving maternal health service utilization and knowledge of major obstetric danger signs.

## Objectives

### General objective

The overall goal of this study is to develop understanding of contributions of religious leaders to maternal health promotion and evaluate effectiveness of trained religious leaders’ engagement in maternal health education versus usual care on maternal health service utilizations.

### Specific objectives

#### Objective 1

To explore perception of pregnant mothers and religious leaders on engagement of religious leaders in promoting maternal and child health in rural setting: qualitative exploratory study.

#### Objective 2

To assess effectiveness of trained religious leaders’ engagement in maternal health education in improving knowledge and recognitions of danger signs during pregnancy and child birth.

#### Objective 3

To assess effectiveness of trained religious leaders’ engagement in maternal health education on antenatal care service utilization

#### Objective 4

To assess effectiveness of trained religious leaders’ engagement in maternal health education on skilled delivery service utilization

## Hypothesis

We hypothesize that trained religious leaders’ engagement in maternal health education will increase the proportion of antenatal care, skilled delivery service utilizations and knowledge of major obstetric danger signs.

## Methods

### Study design

This study is a cluster randomized control trial to assess effectiveness of trained religious leaders’ engagement in maternal health education on maternal health behaviours. A total of four objectives will be answered from this study. The first objective is qualitative study, designed as explorative grounded theory. Objectives 2, 3 and 4 are designed as a community-based cluster randomized control trial, which evaluates the effectiveness of trained religious leaders’ engagement in maternal health education on maternal health behaviours (ANC, safe delivery service utilization and knowledge of danger signs during pregnancy and child birth).

### Source population

All pregnant women living in Amaka and Lemo districts of Hadiya Zone, Southern Ethiopia.

## Study population

Pregnant women in selected kebeles of districts in Hadiya Zone, Southern Ethiopia.

## Inclusion and exclusion criteria

### Inclusion criteria

Pregnant women less than 20 weeks gestational age

Living in selected kebele

Pregnant women willing to participate in the study

### Exclusion criteria

Pregnant women who are seriously ill and unable to communicate will be excluded from the study.

## Primary outcome variables

ANC Utilization

Skilled delivery service Utilization

### Secondary outcome variables

Knowledge of the major obstetric danger signs

Birth preparedness and complication readiness

Attitude towards skilled delivery service utilization

Perception of Pregnancy Risk

Self-efficacy

All variables will be measured at the individual-level (i.e., for each pregnant mother) within study clusters.

## Sample size

In accordance with the main objective of the larger study, the sample size estimate is designed to detect changes in the proportion of skilled delivery service utilization. The sample size is calculated using stata software based on the following assumptions. Tail (s): One; effect size (d): 0.15; ***α*** error probability = 0.05; power (1-***β*** error probability) = 0.8, and allocation ratio (N1/N2) = 1. This gives a sample size of 155. Then, adding design effect, DE = 1+(m-1)ICC, where m = Average cluster size, m = 50, ICC = 0.02 based on a published study ICC [23], DE = 2, and allowing for a 10% loss to follow up, the total sample size is 682 (N1 = 341 and N2 = 341). N1 and N2 are sample sizes in the control and intervention group, respectively. Based on these assumptions, the estimated sample size is: 306 pregnant mothers per group to detect a 15-percentage point anticipated difference in the proportion of skilled delivery service utilization (an increase from 26% to 41%). This estimate is based on the baseline skilled delivery service utilization in the Ethiopia Demographic and Health Survey (26% from EDHS 2016, SNNP region report) in the control group [24].

### Sample size for qualitative study

The sample size for the qualitative study will be determined based on the saturation of information. However, for a planned purpose, 4 focus group discussions and 12 in-depth interviews which are among religious leaders and pregnant mothers will be considered.

## Randomization and sampling procedure

First, 2 districts out of the 13 districts in Hadiya zone are selected purposely. Second, Non-adjacent 12 kebeles (clusters) are selected from two districts randomly. A pregnancy test will be done for the study women at the beginning of the surveillance. Then identified pregnant women with gestational age less than 20 weeks will be included in the study.

### For qualitative study

A purposive sampling technique will be used to select the study participants for the in-depth interviews. Participants (religious leaders) will be selected based on their acceptance by their followers from each religion domination.

## Randomization

There are a total of 12 clusters for this cluster randomized trial. Researchers and kebeles’ leaders, religious leaders and HEWs will perform randomization to generate comparable groups and eliminate the source of selection bias in assigning kebeles (clusters) to groups of intervention and control. Simple randomization with 1:1 assignment is applied to assign clusters to either the control group or the intervention group. First, 12 non-adjacent clusters are randomly selected. The 12 clusters are then listed alphabetically. The list of random numbers is generated in MS Excel 2010 and the generated values are pinned by copying them as ’values’ next to the alphabetical list of clusters. They are sorted in ascending order according to a random number generated. Finally, the first 6 clusters are selected as intervention clusters and the last 6 as control clusters. A statistician blinded by the research group and not participating in the study generates an assignment sequence and randomizes the clusters (figure below). The intervention group is assigned to receive training from religious leaders on maternal and neonatal health and the control group will be left to continue the existing practices.

### Controlling contaminations and spill-over effects

First, two districts are selected purposely from 13 districts in the Hadiya Zone. From two selected districts, 12 non-adjacent clusters are purposely and the clusters are allocated into either intervention or control group by simple randomization. The trained religious leaders will be assigned to provide intervention for pregnant mothers based on their residency of each cluster.

## Trial design

**Fig 1:**
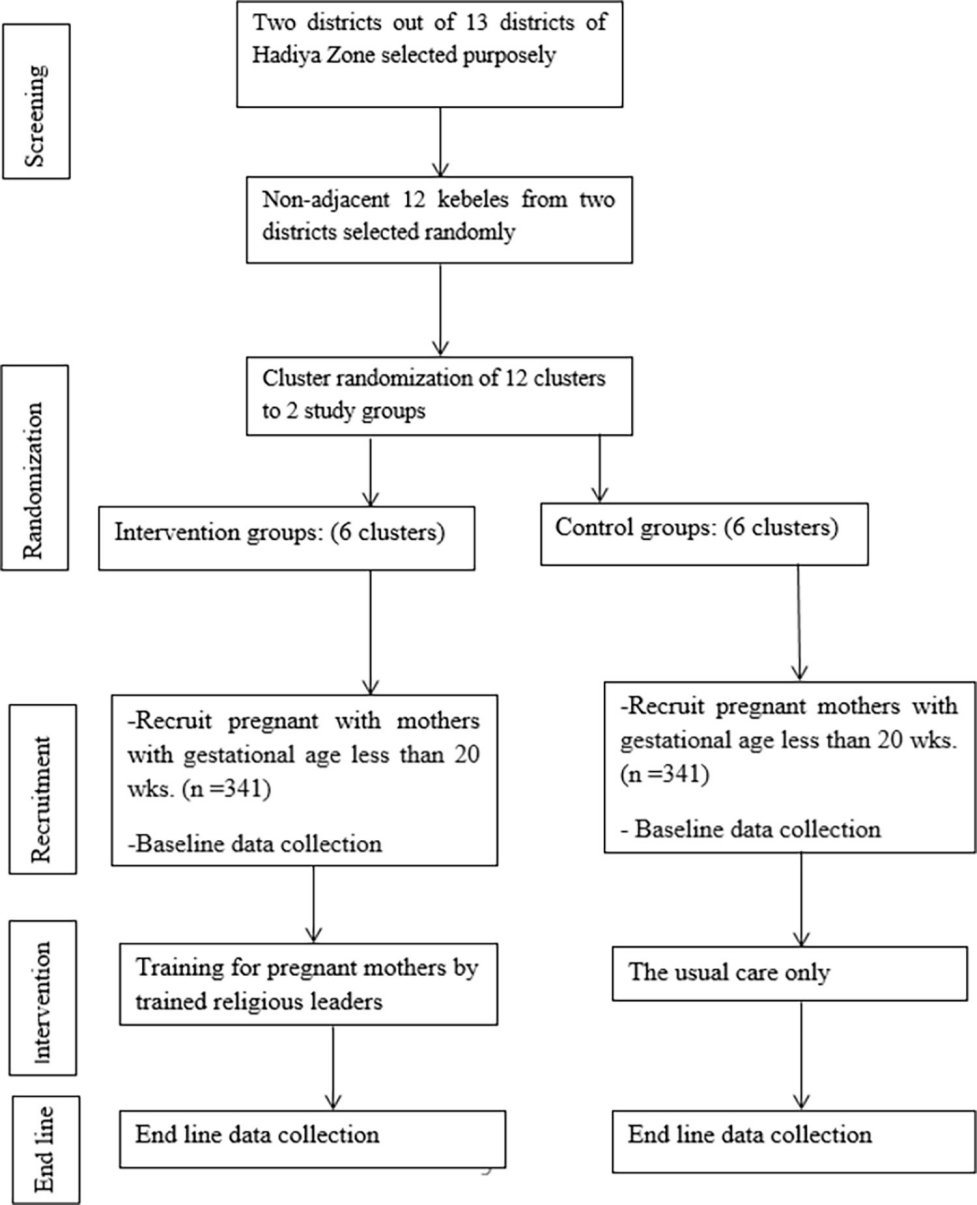
Diagrammatic representation of the randomization of study cluster (kebeles).

This study is a parallel two-arm a cluster randomized controlled trial with 8 clusters. The trial arms are as follows: 1) religious leader training and 2) standard cares. The kebeles health posts are designated as clusters for the trial. Outcome assessments will be made using repeat cross-sectional surveys at baseline (prior to intervention roll-out) and at 4 months post intervention (i.e. the end line). A schematic for the trial design is displayed in (Fig 1).

## Sampling techniques

Considering the number of kebeles that can meet the sample size, the study includes 12 kebeles. The kebeles (cluster at health post level) are randomly selected and assigned to the intervention and control groups). Distance among the kebeles is considered and some kebeles are left in between to serve as a corridor and avoid contamination from the religious leaders, and to minimize the likelihood of contact between religious leaders and thus the exchange of information among the groups.

## Intervention

For 4 months, trial participants in the intervention group will receive behavioural change communication on maternal health, while those in the control group will receive usual care (Table 1). In collaboration with health extension workers, kebeles leaders and religious leaders, the researchers and field workers will arrange an appropriate training place in the intervention clusters to train pregnant mothers on maternal health.

**Table 1:** Participants’ timeline of enrolment, intervention and assessment.

Activities	Month 1	Month 2	Month 3	Month 4	Month 5	Month 6
Participant enrolment						
Baseline assessment						
Intervention						
End line assessment						

### Training of religious leaders and pregnant mothers

Protestant, Orthodox, and Muslim are three religion domains in Hadiya Zone. First, the religious organizations in the study districts will be identified. Then the local religious leaders from each clustered kebele will be recruited based on religious educational status (educational status greater than or equal to diploma), acceptance by their followers and popularity, in collaboration with religious organization leaders, health extension workers, and kebele leaders. A total of 16 religious leaders will be recruited to be trainers of pregnant mothers. Then after the potential religious leaders are recruited, the two days training will be given for them. Recruited religious leaders are expected to give training on the topics (maternal health) for four sessions to promote healthy maternal behaviours for the members of their religion.

The trained religious leaders will deliver a total of four group training sessions for the pregnant mothers they are assigned with the same training procedures provided by the researcher during training of religious leaders. The first training session will be conducted at the beginning of the intervention whereas the second session of similar content will be repeated after one month of the first intervention session and the third and fourth training sessions will be after one months of the second intervention session. Religious leaders will be trained to conduct culturally appropriate training sessions with pregnant mothers using facilitator manual and posters prepared in the local language.

After training sessions, every participant will receive a copy of the visual materials (posters) containing the key messages for promoting maternal health behaviours. Direct, interactive and participatory learner and activity oriented instructional strategies will be delivered. Talks, group discussions, group work exercises, demonstrations, role plays, storytelling, simulation, case studies and problem-solving will be used to enhance knowledge, attitude, and behaviours on maternal and neonatal health, (Table 2 shows over protocol of intervention).

**Table 2:** Intervention protocol.

Session	Content of the message	Dose	Strategy of delivery	Frequency	Compliance parameters	Responsible person
Session 1	✓ Major causes of maternal and new-born Illness and death	1:00 hour	• Brain storming • Group education/ Group work • Case study/ case scenario • Question and Answer (Q&A) • Agree/ Disagree exercises • Group Presentation	Once at their 2^nd^ trimester period	• % pregnant mothers participated	Researcher and trained religious leaders
Session 2	✓ Focused Antenatal Care ✓ About danger signs during pregnancy	1:00 hours	Brain storming • Group education/ Group work • Case study/ case scenario • Question and Answer (Q&A) • Agree/ Disagree exercises • Group Presentation	Once at their 2^nd^ trimester period	• % pregnant mothers participated	Researcher and trained religious leaders
Session 3	✓ Birth preparedness and complication readiness ✓ Danger signs, labor and childbirth, ✓ Components of postnatal care and Postnatal danger signs and neonatal danger signs	1:00 hour	Brain storming • Group education / Group work • Case study/ case scenario • Question and Answer (Q&A) • Agree/ Disagree exercises • Group Presentation	Once at their 3^rd^ trimester period	• % pregnant mothers participated	Researcher and trained religious leaders
Session 4		1:00 hour	Brain storming • Group education / Group work • Case study/ case scenario • Question and Answer (Q&A) • Agree/ Disagree exercises • Group Presentation	Once at their 3^rd^ trimester period	• % pregnant mothers participated	Researcher and trained religious leaders
	**Providing specific take-home print materials** ✓ ANC practices ✓ SDS practice	• Poster • Information card • Flip chart	-	Delivered during in each training session	• % of pregnant mothers received print materials	Researcher and trained religious leaders
	**End line data collection**	-	-	at 4^th^ month of intervention	% pregnant mother interviewed	Researcher, supervisors data collectors.

### Control group (standard care)

The control group in this study will be on the existing routine maternal service without a provision of religious leaders’ training intervention. In this arm, there will be no intervention by the researchers, rather baseline and end line data will be collected.

### The focuses of training (intervention key messages) on

**Antenatal care:** (taking TT injection, taking Calcium Consumed 100+ IFA tablets, eating more food during pregnancy, eating Green vegetables, eating Fruits during pregnancy, drinking milk during pregnancy, eating pulses and beans during pregnancy, taking nap/rest, regularly during pregnancy, Husband accompanying for ANC (regularly /mostly), Human immunodeficiency virus (**HIV) and syphilis** test, tobacco use and substance use. **Benefits of early and exclusive breastfeeding (**Provides the best nutrition for the new-born Is easily digested and efficiently used by the baby’s body, Protects against infection and other illnesses, Offers some protection against allergies, Is cost-effective and affordable, Promotes mother-baby bonding. **Unhealthy beliefs and practices about feeding new-borns**, **Hygiene during pregnancy**, **Breastfeeding and contraception, Benefits of birth spacing: (**Maternal mortality, fetal death (miscarriage or stillbirth), neonatal mortality, Anaemia in the mother during subsequent pregnancies, Postpartum inflammation of the endometrium lining the uterus, Premature rupture of the amniotic membranes surrounding the fetus, Premature birth, Intrauterine growth retardation and a low birth-weight baby and Malnutrition of new-borns and infants due to insufficient breast-milk**).**

**Prenatal Danger signs: (**Severe headache, Blurred vision, Fatal movement absent, High blood pressure, Edema of the face/swelling, Oedema of the hands/leg swelling, Convulsions, Excessive vaginal bleeding, Severe lower abdominal pain and Leaking fluid (meconium stained).

**Labor/delivery Danger signs: (**Excessive vaginal bleeding, Foul-smelling discharge, High fever, Baby’s hand or feet coming out first, Baby is in abnormal position, Prolong labor (.12 hours), Retained placenta, Rupture uterus, Cord prolapse, Cord around neck and Convulsion).

**Postpartum Danger signs:** (Excessive vaginal bleeding, Foul-smelling discharge, High fever, Inverted nipples, Tetanus, Retained placenta, severe abdominal pain, Convulsions **and** Engorged breasts/swelling of breasts).

**Neonatal Danger signs: (**Poor feeding or unable to suck, Diarrheal, Redness around the cord, Red eye/discharging eyes, Difficult breathing, Yellow coloration of the skin/jaundice, Hypothermia/shivering, Blisters on skin/Skin lesion, Baby doesn’t cry, Fever, Unconscious, Fast breathing, Chest in drawing, Doesn’t pass urine, Doesn’t pass stool and Convulsions.

**Consulting doctor or others health professionals:** if they have danger signs (during pregnancy, labor and childbirth, 42 days after delivery, and neonatal danger signs).

**Birth preparedness and complication readiness:** (plan for where to give birth, plan for a skilled birth attendant, plan to save money, plan for transportation and identification of compatible blood donors in case of emergency).

**Delivery at a health facility with a skilled provider** and **postnatal care:** (Women discuss FP method with husband Women adopted FP method after delivery Mother fed colostrum to the baby).

## Cluster and individual selection

The kebeles’(small administrative unit in Ethiopia) health posts are eligible for trial. All 40 health post units in the selected districts are eligible and 12 health posts are randomly selected for the trial using a computer random number generator. Pregnant mothers less than or equal to 20 gestational age are eligible to participate in the trial if they are living in the villages within the selected health post village areas.

### Identifying eligible pregnant mothers for the study

To identify the eligible pregnant mothers, pregnancy surveillance will be conducted among currently married women of reproductive age (15–49 years) permanently living in the selected districts. A pregnancy test will be done for the study women at the beginning of the surveillance. Identifying pregnant women will be a two-step process. First, all women will be screened using pregnancy screening questions adapted from the Kenya family planning enrolment questionnaire as published in the Lancet [25] (Table 3). If the answer to at least one of the six pregnancy screening questions is ‘yes’, pregnancy will be ruled out. Women who respond in a way that suggested the possibility of being pregnant is asked to provide a urine sample for a pregnancy test, which will be done using a dipstick in the respondent’s home. Once pregnancy is detected, the women will be recruited for the trial study.

**Table 3:** Pregnancy screening questions adapted from Kenya family planning screening questions.

S/No	Items	Yes or No
1	Have you given birth in the past 4 weeks?	
2	Are you less than 6 months post-partum or fully breastfeeding and free from menstrual bleeding since you had your child?	
3	Did your last menstrual period start with in the past 7 days?	
4	4 Have you had a miscarriage or an abortion in the past 7 days?	
5	Have you abstained from sexual intercourse since your last menses?	
6	Have you been using a reliable contraceptive (pills, injectable, and Norplant) method consistently and correctly?	

## Intervention assignment and masking

Randomization is stratified by kebeles (Kebele health post), with 12 groups in districts assigned control or intervention at a public randomization meeting. From each cluster (kebele), local actors (agricultural extension workers, kebele leaders, and heads of religious organizations) are invited. A number is assigned to each group, these numbers are written on small plastic balls and the balls are placed in a dark bag.

As soon as all of the balls are selected, each participant is instructed to draw a ball from the bag and recite the group number. On a piece of paper, cluster numbers are written in the order of selection. The participants should then put 12 pieces of paper with the numbers 1 through 12 in the dark bag. Next, they instruct everyone to select a piece of paper and read the number on it. Each piece of paper is corresponded to a different allocation sequence created by an independent statistician. The public allocation of each cluster to one of two groups is then accomplished using the corresponding sequence. The intervention team could not be masked to allocation due to the nature of the intervention that is being assessed. Both at the cluster and individual levels, the data collection team will be unaware of information about allocation. To eliminate bias, blinding is applied as follows. When the kebeles are randomly assigned to study groups, the researchers do not explain the study interventions and hypotheses to the participants.

## Compliance parameter

Eligible pregnant mothers in the intervention clusters will be repeatedly visited in a case of absence to solve the problem of compliance to a full intervention package. Despite these efforts, due to different reasons, the eligible women in the intervention group may not fully comply with the planned intervention as per recommendations within the intervention package. This might exert negative effect on the uptake of planned services by religious leaders. The impact of variability in the compliance to the proposed intervention requirements will be considered during analysis as a dos response function. Therefore, compliance checklist will be used to track the level of women’s compliance to our proposed intervention package to account during data analysis.

## Intervention fidelity

Fidelity of the intervention will be maintained based on the National Institutes of Health Behavioural Change Consortium developed best practice recommendations [26]. The intervention design has conceptual framework. Non adjacent clusters are selected to prevent information contamination. Equal numbers of clusters will be taken for the intervention and control groups from each district to balance variations. The intervention process will be pretested before the implementation of the trial. Each pregnant mother will receive equal numbers and frequencies of training, and the lengths of contacts within an intervention group will be similar to make the process standardized. Group education training will be given in a group using a training manual, role-playing, and mock counselling practice. The competency of recruited religious leaders will be monitored by pre-post training test. The test focuses on knowledge and attitudes towards intervention key messages. If the post-training test score of the trained religious leaders is below acceptable range, retraining will be given on identified gaps of intervention messages. Training sessions will be randomly selected for process evaluation and all selected sessions will be evaluated by one process evaluator. The process observer rated the educator using a ‘yes/no’ rating system on items such as using a training guide, provision of the whole content, duration and frequency of training, preparedness, accuracy, and ability to properly respond to questions. Intervention receipt will be assessed using checklists on knowledge of the pregnant mothers on maternal health behaviour through interviewing about their understanding of the core contents of the intervention. Intervention enactment will be assessed on utilizations of antenatal care and skilled delivery.

### Measurements and indicators

Trained interviewers will conduct face-to-face interviews using structured questionnaires. Questionnaires contain sections on socio-demographics, Reproductive history of respondent, maternal health service utilization, birth preparedness and complication readiness, knowledge of danger signs during pregnancy, labor and child birth, new-born and postpartum period, perception about pregnancy, childbirth, and the period immediately after childbirth, attitude towards safe delivery utilization and religious leaders’ engagement scale measurement tool. The uptake of antenatal care utilization will be assessed based on “at least one ANC attendance” (Women who have attended at least one ANC check-up during their current pregnancy) and “four or more ANC utilization” (Women who attended four or more ANC visits) during their current pregnancy as reported by the participant [27]. Safe delivery service utilization will be assessed based on women who gave birth in health center and hospital by assistance of health professionals that have midwifery skills including Midwife nurse, Nurse, Health Officers and Doctors as reported by the participant [28].

Knowledge of danger signs during pregnancy includes 14 items, knowledge of labor and childbirth includes 9 items, and knowledge of neonatal danger signs includes 11 items. All knowledge score will be assessed adding total number of correct spontaneous responses to maximum number of items with a minimum score of 0 and maximum number of items (0 when a mother mentioned none of the key danger signs and maximum number when the mother mentioned all the danger signs). Spontaneous response is respondents’ naming of danger signs without giving option of the respective signs. Accordingly, two categories will be developed for knowledge of danger signs (Good and poor categories). Women who mention at least three neonatal danger signs will be considered to have good knowledge whereas those who mentioned less than three of the danger signs will be labelled to have poor knowledge as stated by several studies [29]. The BPCR questionnaire is adapted and modified from BPCR monitoring tools for maternal and new-born health [30]. The attitude toward the use of safe delivery services consists of 12 items that will be measured using a Likert scale in which respondents will be asked to strongly agree, agree, neutral, disagree, and strongly disagree. The overall attitude score will be calculated by adding the items after reverse scoring for negatively phrased sentences. A higher composite score indicated a more favourable attitude.

### Perception of Pregnancy Risk Questionnaire (PPRQ)

This is a self-report questionnaire consisting of nine visual analogue scales for measuring the perceptions of pregnancy risk among pregnant women. The questionnaire consists of 2 subscales containing 4 questions about risk to yourself (the mother) (e.g. ’Are you at risk for caesarean section?’). Her five questions about risks to babies (e.g., "Is my baby at risk of having birth defects?"). “Respondents will be asked to put a vertical mark through the line to indicate their assessment of risk for each Item,” (yielding a score ranging from 0–100). The overall PPRQ score will be obtained by adding the scores for each of the 9 points and dividing by 9 to get a score of 100. A higher score indicates a higher perceived risk [31].

### Data collection and management

The principal investigator will train the data collectors and supervisors on two consecutive days on instructions like: quantitative methods, research guidelines, role-plays (demonstrations), declarations of consent, responding to participants, data collection using mobile health, ethical procedures and general information, and research objectives.

Data will be collected using a mobile application called the Open Data Kit. (ODK). In the first step, a pre-intervention survey will be conducted after randomization and allocation. There will be two data collections, including pre-intervention and post-intervention data. Data will be collected at 4-month intervals to have good longitudinal data. The data type is repeated cross-sectional data. Experienced data collectors who have a health background and speak local language will be employed to collect the data. In addition, a master’s degree with health profession will be hired to oversee the entire data collection process.

### Baseline data collection

Each data collector and supervisor will be assigned to a different cluster within the district. The interview will be expected to last approximately one hour and will be conducted in a quiet private room in the woman’s home. If the selected woman is not at home, the interviewer will attempt to visit and interview the household on up to 3 different days/times before replacing the woman with another woman. Data will be collected for a total of 15 days.

### End line data collection

The same procedure with the baseline data will be repeated to collect the end line data after one month of delivery. Data collectors with health background will be used to collect the data. Supervisors will be assigned to check for the daily activity, consistency, and completeness of the questionnaire and to give appropriate support during the data collection process. The data collectors and the supervisors will be assigned to a different cluster of a given district.

### Qualitative data collection

Focus group discussions and in-depth interviews will be conducted with religious leaders and pregnant mothers. Focus group discussions and in-depth interviews will be conducted by trained research assistants and principal investigators. Data will be collected by recording interviews by audiotape. Brief field notes will be made during the interview. Field notes will carefully be taken during data collection and analysis. These notes will collect observations and assumptions about what will be heard or observed and personal narratives about what will be felt by the researcher during an interview.

### Data quality control

To ensure data quality, data collectors and supervisors will be trained, and regular monitoring and follow-up will be done by supervisors and principal investigators. In addition, data integrity and consistency will be checked regularly on a daily basis. The questionnaire is translated into Amharic and then into English by a translator unfamiliar with the original questionnaire. Reliability and validity tests will be performed on the scale variables to standardize the questionnaire. Pilot test and pre-testing of the tool will be conducted in areas with similar characteristics to study population to ensure clarity, wording, logical order, and abbreviated patterns of questions. Pre-tested samples will not be included in the study and modifications are made.

### For the qualitative study

Quiet places, comfort, and auspicious times will be selected and organized to conduct a qualitative study with religious leaders to encourage maximum concentration. Study participants will also be asked to give factual answers by explaining the purpose and importance of the study and by ensuring the confidentiality of the data they will provide. To improve reliability between encoders, each coder will independently apply the codebook to a selected, rich transcript, and consider any differences in their encoding, which will be discussed and resolved.

## Operational definition

### Skilled delivery service utilization

Skilled delivery **service utilization will be measured based on** women who gave birth in health center and hospital by assistance of health professionals that have midwifery skills including Midwife nurse, Nurse, Health Officers and Doctors *as* reported by the participant.

### Antenatal care service utilization

Antenatal care service utilization will be measured based on “at least one ANC attendance” (Women who have attended at least one ANC check-up during their current pregnancy) and “four or more ANC attendance” (Women who attended four or more ANC visits) during their current pregnancy as reported by the participant.

### Birth preparedness and complication readiness

Birth preparedness: A woman will be classified as “well birth prepared” in the most recent pregnancy if she has accomplished three of the following practices: identified skilled health professional, saved money, identified transport or had delivery kit/materials. A woman who makes arrangements for birth in less than three of the four ways will be classified as “not well birth prepared”.

### Knowledge of obstetric danger signs

Women who mention at least three neonatal danger signs will be considered to have good knowledge whereas those who mentioned less than three of the danger signs will be labelled to have poor knowledge as stated by several studies.

### Perception of pregnancy risk

Risk perception is operationalized using the adapted Perception of Pregnancy Risk Questionnaire (PPRQ) developed by Heaman & Gupton. Higher scores will indicate higher levels of perceived risk.

### Attitude towards skilled delivery service utilization

Attitude will be measured by using five point Likert scale. Positive attitude will be scored by participants who respond above the mean of the attitude assessment questions and if below the mean they were categorized as having negative attitude.

## Statistical analysis

Data of baseline and end-line surveys will be combined and analyzed using Stata 14.1. Analyses will be based on the intention-to-treat principle and compared differences of the outcomes between the intervention and control at both individual and cluster levels. First, Univariate analyses will be performed to explore the characteristics of respondents. We then will use logistic regression with random effects to estimate the effect of the intervention on maternal health behaviour, adjusting for clustering. Generalized estimated equations (GEE) regression analyses adjusted for clustering will be used to test the effect of the intervention on promoting maternal health behaviour. We will repeat analyses for the primary and secondary outcomes adjusted for baseline differences by fitting an interaction term between study period (baseline vs intervention) and allocation in each model. Mixed-effects multilevel logistic regression model will be used to identify determinants of skilled delivery service utilizations.

### For qualitative study

Interviews and focus group discussions will be recorded and transcribed verbatim. Transcripts will be analyzed by using the Atlas.ti 7.0 qualitative software. Two analysts will independently code the transcript and then review, discuss, and refine the coding schemes until consensus is reached. Emerging concepts will be evaluated using the constant comparative method from grounded theory. This means that once the concept is defined, the previously analyzed interviews will be examined to check whether their content corresponds to the concept. Then a final version of the code-book will be developed to define the themes and sub-themes. Coded transcripts will be further analyzed and summarized into narratives for each theme and sub-theme. The results of the study will be presented, discussed, and validated during stakeholder meetings held at regional and county health offices.

## Discussion

In the experimental group, a behavioural change communication approach led by a trained religious leader will be given to pregnant mothers to improve maternal health service utilization and knowledge of knowledge of major obstetric danger signs. To ensure accessibility and sustainability, interventions are based on central and peripheral routes of persuasive messages. An individual’s ability to understand information depends on the quality of the information. This can be explained by two factors: Completeness and Accuracy of Information. This study adopted both two factors as the central route of the messages. Information completeness is when there is sufficient depth and breadth of information in the communication. Accuracy of information for this study is defined as the degree to which the information is accurate, precise, and unambiguous [32]. In the context of maternal health education by religious leaders, the accuracy of information requires that all information discussed to be accurate and consistent. The researchers argue that suggestion works when individuals are "unmotivated by topics or unable to process relevant arguments," which is found abundantly in the central route [33]. Therefore, individuals look for simpler cues, such as source reliability, aesthetics, and popularity. In this study, peripheral ways of persuading maternal health education messages will be considered when recruiting religious leaders.

If this intervention proves to be effective in improving the maternal health service utilization and knowledge of major obstetric danger signs, it will be scaled up in other parts of Southern region and other regions of the country.

### Trial status

From the 13 districts found in Hadiya Zone, Lemo and Amaka are elected purposely. Trainers’ and participants’ manuals were prepared in English and translated into Amharic, the language spoken locally. Information, education communication materials such as posters, leaflets, and visual teaching materials were developed in Amharic. This trial will be carried out from March 1, 2023 to July 2, 2023.

## Supporting information

S1 Checklist SPIRIT 2013 checklist: Recommended items to address in a clinical trial protocol and related documents*. (DOC)

S1 Protocol (DOCX)

## Abbreviations

ANC: Antenatal care
BPCR: Birth preparedness and complication readiness
CSA: central statistical agency
EDHS: Ethiopian demographic health survey
EmOC: Emergency obstetric care
FMOH: Federal Ministry of Health
HEWs: Health Extension workers
MCH: maternal and child health
MDG: Millennium Development Goal
PHCU: Primary Health Care Unit
PPRQ: Perception of Pregnancy Risk Questionnaire
SBCC: Social behavioural change communication
SDG: Sustainable development goal
WHO: World Health Organization

## References

[1] WHO, Trends in maternal mortality: 2000 to 2017: estimates by WHO, UNICEF, UNFPA, World Bank Group and the United Nations Population Division. Geneva:. 2019.

[2] Adedini S.A., et al., Role of religious leaders in promoting contraceptive use in Nigeria: evidence from the Nigerian urban reproductive health initiative. Global Health: Science and Practice, 2018. 6(3): p. 500–514. doi: 10.9745/GHSP-D-18-00135 30287529 PMC6172128

[3] Ahmed S., et al., Economic status, education and empowerment: implications for maternal health service utilization in developing countries. PloS one, 2010. 5(6): p. e11190. doi: 10.1371/journal.pone.0011190 20585646 PMC2890410

[4] Grede N., de Pee S., and Bloem M., Economic and social factors are some of the most common barriers preventing women from accessing maternal and newborn child health (MNCH) and prevention of mother-to-child transmission (PMTCT) services: a literature review. AIDS and Behavior, 2014. 18(5): p. 516–530. doi: 10.1007/s10461-014-0756-5 24691921

[5] Pell C., et al., Factors affecting antenatal care attendance: results from qualitative studies in Ghana, Kenya and Malawi. PloS one, 2013. 8(1): p. e53747. doi: 10.1371/journal.pone.0053747 23335973 PMC3546008

[6] Simkhada B., et al., Factors affecting the utilization of antenatal care in developing countries: systematic review of the literature. Journal of advanced nursing, 2008. 61(3): p. 244–260. doi: 10.1111/j.1365-2648.2007.04532.x 18197860

[7] Underwood C., et al., Role of community-level factors across the treatment cascade: a critical review. JAIDS Journal of Acquired Immune Deficiency Syndromes, 2014. 66: p. S311–S318. doi: 10.1097/QAI.0000000000000234 25007202

[8] Moyer C.A., et al., “It’s up to the woman’s people”: how social factors influence facility-based delivery in Rural Northern Ghana. Maternal and child health journal, 2014. 18(1): p. 109–119. doi: 10.1007/s10995-013-1240-y 23423857

[9] Miller N.P., et al., Barriers to the utilization of community-based child and newborn health services in Ethiopia: a scoping review. Health Policy and Planning, 2021. 10.1093/heapol/czab047 PMC8496769 33885143

[10] Kifle D., et al., Maternal health care service seeking behaviors and associated factors among women in rural Haramaya District, Eastern Ethiopia: a triangulated community-based cross-sectional study. Reproductive Health, 2017. 14(1): p. 6. doi: 10.1186/s12978-016-0270-5 28086926 PMC5237279

[11] Dahab R. and Sakellariou D., Barriers to Accessing Maternal Care in Low Income Countries in Africa: A Systematic Review. International journal of environmental research and public health, 2020. 17(12): p. 4292. doi: 10.3390/ijerph17124292 32560132 PMC7344902

[12] Mekonnen Y. and Mekonnen A., Factors influencing the use of maternal healthcare services in Ethiopia. Journal of health, population and nutrition, 2003: p. 374–382. 15038593

[13] Berhan Y. and Berhan A., Review of maternal mortality in Ethiopia: a story of the past 30 years. Ethiopian journal of health sciences, 2014. 24: p. 3–14. 10.4314/ejhs.v24i0.2s PMC4249207 25489179

[14] Probandari A., et al., Barriers to utilization of postnatal care at village level in Klaten district, central Java Province, Indonesia. BMC health services research, 2017. 17(1): p. 1–9. 28784169 10.1186/s12913-017-2490-y PMC5547562

[15] WHO, The Partnership for Maternal, Newborn & Child Health. 2011. Strategic Framework 2012 to 2015. Geneva, Switzerland: PMNCH. 2011.

[16] Campbell M.K., et al., Church-based health promotion interventions: evidence and lessons learned. Annu. Rev. Public Health, 2007. 28: p. 213–234. doi: 10.1146/annurev.publhealth.28.021406.144016 17155879

[17] Anshel M.H. and Smith M., The role of religious leaders in promoting healthy habits in religious institutions. Journal of religion and health, 2014. 53(4): p. 1046–1059. doi: 10.1007/s10943-013-9702-5 23516019

[18] Lumpkins C.Y., et al., Promoting healthy behavior from the pulpit: Clergy share their perspectives on effective health communication in the African American church. Journal of religion and health, 2013. 52(4): p. 1093–1107. doi: 10.1007/s10943-011-9533-1 21965057 PMC3540142

[19] Lasater T.M., et al., Synthesis of findings and issues from religious-based cardiovascular disease prevention trials. Annals of Epidemiology, 1997. 7(7): p. S46–S53.

[20] Peterson J., Atwood J.R., and Yates B., Key elements for church‐based health promotion programs: outcome‐based literature review. Public Health Nursing, 2002. 19(6): p. 401–411. doi: 10.1046/j.1525-1446.2002.19602.x 12406175

[21] Holt C.L., Lewellyn L.A., and Rathweg M.J., Exploring religion-health mediators among African American parishioners. Journal of Health Psychology, 2005. 10(4): p. 511–527. doi: 10.1177/1359105305053416 16014389

[22] Williams R.M., et al., A study of rural church health promotion environments: Leaders’ and members’ perspectives. Journal of religion and health, 2012. 51(1): p. 148–160. doi: 10.1007/s10943-009-9306-2 19960262

[23] Killip S., Mahfoud Z., and Pearce K., What is an intracluster correlation coefficient? Crucial concepts for primary care researchers. The Annals of Family Medicine, 2004. 2(3): p. 204–208. doi: 10.1370/afm.141 15209195 PMC1466680

[24] CSA, Central Statistical Agency (CSA) [Ethiopia] and ICF. 2016. Ethiopia Demographic and Health Survey 2016. Addis Ababa, Ethiopia, and Rockville, Maryland, USA: CSA and ICF. 2016.

[25] Stanback J., et al., Checklist for ruling out pregnancy among family-planning clients in primary care. The Lancet, 1999. 354(9178): p. 566. doi: 10.1016/S0140-6736(99)01578-0 10470704

[26] Bellg A.J., et al., Enhancing treatment fidelity in health behavior change studies: best practices and recommendations from the NIH Behavior Change Consortium. Health psychology, 2004. 23(5): p. 443. doi: 10.1037/0278-6133.23.5.443 15367063

[27] Ayalew T.W. and Nigatu A.M., Focused antenatal care utilization and associated factors in Debre Tabor Town, northwest Ethiopia, 2017. BMC research notes, 2018. 11(1): p. 1–6. 30445991 10.1186/s13104-018-3928-y PMC6240228

[28] Yoseph M., et al., Institutional delivery services utilization and its determinant factors among women who gave birth in the past 24 months in Southwest Ethiopia. BMC health services research, 2020. 20(1): p. 1–10. 10.1186/s12913-020-05121-9 PMC7106731 32228558

[29] Bililign N. and Mulatu T., Knowledge of obstetric danger signs and associated factors among reproductive age women in Raya Kobo district of Ethiopia: A community based cross-sectional study. BMC pregnancy and childbirth, 2017. 17(1): p. 1–7. 28222694 10.1186/s12884-017-1253-4 PMC5320700

[30] Maternal J., neonatal health: Monitoring birth preparedness and complication readiness, tools and indicators for maternal and newborn health. Johns Hopkins, Bloomberg school of Public Health. Center for communication programs, Family Care International, 2004.

[31] Heaman M.I. and Gupton A.L., Psychometric testing of the perception of pregnancy risk questionnaire. Research in nursing & health, 2009. 32(5): p. 493–503. doi: 10.1002/nur.20342 19606451

[32] Lee Y.W., et al., AIMQ: a methodology for information quality assessment. Information & management, 2002. 40(2): p. 133–146.

[33] Petty R.E., et al., The elaboration likelihood model of persuasion. 1986: Springer.<

