# Supplementary material for: Effectiveness of trained religious leaders’ engagement in maternal health education on improving maternal health service utilizations: Protocol of cluster randomized controlled trial in Hadiya Zone, Southern Ethiopia
Source: PLoS One. 2024 Apr 10;19(4):e0296173. doi: 10.1371/journal.pone.0296173 (PMC11006183; doi:10.1371/journal.pone.0296173)
Supplement: S1 Protocol — (DOCX) [file pone.0296173.s002.docx]

**JIMMA UNIVERSITY**

**INISTITUTE OF HEALTH**

**GRADUATE STUDIES**

**EFFECTIVENESS OF TRAINED RELIGIOUS LEADERS’ ENGAGEMENT IN MATERNAL HEALTH EDUCATION ON IMPROVING MATRNAL HALTH SERVICE UTILIZATIONS: CLUSTER RANDOMIZED CONTROLLED TRIAL IN HADIYA ZONE, SOUTHERN ETHIOPIA.**

**By**

**Abinet Arega (MPH/HEHP)**

**A RESEARCH PROPOSAL IS TO BE SUBMITTED TO DEPARTMENT OF HEALTH, BEHAVIOUR AND SOCIETY, FACULTY OF PUBLIC HEALTH, INSTITUTE OF HEALTH, JIMMA UNIVERSITY, IN FULFILMENT OF THE REQUIREMENTS FOR THE AWARD OF THE DEGREE OF DOCTOR OF PHILOSOPHY IN PUBLIC HEALTH/HEALTH COMMUNICATION AND HEALTH BEHAVIOUR.**

**August, 2022**

**JIMMA, ETHIOPIA**

**EFFECTIVENESS OF TRAINED RELIGIOUS LEADERS’ ENGAGEMENT IN MATERNAL HEALTH EDUCATION ON MATRNAL HALTH SERVICE UTILIZATIONS: CLUSTER RANDOMIZED CONTROLLED TRIAL IN MISHA DISTRICT, HADIYA ZONE, SOUTHERN ETHIOPIA.**

**PI:** Abinet Arega (MPH/HEHP)

**Promoters:**

1. Professor. Zewdie Birhanu (Ph.D.)
2. Dr. Yohannes Kebede (Ph.D., Associate Professor)

**Declaration for thesis proposal approval**

I, the undersigned, declare I have agreed to accept the responsibility for the scientific, ethical and technical conduct of this research thesis and provision of the required progress reports as per the terms and conditions of public health and the Jimma University.

Name of student: Abinet Arega

Signature: ___________________________

Date of submission: _________________

As a University advisor, I declare that the proposal is approved for submission for Proposal defence.

Name of first Advisor: **Professor Zewdie Birhanu** (Ph.D.)

Signature: ____________

Date: ___________

Name of second Advisor: **Dr. Yohannes Kebede** (Ph.D., Associate Professor)

Signature: _______________________

Date: ____________________________

# Summary

Background: Despite the many supply- and demand-side interventions aimed at increasing uptake of maternal health service utilizations, the maternal and new-born health service utilizations remains low. Religious leaders have the power to inhibit or facilitate effective adoption of maternal health service utilizations to promote maternal and child health. However, evidence on the roles of religious leaders in promoting maternal health behaviour in developing world is not fully known. Therefore this cluster-randomized trial is designed to evaluate the effects of trained religious leaders’ engagement in maternal health service utilizations and knowledge of major obstetric danger signs.

Methods: A community based cluster randomized control trial in which the study kebeles are randomly assigned into intervention and control groups will be conducted. The sample size is calculated using stata software. Three hundred six pregnant mothers will be enrolled in each group. The intervention comprised of the messages on maternal health and new-born care by trained religious leaders. A baseline study will be conducted before the intervention and post-intervention evaluation will be conducted after four months. Religious leaders will be selected and trained to lead participatory sessions on maternal health and new-born care. Data on maternal health service utilizations, knowledge about obstetric danger signs, attitude towards skilled delivery service utilization and perception of pregnancy risk will be collected from a repeated cross sectional household survey. Effect of intervention will be assessed using multivariable logistic regression with generalized estimating equation model. Mixed-effects multilevel logistic regression model will be used to identify determinants of skilled delivery service utilizations. Data will be analyzed using STATA software. For qualitative study, coded transcripts will be further analyzed and summarized in narratives for each theme and sub-theme.

**Work plan and budget:** the project will take about one year for completion and the total estimated cost is 256,000 Birr.

**Key words:** Religious leaders, maternal health, Hadiya Zone

# ACKNOWLEDGEMENTS

I would like to thank Jimma University Doctoral School and Department of Health, Behaviour and Society for providing me this opportunity to carry out the research proposal development. Prof. Zewdie Birhanu (Ph.D) and Dr. Yohannes Kebede (Ph.D., Associate Professor) for their valuable advice and comments during the development of the proposal. Lastly but not least I would like to extend my thank to my friends for their supports and suggestion and Lemo district and Ameka district Health Offices for giving information.

Table of Contents

[**Declaration for thesis proposal approval** iii](#_Toc117097144)

[Summary iv](#_Toc117097145)

[ACKNOWLEDGEMENTS v](#_Toc117097146)

[List of tables vii](#_Toc117097147)

[List of figures viii](#_Toc117097148)

[List of Acronyms and Abbreviation viii](#_Toc117097149)

[CHAPTER ONE: INTRODUCTION １](#_Toc117097150)

[1.1 Back ground １](#_Toc117097151)

[1.1.2 Definitions of Maternal health and Maternal Healthcare Services １](#_Toc117097152)

[1.1.3 Maternal and New-born Health issues worldwide ２](#_Toc117097153)

[1.1.4 Global Goals on MCH ４](#_Toc117097154)

[1.1.5 Maternal Health Status in Ethiopia ４](#_Toc117097155)

[1.1.6 Statement of the problem ５](#_Toc117097156)

[CHAPTER TWO: LITERATURE REVIEW ７](#_Toc117097157)

[2.1 Antenatal care ７](#_Toc117097158)

[2.2 Skilled delivery care １０](#_Toc117097159)

[2.3 Perception of pregnancy risk １２](#_Toc117097160)

[2.4 Knowledge of the major obstetric danger signs １３](#_Toc117097161)

[2.5 Religious leaders in promoting health １４](#_Toc117097162)

[2.6 Roles of religious leaders in maternal and child health promotion １５](#_Toc117097163)

[2.7 Impact of religious leaders on maternal and child health outcomes １７](#_Toc117097164)

[2.8 Implementation challenges associated with working with religious leaders １９](#_Toc117097165)

[2.9 Conceptual Framework １９](#_Toc117097166)

[2.10 Significance of the study ２２](#_Toc117097167)

[Objectives ２２](#_Toc117097168)

[3.1 General objective ２２](#_Toc117097169)

[3.2 Specific objectives ２２](#_Toc117097170)

[Hypothesis ２３](#_Toc117097171)

[Chapter four Methods and Materials ２３](#_Toc117097172)

[4.1 Study settings and period ２３](#_Toc117097173)

[4.2 Study design ２５](#_Toc117097174)

[4.3 Source population ２５](#_Toc117097175)

[4.4 Study population ２５](#_Toc117097176)

[4.5 Inclusion and Exclusion Criteria ２５](#_Toc117097177)

[4.7 Study variables ２６](#_Toc117097178)

[4.7.1 Dependent variables ２６](#_Toc117097179)

[4.7.1 Independent variables ２６](#_Toc117097180)

[4.8 Sample size ２７](#_Toc117097181)

[4.9 Randomization and Sampling procedure ２７](#_Toc117097182)

[Randomization ２８](#_Toc117097183)

[4.11 Trial design ２８](#_Toc117097184)

[4.12 Sampling techniques ２９](#_Toc117097185)

[4.13 Intervention ３０](#_Toc117097186)

[4.14 Cluster and individual selection ３６](#_Toc117097187)

[4.15 Intervention assignment and masking ３７](#_Toc117097188)

[4.17 Data collection and management ４１](#_Toc117097189)

[4.18 Data Quality Control ４２](#_Toc117097190)

[4.19 Statistical analysis ４４](#_Toc117097191)

[Table 4: Summary of study design ４６](#_Toc117097192)

[4.20 Dissemination plan ４７](#_Toc117097193)

[4.21 Ethical consideration ４７](#_Toc117097194)

[References ５１](#_Toc117097195)

[APPENDICES ５８](#_Toc117097196)

[Annex 1: Questionnaire for quantitative part ６１](#_Toc117097197)

[Annex 2 Amharic Version ７７](#_Toc117097198)

[Annex 2 Interview guide for qualitative study ９２](#_Toc117097199)

[Annex 3 Interview guide for qualitative study Amharic Version ９３](#_Toc117097200)

[Key message Amharic Version ９５](#_Toc117097201)

[Key message Hadiyisa Version ９７](#_Toc117097202)

# **List of tables**

[Table 1 Distribution of religious institutions in selected clusters (kebeles) in Lemo and Amaka districts ３０](#_Toc100565445)

[Table 2 Pregnancy screening questions adapted from Kenya family planning screening questions ３７](#_Toc100565446)

[Table 3: Budget summary ５０](#_Toc100565447)

# **List of figures**

[Figure 1: Religious leaders led SBCC frame work to improve maternal health service utilization. ２０](#_Toc100565448)

[Figure 2: Diagrammatic representation of the randomization of study cluster (kebeles) ２７](#_Toc100565450)

[Figure 3. Presents the intervention processes and package of trained religious leaders led SBCC. ３０](#_Toc100565451)

# List of Acronyms and Abbreviation

ANC: Antenatal care

BPCR: Birth preparedness and complication readiness

CSA: central statistical agency

EDHS: Ethiopian demographic health survey

EmOC: Emergency obstetric care

EPHI: Ethiopian Public Health Institute

FMOH: Federal Ministry of Health

HEWs: Health Extension workers

MCH: maternal and child health

MDG: Millennium Development Goal

PHCU: Primary Health Care Unit

PPRQ: Perception of Pregnancy Risk Questionnaire

SBCC: Social behavioral change communication

SDG: Sustainable development goal

WDA: Women development army

WHO: World Health Organization

#

# **CHAPTER ONE: INTRODUCTION**

## **Back ground**

## Definitions of Maternal health and Maternal Healthcare Services

Maternal health is the health of the mother during pregnancy, childbirth, and the postpartum period. Maternal health care are antenatal care (ANC), skilled delivery care, and postnatal care (PNC) (WHO, 2016).

**Antenatal care**

ANC care is provided by skilled healthcare workers to pregnant women and girls in order to protect the health of both mothers and babies. This is usually accomplished through risk-aversion activities, health education, and the detection and management of pregnancy-related diseases or complications (WHO, 2016). The WHO revised the required number of antenatal contacts from four (under the 2002 focused ANC model) to eight in 2016. Under the eight-contact schedule, women are expected to have their first contact during the first trimester, two contacts during the second trimester and five in the last trimester Components recommended for all women during the eight contacts include items such as nutritional counselling, iron and folic acid supplementation, maternal assessments such as hyperglycaemia tests and a 24-week ultrasound scan, and preventive measures such as the tetanus toxoid vaccination. Prior to the policy change, ANC use was often measured using two indicators: (i) the percentage of women who reported receiving ANC at least once during their previous pregnancy, and (ii) the percentage of women who reported receiving ANC at least four times during their previous pregnancy (WHO, 2010, WHO, 2006). Doctors, nurses, midwives, clinical officers, and community health workers are among the skilled healthcare professionals are trained to offer some components of ANC. (WHO, 2010).

**Skilled delivery care**

There are several definitions for delivery care that incorporate slightly different components. The fraction of births that occur in health facilities is referred to as "institutional births"4 or "institutional deliveries" by the WHO (WHO, 2006). This definition is based on the idea that women giving birth in a health institution were likely attended to by a skilled health worker who was trained to handle simple deliveries and refer complicated ones (WHO, 2006), Despite the fact that this is not always the case. This indicator is also known as facility birth and facility-based delivery (Moyer et al., 2013). Traditional birth attendants, whether or not they have been trained, are not regarded appropriate caregivers for delivery care (WHO, 2010). The proportion of births attended by a skilled health professional is also one of the Sustainable Development Goal indicators related to the third goal pertaining to health and well-being (WHO, 2015). One limitation associated with the different definitions of delivery care is the potential discrepancy between skills that trained health workers should theoretically have and the actual skills they have when practically assessed (Harvey et al., 2007). Additionally, when self-reported responses are relied upon from household survey data in low-resource settings, there are also limitations in women’s ability to distinguish between various health worker cadres (Radovich et al., 2019).

**Postnatal care**

The postnatal or postpartum period is generally considered to cover the six weeks following birth. The WHO recommends four postnatal contacts with the first occurring within 24 hours of birth; additional visits are recommended at 48-72 hours, 7-14 days and six weeks post-delivery (WHO, 2013). Both women and new-borns are at highest risk of death and complications during this period (Warren et al., 2006). PNC guidelines cover care recommended for both mothers and new-borns; assessments of the baby include items such as its ability to feed, its breathing and its temperature. Mothers are typically assessed for bleeding, infection, pain, etc.(WHO, 2013). PNC service use can be measured using several indicators depending on contact timing (i.e., within 24 hours, other contacts or all recommended contacts) and target (i.e. mother or new-born or both). The DHS program measures the percent of women who receive postnatal checks during the first two days from health workers, community health workers or traditional birth attendants. Separate indicators describing the type of provider present for the first PNC check for women and for new-borns are also present. When relying on self-reported responses from household surveys, one of the limitations is the potential overestimation of PNC among women who delivered at a health facility who may not be able to distinguish between intra-partum and postpartum checks by healthcare professionals (Amouzou et al., 2020).

## 1.1.3 Maternal and New-born Health issues worldwide

The health of mothers and children is interrelated and affected by multiple factors (Sharif, 2005). Every year, millions of pregnant women, new mothers, and children suffer from serious sickness or die as a result of preventable or treatable causes (U.N., 2019). Almost all maternal and child deaths (99%) occur in less developed countries, with Africa being the hardest hit region (WHO, 2019a). Maternal and child health (MCH) is receiving more attention, and under-five and maternal death rates have dropped significantly since 1990. Improving MCH is considered as vital to supporting economic development.

Still, as efforts focus on achieving new global MCH goals such as ending preventable deaths among new-borns and children under five and reducing global maternal mortality, significant challenges remain. Despite the availability of effective interventions, lack of finance and limited access to services has slowed progress, notably in the area of maternal health.

Each year, an estimated 5.3 million children under age five – primarily infants – die from largely preventable or treatable causes (U.N., 2019). In addition, approximately 295,000 women die during pregnancy and childbirth each year, and millions more experience severe adverse consequences (WHO, 2019a). These challenges are especially prevalent in developing countries. Furthermore, sub-Saharan Africa is the hardest hit region in the world, followed by Southern Asia and South-Eastern Asia; altogether they account for approximately 90% of maternal and under-five deaths (U.N., 2019).

More than a quarter (27%) of all maternal deaths is due to severe bleeding, mostly after childbirth (postpartum haemorrhage). Sepsis (11%), unsafe abortion (8%), and hypertension (14%) are other major causes. Maternal deaths are caused by diseases that complicate pregnancy, such as malaria, anemia, and HIV, which account for around 28% of all maternal deaths (Say et al., 2014). Inadequate care during pregnancy and high fertility rates, often due to a lack of access to contraception and other family planning/reproductive health (FP/RH) services increase the lifetime risk of maternal death. While the proportion of pregnant women who receive the required minimum of four prenatal care visits is increasing, it is still only 52% in developing countries and even lower in Sub-Saharan Africa and Southern Asia (UN, 2015a).

Complications due to premature births account for more than a third (35%) of new-born deaths, followed by delivery-related complications (24%), sepsis (15%), congenital abnormalities (11%), pneumonia (6%), tetanus (1%), diarrhoea (1%), and other causes of death (7%) (U.N., 2019). Low birth weight is a major risk factor and indirect cause of new-born death (Black et al., 2010). New-born deaths account for most child deaths (47%), followed by pneumonia (12%), diarrhoea (8%), injuries (6%), malaria (5%), measles (2%), HIV/AIDS (1%), and other causes of death (21%) (U.N., 2019).

Key interventions that reduce the risk of maternal mortality include skilled care at birth and emergency obstetric care. New-born deaths may be substantially reduced through increased use of simple, low-cost interventions, such as breastfeeding, keeping new-borns warm and dry, and treating severe new-born infections. Strengthening health systems and improving access to treatments, notably through community-based clinics, are also critical, and interventions that are integrated into a comprehensive continuum of care have been found to be more effective (WHO, 2011).

## 1.1.4 Global Goals on MCH

The following are some of the most important global goals for increasing access to and improving MCH services:

**SDGS 2 & 3: SAVE MOTHERS AND CHILDREN’S LIVES AND END ALL FORMS OF MALNUTRITION**

Global MCH targets were adopted in 2015 as part of Sustainable Development Goals (SDGs) 2 and 3 and are to, by 2030 (UN, 2015b):

Goal 3. Ensure healthy lives and promote wellbeing for all at all ages

3.1 By 2030, reduce the global maternal mortality ratio to less than 70 per 100,000 live births

3.2 By 2030, end preventable deaths of new-borns and children under 5 years of age, with all countries aiming to reduce neonatal mortality to at least as low as 12 per 1,000 live births and under 5 mortality to at least as low as 25 per 1,000 live births.

## 1.1.5 Maternal Health Status in Ethiopia

Levels of utilization of ANC, delivery and PNC have been steadily increasing in Ethiopia as
shown by DHS data between 2000 and 2019. The national averages in 2000 were 27% for ANC, 5% for delivery care and 11% for PNC (CSA, 2001) but increased to 74%, 50% and 34% respectively by 2019 (EPHI, 2019). Similar upward trends have been observed in the SNNP region, with ANC having the highest consumption and PNC having the lowest. While ANC use, when measured as at least one visit, has generally been higher than other service use in Ethiopia, it is much lower when the proportion of women reporting four or more visits is considered. Nevertheless, the number of women reporting at least four ANC contacts has increased between 2000 (10%) and 2019 (43%) (EPHI, 2019, CSA, 2001). Pregnant women have also tended to begin using ANC in their second trimester rather than in their first trimester as recommended, and this has only marginally improved over time (CSA, 2016). The proportion of births attended by skilled professionals has been on the rise since 2000 (CSA, 2016, CSA, 2001). However, the majority of these births are assisted by nurses and midwives rather than doctors. In 2016 for example, only 20% of births were attended by doctors although the proportion is much higher in private facilities compared to government facilities (CSA, 2016). A shift in policy around clean and safe delivery at health posts may also have affected the profile of providers attending to births. Initially HEWs were trained to support uncomplicated deliveries at health posts (Medhanyie et al., 2012, Jackson and Hailemariam, 2016), but this policy was later discontinued possibly over concerns about the inability of all HEWs to perform this safely (Desta et al., 2017). The percentage of women receiving PNC from health professionals has also been slowly rising from just 3% in 2000 to 16% in 2016 (CSA, 2001, CSA, 2016). Most women receive their first postnatal check within the first 48 hours after delivery and numbers have been steadily increasing from just 2% in 2000 (49) to 34% in 2019 (EPHI, 2019). As with ANC, very few women have additional PNC visits beyond the first 48 hours as recommended. In fact in 2016, less than 2% of women reported postnatal checks between one and six weeks after birth (CSA, 2016).

## 1.1.6 Statement of the problem

The maternal mortality rate is far too high. In 2017, over 295 000 women died during and after pregnancy and childbirth. The overwhelming majority of those deaths (94%) occurred in low-resource settings, and most could be prevented. In 2017, maternal fatalities in Sub-Saharan Africa and Southern Asia accounted for over 86 percent (254 000) of all maternal deaths worldwide. Sub-Saharan Africa alone accounted for roughly two-thirds (196 000) of maternal deaths, while Southern Asia accounted for nearly one-fifth (58 000) (WHO, 2019b).

High mortality rates for pregnant women and their new-borns are one of Africa's most intractable public health issues today, and Ethiopia is one of the countries most afflicted. The most direct causes of maternal death in Ethiopia include obstetric complications like haemorrhage, obstructed labor/ruptured uterus, pregnancy-induced hypertension, puerperal sepsis, and unsafe abortion. the foremost reported indirect causes of maternal death were anaemia and malaria (Mekonnen and Gebremariam, 2018).

Since 2000, Ethiopia has reduced maternal and child mortality by half, but a maternal death rate of 412 per 100,000 live births and child death rate of 67 per 1,000 are still too high. Impressive progress has been made with half women now parturition during a facility, reducing the danger of death thanks to complications during delivery (USAID, 2020). Ethiopia has achieved a big progress on reduction of maternal and child mortality within the Millennium Development Goal (MDG) era, and also left issues and lessons within the era of post-2015 (CSA, 2016, CSA, 2012). The government of Ethiopia has taken maternal and neonatal health and underlying accessibility and quality of healthcare under consideration , with the worldwide targets of the Sustainable Development Goals (SDGs) to scale back the maternal mortality ratio to 70 per 100,000 live births and to scale back the neonatal mortality rate  to as low as 12 per 1,000 live births by 2030 (Ahmed et al., 2010, WHO, 2015)

One explanation for top maternal death rates and poor health outcomes among women in Ethiopia is underutilization of maternal health services (antenatal, skilled birth and postnatal care) by an outsized proportion of women within the country (Mekonnen and Mekonnen, 2003, Berhan and Berhan, 2014). Facility- based maternal health services are a proximate determinant of maternal morbidity and mortality (Probandari et al., 2017) 4 and proven health interventions for preventing maternal morbidity and mortality, mainly among women in resource-poor settings (Probandari et al., 2017).

As a result, expanding access to and usage of expert maternal care during pregnancy, birth, and postpartum is critical, particularly in an enabling environment with sufficient or appropriate facilities (Campbell et al., 2006, Clark, 2012). Antenatal care (ANC) plays a critical role in reducing maternal deaths by detecting and managing pregnancy-related problems early (AbouZahr and Wardlaw, 2003). However, not all causes of maternal death are often averted by ANC, especially those resulting from complications arising during birth and therefore the immediate postpartum period (Ronsmans et al., 2006). Hence, subsequent skilled birth and postnatal care (PNC) are essential within the continuum of maternal health service. Skilled birth care can avert most maternal deaths that occur round the time of birth through active management of the third stage of labour and administering uterotonic medicines to scale back haemorrhage (Prata et al., 2013). PNC has the potential to scale back maternal deaths through identification and treatment of post-partum complications12 and enable doctors to supply health promotion also as preventive interventions (Lawn et al., 2010).

According to the EDHS 2016, the Central Statistics Agency, 80 percent of births in cities were assisted by a skilled provider, and 79 percent were delivered in the health facilities, compared to 21% and 20%, respectively, of births in rural areas (CSA, 2016). Although, the Federal Ministry of Health (FMOH) has applied a multi-prolonged approach to extend utilization of institutional delivery by improving access and strengthening facility-based maternal services but, the proportions of births attended by skilled personnel is merely 28%, which is extremely much less than that of the SSA that was around 53% (Alemi Kebede and Teklehaymanot, 2016, Eshete et al., 2019). The share of skilled birth attendant in Ethiopia is one among rock bottom within the world.

Community education and demand generation activities by involving family members, traditional and religious leaders in maternal health behaviours are potential solutions. Knowledge about maternal and neonatal health service utilization can be increased through community-based structures, such as religious institutions. In Ethiopia, the majority of the population belongs to a religious group, and religion is an important part of society. Through places of worship, religious institutions have access to a large portion of the population. Religious leaders serve as community leaders as well as gatekeepers of information and access to the population and can help raise awareness about underutilized maternal and neonatal health services.

Therefore this cluster-randomized trial is designed to evaluate the effects of trained religious leaders’ engagement in maternal health education versus usual care on prenatal behaviours. We hypothesize that trained religious leaders’ engagement in maternal health education will increase the proportion of antenatal care, skilled delivery service and postnatal care service utilizations.

# CHAPTER TWO: LITERATURE REVIEW

## 2.1 Antenatal care

A systematic review of the factors associated with ANC use in Sub-Saharan Africa (SSA) identified 74 observational studies conducted between 2008 and 2018 (Okedo-Alex et al., 2019). About one-third of the studies originated in Ethiopia. The majority of studies focused on individual and household-level factors. Higher education levels for women and their husbands, higher wealth, being employed, lower parity, planned pregnancies, having health insurance, living close to facilities, urban residence and being involved in decisions generally all exhibited favourable associations with ANC use. The review described the association between age and ANC use as context dependent with some studies reporting higher odds of use among older women and others finding higher odds of use among women under 20 years of age (Okedo-Alex et al., 2019). Ownership or use of radio and televisions was also positively correlated with ANC use in a handful of studies. A few studies specifically examined community-level factors (Makate and Makate, 2017, Ononokpono et al., 2013). For instance, a study from Zimbabwe reported that community-level prevalence of contraceptive use positively correlated with increased odds of ANC use in both urban and rural populations; religious composition of the community and more nurses per capita were significantly associated with higher odds of ANC use among rural women but not urban women (Makate and Makate, 2017). Another study used DHS data from 32 low- and middle-income countries (LMICs) to explore determinants of the frequency of ANC use to see if they differed from those linked to any ANC use (Guliani et al., 2014). The authors reported education, age, marital status, household wealth and place of residence (rural or urban) as influencing both use and frequency of antenatal care. With respect to ANC use, education and household wealth showed almost “dose-dependent” associations with ANC attendance with increasing levels correlating with higher odds of use. In terms of frequency of ANC use, women with primary education were likely to have 11% more visits than women with no education, while those with a secondary education were likely to have 37% more visits. Married women had higher odds of ANC use than unmarried women as did employed women compared to unemployed ones. Women having their first child were most likely to seek ANC compared to women with higher parity while those with unwanted pregnancies had lower odds of ANC use than women who wanted their pregnancy. The interaction between urban residence and wealth indicated that the gap in ANC use between urban and rural residents widened as wealth increased. For instance, urban women in the least poor quintile had double the odds of ANC use compared to their rural counterparts. The authors also explored differences in the relative influence of factors between countries in Africa, Asia and Latin America, noting consistency in the effects of education, marital status, unwanted pregnancies and, wealth on ANC use across all three continents. The effects of employment varied, exerting a negative effect on frequency of ANC visits in Asia but a positive effect in SSA (Guliani et al., 2014).

A systematic review focusing on factors associated with women completing at least one ANC visit in Ethiopia included 15 community-based, observational studies conducted between 2002 and 2016 (Tekelab et al., 2019). The authors pooled adjusted estimates for place of residence, education levels of women and their husbands and, whether or not the pregnancy was planned using a random-effects model. Higher odds of having at least one ANC visit were associated with urban residence, having some level of education, having a husband with some level of education and a pregnancy being planned or intentional. Eight studies investigated the association between women’s age and ANC use with five indicating that younger women had a higher odds of ANC use (Jira and Belachew, 2005, Zelalem Ayele et al., 2014, Abosse et al., 2010). A few studies identified in the review considered geographical barriers such as travel time (Dutamo et al., 2015, Amentie et al., 2015) (71,74) or estimated distance between household clusters and the nearest health facility (Girmaye and Berhan, 2016, Worku et al., 2013) as potential factors influencing ANC use. In general, longer travel times or larger distances were associated with lower odds of ANC use. Another study in Ethiopia showed that higher educational attainment, higher socio-economic status, exposure to mass media, and self-reporting decision empowerment were significantly associated with having at least four ANC visits (Ousman et al., 2019). Multivariate analyses in Ethiopia showed that higher maternal and paternal education, higher household wealth status, urban residency and previous use of a contraceptive were associated with ANC service use (1–3 and 4+ ANC visits) (Mekonnen et al., 2019).

In many societies, cultural beliefs and lack of awareness inhibit preparation for delivery and seeking care. Due to this reason complications occur in unprepared family could take lots of time in understanding the problem; to get organize, in getting money, in finding transport and reaching the appropriate referral facility. Therefore, this delay in the decision, in reaching health facility and delay in receiving care can be solved by proper use of birth preparedness and complication readiness plan (Acharya et al., 2015, Organization, 2002). Birth preparedness and complication readiness (BPCR) helps pregnant women how to identify skilled birth attendant, how to identify signs of labor and in recognizing danger signs for pregnancy-related complications. It also helps to reduce other barriers to seeking care, such as transport costs, perceptions of poor quality of care and cultural differences (Organization, 2007). BPCR components are included in the new World Health Organization (WHO) model for antenatal care as part of antenatal care education in clinic setting (Villar et al., 2001). Birth Preparedness and Complication Readiness is one of the strategy to promote timely utilization of skilled maternal and neonatal care timely. Preparing for childbirth and associated complications reduces the three delays of maternal death; delay in recognizine danger signs and decision of seeking care in home, delay in reaching health facility and delay in receiving health care by encouraging pregnant women, their families, and communities to effectively plan for births and prepare for emergencies if they occur (Botha et al., 2013).

## 2.2 Skilled delivery care

An analysis of DHS data from 43 countries in Africa and Asia from over 200,000 women identified several factors that significantly correlated with using delivery care (Diamond-Smith and Sudhinaraset, 2015). In terms of individual characteristics, higher age, more years of education, not being married and not wanting the pregnancy was associated with increased odds of facility delivery. At household level, wealth, place of residence, sex of household head, having fewer children, husband’s age, husband’s employment status but not husband’s education was important correlates of delivery care use. Living in a community with a high proportion of women using ANC services at least four times as well as using ANC herself was also associated with increased odds of delivery care use. When only African countries were considered, important correlates remained largely similar; however, being unemployed and having husbands with more education were positively associated with delivery care use. Unlike the model including all countries, sex of household head and husband’s age did not exhibit statistically significant associations in models with African countries only (Diamond-Smith and Sudhinaraset, 2015).

The context-dependent influence of husband’s education was also reported in another smaller scale analysis using DHS data between 2006 and 2010; while in Nigeria women with more educated husbands (secondary versus primary education) had higher odds of delivery care, the opposite was true in Kenya, but no effect was observed in Tanzania (Tey and Lai, 2013). Reported frequency of media use (radio, television and newspaper use combined) also had country dependent associations; lower use scores were associated with lower odds of facility delivery in Kenya and Nigeria but not Tanzania, however, distance is likely to play an important role in women’s ability to access delivery care (Bohren et al., 2014). Indeed, the most commonly reported reasons for not delivering at a health facility reported in Kenya and Tanzania were large distances and lack of transport (Tey and Lai, 2013).

A meta-analysis of factors associated with delivery care use in Ethiopia was recently published. It included 24 observational studies, primarily of cross-sectional design, that were conducted mainly in Amhara and Oromia regions (Nigusie et al., 2020). The authors reported abstracting adjusted effect estimates to generate pooled estimates of association for women’s attitudes, women’s knowledge, age at first pregnancy, education, parity, occupation, place of residence, availability of information sources, distance, ANC use, place of most recent birth and complications during prior birth with delivery care use (Nigusie et al., 2020). Women with a favourable attitude towards delivery care services, who were younger than 35 years of age at first pregnancy, who were literate or had some level of formal education, lived in urban areas, had used ANC services and had given birth to their previous most recent child at a health facility had higher odds of using delivery care. Another earlier meta-analysis on factors associated with use of delivery care in Ethiopia reported unadjusted pooled summary estimates using 34 observational studies published between 2000 and 2014 (Alemi Kebede and Teklehaymanot, 2016). In general, younger age, first-time pregnancies, higher education among women and their husbands, urban residence, better danger sign awareness, ANC use, planned pregnancies and the presence of complications during pregnancy favoured the use of delivery care services among women.

Another study has revealed that place of residence, maternal education, and husband and relatives decision are found to be very important for institutional delivery. The presence of professional individuals at birth is critical in guaranteeing the safe delivery of women and thereby lowering maternal mortality; unfortunately, the majority of our moms still give birth at home without the support of a skilled person (Fikre and Demissie, 2012).

A systematic review and META-analysis in Ethiopia found that there are several modifiable factors such as empowering women through education; promoting antenatal care to prevent home delivery; increasing awareness of women through mass media and making services more accessible would likely increase utilization of institutional delivery (Nigusie et al., 2020).

A pooled research based on Demographic and Health Surveys conducted in 12 East African nations from 2008 to 2017 revealed that competent birth attendance at birth was low in the region. Age, women and husband education, wealth index, ANC visit, number of gestation, accessing health care, residence, and living countries were major determinants of skilled birth attendance (Tessema and Tesema, 2020).

## 2.3 Perception of pregnancy risk

Risk perception is defined as the people's judgments and evaluations of risks they might be exposed to (Finucane and Holup, 2006). Risk perception plays a central role in many health behavior theories including, Health Belief Model, Protection Motivation Theory and Prospect Theory (Bayrampour et al., 2012). It seems that a greater perception of health risk increases the protected motivation, it is important to understand how people perceive health risks, how accurate these perceptions are, and how risk information is received (Jackson et al., 2006). Perception of risk is a factor which strongly influences the care that high risk women receive during their pregnancy and their decisions about prenatal care.

A recent meta-analysis by (Brewer et al., 2007) found a high degree of consistency and strength of association between risk perceptions and health behavior, using the example of vaccination. All three risk perception measures were related to vaccination behavior, with perceived risk likelihood (r ¼.26) and susceptibility (r ¼.24) having a moderate effect size and perceived risk severity (r ¼.16) having a small to moderate effect size.

The role of risk perception is an important issue especially during pregnancy when health
behavioral changes have the potential to impact both mother and infant(Bayrampour et al., 2013, Czerwinski et al., 2010) (Bayrampour et al., 2012b; Czerwinski et al., 2010; Kim et al., 2007). Risk perception in pregnancy is a topic that has generated a great deal of interest. A recent concept analysis found that risk perception in pregnancy influences women’s affective state and has an impact on decision-making about pregnancy and childbirth (Lennon, 2016). The manner in which pregnant women conceptualize risk, how women respond to communications regarding pregnancy risk and to what extent these thoughts and beliefs impact health behaviors are all important topics for consideration. Risk perception in pregnancy is a complex phenomenon as it involves not only the individual woman, but the unborn infant as well. It is important for professionals involved in the prenatal care to understand the knowledge of women about the perception of risk, without such an understanding even well intended policies may not be effective (Lee et al., 2012, Slovic et al., 2013)

## 2.4 Knowledge of the major obstetric danger signs

In developing countries, several factors are reported to hamper access to EmOC. The most frequently reported deterrents include socio-economic and cultural factors (Agunwa et al., 2015, Ossai and Uzochukwu, 2015), lack of knowledge about obstetric danger signs (Amenu et al., 2016, Demissie et al., 2015) and poor awareness of the availability of EmOC service (Kabakyenga et al., 2011). Lack of knowledge about obstetric danger signs often results in delays in seeking timely obstetric care (Thaddeus and Maine, 1994). In Ethiopia, it has been indicated that only 6% of women who encountered obstetric complications were able to access EmOC (Admasu et al., 2011), with the main reason being lack of knowledge about obstetric danger signs.

Knowledge of the major obstetric danger signs, including severe vaginal bleeding, oedema on the face, blurred vision, prolonged labour, convulsions, retained placenta, foul-smelling vaginal discharge, and high grade fever (JHPIEGO, 2004), can help to facilitate timely healthcare access. In previous research in developing countries, it has been suggested women’s knowledge about obstetric danger signs determines their health-seeking behaviour. For instance, Kosum et al. reported that women with poor knowledge of obstetric danger signs are less likely to attend a healthcare facility when they face obstetric emergencies (Koşum and Yurdakul, 2013). Similarly, Jammeh et al. stated that an inability to identify danger signs during pregnancy by women was reported to result in delays in accessing obstetric care(Jammeh et al., 2011). Several researchers have indicated that women with poor knowledge of obstetric danger signs are less likely to have better birth preparedness and complication readiness, and as a result they usually delay seeking appropriate obstetric care (Tilahun and Sinaga, 2016, Zepre and Kaba, 2017, Kabakyenga et al., 2011). At the global level, women’s knowledge about obstetric danger signs has been related to a number of factors. Women who experienced obstetric complications during the previous pregnancies (Rashad and Essa, 2010) (Metwally et al., 2015) are more knowledgeable about obstetric danger signs as compared to those who never experienced obstetric complications. Being exposed to health education (Bintabara et al., 2017) (Gebrehiwot et al., 2014, Pembe et al., 2009) was also reported to improve women’s knowledge about obstetric danger signs. In numerous studies, it has been shown that multiparous (Rashad and Essa, 2010, Pembe et al., 2009) and those women who visited a health facility for antenatal care (ANC) (Rashad and Essa, 2010, Nithya et al., 2017, Doctor et al., 2013) are more likely to be aware of obstetric danger signs as compared to nulliparous and those who did not visit a health facility for ANC. Maternal socio-economic factors are also reported in several studies as affecting women’s knowledge about obstetric danger signs. Women who are employed and work for paid jobs (Bintabara et al., 2017, Okour et al., 2012, Doctor et al., 2013) are more knowledgeable about obstetric danger signs compared to their unemployed counterparts.

The result of numerous studies showed that older mothers (Pembe et al., 2009) and educated mothers (Ossai and Uzochukwu, 2015, Rashad and Essa, 2010) are more knowledgeable about obstetric danger signs than their younger and uneducated counterparts. Morhason-Bello and colleagues, in their study of Nigeria, showed that the Islamic religion (Morhason‑Bello et al., 2016) was associated with better knowledge of obstetric danger signs.

In Ethiopia, similar socio-demographic determinants of women’s knowledge about obstetric danger signs have been reported. In the findings of several studies, it has been shown that educated mothers (Amenu et al., 2016, Tilahun and Sinaga, 2016), older women (Hailu et al., 2010, Workineh et al., 2014) and employed women (Dile et al., 2015) have relatively better knowledge than the uneducated, younger and unemployed mothers. Better knowledge of obstetric danger signs was also reported among women who have an educated partner (Abiyot et al., 2014), have higher household income (Amenu et al., 2016) and are urban dwellers (Fisseha et al., 2017). Women who previously gave birth at a health facility, those with higher number of parities and those who visited a health facility for ANC services in previous pregnancies (Amenu et al., 2016, Tilahun and Sinaga, 2016, Dile et al., 2015, Yadeta and Kumsa, 2017) were reported to have comparatively better knowledge about obstetric danger signs.

## 2.5 Religious leaders in promoting health

Religious and faith community health promotions have the ability to reduce health inequities, and religious institutions are among the most respected and trustworthy institutions that can considerably boost public health work (Campbell et al., 2007). Similarly, (Anshel and Smith, 2014) Faith leaders, in addition to physicians and health care providers, are another category of people who have a big impact on other people's beliefs, feelings, and behaviours, according to research. Spiritual leaders have the ability to impact health behaviour on a variety of levels, from personal to ecological, with "knock-on" implications on community health. This is achieved via health education and health promoting strategies (Lumpkins et al., 2013). Furthermore, the influence of faith leaders on health behaviour is in line with the tenets of the Ottawa Charter for Health Promotion since they are perceived as strengthening community action (Lumpkins et al., 2013). That is, communities must be empowered to participate in and govern their own affairs. A faith leader is in a unique position to encourage behaviour change since he or she is a vital component of the community. According to an evaluation of programs involving faith-based organizations, clergy were able to considerably aid behaviour change, particularly among hard-to-reach populations (Anshel and Smith, 2014, Lasater et al., 1997). Other studies have also identified the importance of faith leader's influence on health behaviour (Peterson et al., 2002). Similarly, faith leaders influence on behaviour has been attributed to Scripture-based passages that espouse the virtues of healthy living (Anshel and Smith, 2014, Holt et al., 2005). "The body is the temple of God," according to one scripture verse that was a mainstay in the above-mentioned research, and so must be kept holy by abstaining from hazardous behaviours. Although some studies have suggested that spiritual leaders play a role in influencing congregants' health behaviours, little information on the amount of this influence and the mechanisms involved available. (Williams et al., 2012).

## 2.6 Roles of religious leaders in maternal and child health promotion

The most commonly performed roles by religious leaders and faith communities in MCH programmes included: health education, psychosocial support, linkage to care and defaulter tracing. In performing these roles, religious leaders and faith communities carry out several activities such as encouraging women to bring their babies back for immunization and treatment and increasing the utilization of family planning.

**Health education:** religious leaders and faith communities were reported to play a crucial role in maternal and child health education; studies stated that health education was a key component of the intervention delivered by religious and faith communities (Plus., 2017, Lau et al., 2020, Georgetown University, 2011).

**Change agents for shaping norms and influencing behaviours:** Socio-cultural factors have been shown to be important determinants of MNCH behaviours, including ANC attendance (Ahmed et al., 2010, Grede et al., 2014, Pell et al., 2013, Simkhada et al., 2008, Underwood et al., 2014). Studies demonstrated that training and mobilising faith-based and religious leaders resulted in positive changes to the timing and frequency of ANC behaviours for pregnant women in rural underserved communities (Hembling et al., 2017, Adedini et al., 2018, Patterson, 2007). Moreover, working with religious leaders and faith community in maternal and child health area shapes the existing norms that can be obstacle for utilization of maternal and child health services. Also study revealed that uptake of family planning use could be increased by working with religious leaders and faith community (Foulkes, 2014, Grabenstein, 2014, Report, 2009). Studies showed that women delivering at faith-based institutions had more antenatal care, antenatal complications, and caesarean delivery. Faith-based institutions had higher obstetrician attendance and lower rates of eclampsia, preterm birth, stillbirth, Apgar_7, and neonatal near miss (Vogel et al., 2012)

**Increasing family planning demand and trust and advocacy**: faith-based organizations-managed facilities and faith leaders to increase family planning demand and service provision, as well as the importance of coordination with the public sector to ensure supply of commodities and support for faith-based organizations -managed facilities. People have a high degree of trust in FBOs and religious leaders and those religious leaders are thus well placed to address the unmet need for family planning. Religious leaders are committed to advocating to public officials about FP and to talking about FP with communities, provided that their teachings and beliefs on FP are respected. Religious leaders are natural advocates for health issues, given their position of caring for their congregations and their connections within communities. They are respected by public officials and within communities, giving credence to their advocacy (Ruark et al., 2019, Bormet et al., 2021, Georgetown University, 2011). Engaging church leaders in family planning promotion demonstrates the efficacy of this strategy as one of several required to increase contraceptive usage and reduce new-born and maternal mortality in poor nations (Bormet et al., 2021) (Foulkes, 2014).

**Changing attitudes:** working with religious leaders and faith community positively associated with family-planning method approval, which was—in turn—positively and significantly associated with the number of positive family-planning attitudes (Underwood et al., 2013). The collaboration and networking of the government and the churches in the health sector is crucial with regards to sexual and reproductive as well as maternal health. In addition, religious leaders are influential in the communities and societies and they can, therefore assist in addressing issues of maternal health in the communities they are serving (Dialogue, 2014) (Chilongozi, 2017).

**Discouraging early marriage**: The study found that faith leaders play essential roles in rationalizing or discouraging early marriage through preaching and other activities (Amzat, 2020).

**Influencing cultural factors**: Faith-based organizations’ close links to communities provide them with an opportunity to promote behaviour change and address other cultural factors contributing to maternal mortality rates such as early marriage and family planning. Working in collaboration with religious leaders and faith community and other stakeholders is critical to promoting demand for maternal and reproductive health services; however, there is limited knowledge about faith-based maternal healthcare and FBOs are often left off the global health agenda (Ostrowski, 2011).

**Health promoters:** faith community and religious leaders have an important role in promoting acceptability of critical health interventions to save lives and improve maternal health as was witnessed in the IIUM HPPMM project MCH promotion (Maiwada et al., 2016, Canada, 2020).

## 2.7 Impact of religious leaders on maternal and child health outcomes

**Improved MCH knowledge, retention in care and uptake of services:** Data on retention in care and utilisation of maternal and child health services was reported in some studies. For

instance, working with faith communities increased uptake of maternal and child health services utilizations and knowledge scores (Ostrowski, 2011) (Patterson, 2007, Vogel et al., 2012). Another review study showed that engagement of religious leaders increased contraceptive use prevalence and decreased infant and maternal mortality in developing countries (Foulkes, 2014, Dialogue, 2014, Grabenstein, 2014, Olivier, 2016).

In Uganda religious leaders received behaviour change communication (BCC) materials to use for raising awareness as well as data collection tools to use to record the number of participants in their awareness raising activities. Over the course of the four-year program, the trained religious leaders took advantage of numerous chances to raise awareness about maternal and reproductive health issues in their communities and places of worship using the information and skills acquired during training. They delivered messages on male involvement, birth spacing, birth preparation, and use of antenatal care and maternity services this working with religious leaders brought improvements in health-seeking behaviours among community members. Women with maternal health issues started opening up to religious leaders and as a result were able to obtain referrals to health facilities. Participants felt that birth preparedness and use of skilled care increased (Plus., 2017) maternal and child health improvement (Canada, 2020).

Interventions that include clergy of various faiths as change agents for establishing norms and teaching behaviours about family planning and contraceptive usage are critical for improving contraceptive adoption, according to a study in Nigeria (Adedini et al., 2018). Religious leaders, in particular, can substantially influence and shape people’s ideas and views about issues such as contraceptive adoption and family formation. Similarly, a larger number of religious leaders with evidence-based message that is consistent with their religious beliefs, who are supported by their denominational leadership and faith-based technical counterparts, and who are given resources to cover their costs, could have a huge and long-term impact on local and national health policy. Most African Christian leaders and groups support modern methods of voluntary FP to achieve healthy timing and spacing of pregnancy and the ultimate goal of ensuring healthier mothers, children, and communities (Bormet et al., 2021, Georgetown University, 2011).

**Modification of harmful norms and practices that limited uptake of ANC services:** the study results point to the potential of mobilising faith-based and lay leaders in promoting early ANC. Study shows how faith leaders' involvement can have an impact on a very specific health-care issue – going beyond the conventional claim that church leaders are "possibly influential" in development in general (Hembling et al., 2017).

**Lead to deeper understanding of reproductive health and enhancing access to voluntary family planning**: Culturally appropriate training for religious leaders lead to a deeper understanding of, and appreciation for, reproductive health and family planning, with the effects manifesting as increased preaching and/or counselling about these important topics (Underwood et al., 2013, Ruark et al., 2019, Barot, 2013). Improving maternal care (Johnson and Wall, 2014, Report, 2009) There was an improved service statistics related to maternal and reproductive health in the intervention area as compared to the non-intervention or control site. Faith-based organizations and groups had an important role in promoting acceptability of critical health interventions to save lives and improve maternal health as was witnessed in the IIUM HPPMM project (Maiwada et al., 2016).

## 2.8 Implementation challenges associated with working with religious leaders

Participating religious leaders found it hard to conduct health promotion activities in remote areas. Some noted that they require fuel and vehicle maintenance to enable them to reach community gatherings; however, in most cases, they do not have the means. Study participants expected the project to pay them - not understand the language used in the BCC materials that were distributed -too expensive to provide transport and lunch allowances-shortage of resources (Plus., 2017). As the study was based on self-reported data, respondents may have answered questions with the aim of pleasing the interviewer, thus adding social desirability bias to the results (Adedini et al., 2018).

Community members complained that religious leaders were talking about family planning inappropriately and that they were straying from their mission of preaching the word of God. Myths and misconceptions about contraception, religious hostility to family planning, Religious leaders were unable to answer technical queries from family planning clients or potential clients. Male Muslim leaders are unable to reach out to women with messages about family planning (Ruark et al., 2019).

The general consensus among the religious leaders was that attitudes toward these MNCH, SRHR, and gender equality messages often varied between older and younger people and also between men and women (Canada, 2020). Because religious leaders are sought by their communities for advice on almost every aspect of daily life, including reproductive health issues, they need scientific and updated information, which prepares them to correct misconceptions, dispel rumours, and provide useful advice (Report, 2009).

A particular challenge for partnerships between secular development organizations and FBOs is the perception that FBOs are weak in key areas of global health implementation, especially program management, monitoring and evaluation (Georgetown University, 2011). Advocating for FP with religious leaders necessitated far more relationship development and a keen understanding of terminology (Bormet et al., 2021).

## 2.9 Conceptual Framework

Figure 1 shows the framework for improving maternal health behaviour using trained religious leaders in maternal health promotion. This conccptual framework is adapted by reviewing different literatures. Trained religious leaders are expected to improve understanding of pregnant mothers on key maternal health behaviour including ANC, SDS, BPCR and knowledge of obstetric danger signs. The religious leaders’ maternal health behaviours messaging, in addition to the existing services, it is expected to increase pregnant mothers access to maternal health information. This will influence maternal health behaviours that will result in improved maternal health service utilizations and knowlwdge and recognition obstetric dangers signs.

Figure 1: Trained religious leaders led maternal health communication and behavioural change process frame work to improve maternal health service utilization.

**Change: psychosocial**

- Knowledge
- Attitude
- Perception of Pregnancy Risk
- Self-efficacy

**Change: practice**

- ANC utilization
- Skilled delivery service utilization
- BPCR

**Trained religious leaders led maternal health education.**

- Group education approach
- IEC materials; flip chart, card and poster
- Case study
- Monitoring and follow up
- Credibility and popularity of trained religious leaders
- Completeness and accuracy of message

**Socio-demographic and economic characteristics**

- Age
- Ethenicity
- Religion
- Income
- Marital status
- Educational status
- Occupation
- Family size
- Average distance from nearest HC

**Reproductive history of pregnant mothers**

- Parity
- Age at pregnancy
- Still birth
- Abortion
- Home delivery
- Institutional delivery
- Neonatal death

## 2.10 Significance of the study

The government of Ethiopia has taken maternal and neonatal health and underlying accessibility and quality of healthcare into account, with the global targets of the Sustainable Development Goals (SDGs) to reduce the maternal mortality ratio to less than 70 per 100,000 live births and to reduce the neonatal mortality rate to as low as 12 per 1,000 live births by 2030. But the proportion of pregnant women who have care during delivery is universally lower than those who receive ANC. Poor, rural women in sub-Saharan Africa and South Asia are the least likely to receive antenatal, delivery or PNC. Different studies on factors influencing the utilization of maternity health services were done in the country and abroad. In Ethiopia, underutilization of the existing maternal health service is a major problem. This study is designed to use religious leaders to empower the community with knowledge and skill for better utilization of existing service for improvement of maternal and new-born health. It is anticipated that the study will provide impetus for the mainstreaming of involvement of religious leaders in maternal and new-born health. It is hoped that future programmes will re-design the current involvement of religious leaders in maternal and new-born health.

# Objectives

## 3.1 General objective

The overall goal of this study is to develop understanding of contributions of religious leaders to maternal health promotion and evaluate effectiveness of trained religious leaders’ engagement in maternal health education versus usual care on maternal health service utilizations. The details of study design, sample size, sampling technique and sampling procedure for each specific objective are discussed in method section.

## 3.2 Specific objectives

**Objective 1**: To assess effectiveness of trained religious leaders’ engagement in maternal health education in improving knowledge and recognitions of danger signs during pregnancy and child birth.

**Objective 2**: To assess effectiveness of trained religious leaders’ engagement in maternal health education on antenatal care service utilization

**Objective 3**: To assess effectiveness of trained religious leaders’ engagement in maternal health education on skilled delivery service utilization

**Objective 4**: To develop and validate religious’ leaders maternal health education engagement scale: elicitation study on mothers’ perception on religious leaders based maternal health promotion then scale development

**Objective 5**: To explore perception of religious leaders in promoting maternal and child health in rural setting.

# Hypothesis

We hypothesize that trained religious leaders’ engagement in maternal health education will increase the proportion of antenatal care, skilled delivery service utilizations and knowledge of danger signs during pregnancy, labor and child birth and neonatal danger signs.

# Chapter four Methods and Materials

## 4.1 Study settings and period

This study will be conducted in the two rural districts (Lemo and Amaka) of Hadiya Zone in Southern Ethiopia. Hadiya zone is divided into 13 rural Districts and two administrative towns with total of 329 kebeles from which 303 of them rural and 26 of them were urban. Each district is divided into kebeles**;** the lowest administrative units in Ethiopia. Hadiya Zone hosts a total of 1,573,841 populations with a total area of 3542.66 Km^2^. Hadiya zone has an estimated 54455 pregnant mothers. Population of the Lemo District is 148,339. From this 73,390 are males and 74,950 are females. There are 34,563 women in reproductive age group from which 5133 is expected pregnancy. Total population of Amaka district is 80857, 4125 are females and 39622 is males. Expected pregnancy is 2797. Protestant, orthodox and Muslim are the three dominant religions in Hadiya zone. 75.35% of the population are [Protestants](https://en.wikipedia.org/wiki/P%27ent%27ay), 11.13% are [Muslim](https://en.wikipedia.org/wiki/Islam_in_Ethiopia), 8.45% are Ethiopian Orthodox Christians, and 4.31% are [Catholic](https://en.wikipedia.org/wiki/Roman_Catholicism_in_Ethiopia). This study will be conducted between October, 2022 annd May, 2023.

## 4.2 Study design

This study is a cluster randomized control trial to assess effectiveness of trained religious leaders’ engagement in maternal health education on maternal health behaviours. A total of five objectives will be answered from this study. Objectives 1, 2 and 3 are designed as a community-based cluster randomized control trial, which evaluates the effectiveness of trained religious leaders’ engagement in maternal health education on maternal health behaviours (ANC, SDS and knowledge of danger signs during pregnancy and child birth)**.** The fourth objective is designed as a cross-sectional study for the scale validation on engagement of religious leaders in promoting maternal health; the survey will assess how effectively the religious leaders deliver maternal education, to estimate dimensions of engagement. The fifth objective is qualitative study, designed as explorative grounded theory.

## 4.3 Source population

All pregnant women living in Misha district of Hadiya Zone, Southern Ethiopia.

## 4.4 Study population

Pregnant women in selected kebeles of district in Hadiya Zone, Southern Ethiopia

## 4.5 Inclusion and Exclusion Criteria

**Inclusion criteria**

- Pregnant women less than 20 weeks gestational age
- Living in selected kebele
- Pregnant women willing to participate in the study

**Exclusion Criteria**

- Pregnant women who are seriously ill and unable to communicate will be excluded from the study.

# 4.7 Study variables

## 4.7.1 Dependent variables

- ANC Utilization
- Skilled delivery service Utilization

## 4.7.1 Independent variables

**Socio-demographic and economic characteristics**

- Age
- Ethenicity
- Religion
- Income
- Marital status
- Educational status
- Occupation
- Family size
- Average distance from nearest HC

**Reproductive history of pregnant mothers**

- Parity
- Age at pregnancy
- Still birth
- Abortion
- Home delivery
- Institutional delivery
- Neonatal death

**Behavioural variables**

- Knowledge of the major obstetric danger signs
- Birth preparedness and complication readiness
- Attitude towards skilled delivery service utilization
- Perception of Pregnancy Risk
- Self-efficacy

All variables will be measured at the individual-level (i.e., for each pregnant mother) within study clusters.

## 4.8 Sample size

In accordance with the main objective of the larger study, the sample size estimate is designed to detect changes in the proportion of skilled delivery service utilization. The sample size is calculated using stata software based on the following assumptions. Tail (s): One; effect size (d): 0.15; **α** error probability = 0.05; power (1-**β** error probability) = 0.8, and allocation ratio (N1/N2) = 1. This gives a sample size of 155. Then, adding design effect, DE = 1+(m-1)ICC , where m=Average cluster size, m= 50, ICC=0.02 based on a published study ICC (Killip et al., 2004), DE=2, and allowing for a 10% loss to follow up, the total sample size is 682 (N1 = 341 and N2 = 341). N1 and N2 are sample sizes in the control and intervention group, respectively. Based on these assumptions, the estimated sample size is: 306 pregnant mothers per group to detect a 15-percentage point anticipated difference in the proportion of skilled delivery service utilization (an increase from 26% to 41%). This estimate is based on the baseline skilled delivery service utilization in the Ethiopia Demographic and Health Survey (26% from EDHS 2016, SNNP region report) in the control group (CSA, 2016).

**Sample size for qualitative study**

The sample size for the qualitative study will be determined based on the saturation of information. However, for a planned purpose, 12 in-depth interviews which is among religious leaders will be considered.

## 4.9 Randomization and Sampling procedure

First, 2 districts out of the 13 districts in Hadiya zone are selected purposely. Second, Non-adjacent 12 kebeles (clusters) are selected from two districts randomly. A pregnancy test will be done for the study women at the beginning of the surveillance. Then identified pregnant women with gestational age less than 20 weeks will be included in the study.

**For qualitative study:** A purposive sampling technique will be used to select the study participants for the in-depth interviews. Participants (religious leaders) will be selected based on their acceptance by their followers from each religion domination.

## Randomization

There are a total of 12 clusters for this cluster randomized trial. The researcher and kebele leaders, religious leaders and HEWs will conduct the randomization to produce comparable groups and eliminate the source of selection bias in the assignment of kebeles to the intervention and control groups. Simple randomization with a 1:1 allocation will be applied to assign clusters to either control or intervention groups. First, 12 nonadjacent clusters will be selected by a lottery method. Then, the 12 clusters were listed alphabetically. A list of random numbers will be generated in MS Excel 2010 and the generated values will be fixed by copying them as “values” next to the alphabetic list of the clusters. These are arranged in ascending order according to the generated random number. Finally, the first 6 clusters will be selected as intervention clusters and the last 6 as control clusters. A statistician that will be blinded to study groups and not participated in the trial will do the generation of the allocation sequence and the randomization of clusters (Figure below). The intervention group is assigned to receive training from religious leaders on maternal and neonatal health and the control group will be left to continue the current practices.

**Controlling contaminations and spill-over effects**

First, two districts are selected purposely from 13 districts in the Hadiya Zone. From two selected districts, 12 non-adjacent clusters are purposely and the clusters are allocated into either intervention or control group by simple randomization. The trained religious leaders will be assigned to provide intervention for pregnant mothers based on their residency of each cluster.

## 4.11 Trial design

This study is a parallel two-arm a cluster randomized controlled trial with 8 clusters. The trial arms are as follows: 1) religious leader training and 2) standard cares. The kebeles health posts are designated as clusters for the trial. Outcome assessments will be made using repeat cross-sectional surveys at baseline (prior to intervention roll-out) and at 4 months post intervention (i.e. the end line). A schematic for the trial design is displayed in Figure 2.

Screening

Two districts out of 13 districts of Hadiya Zone selected purposely

Non-adjacent 12 kebeles from two districts selected randomly

Randomization

Cluster randomization of 12 clusters to 2 study groups

Intervention groups: (6 clusters)

Control groups: (6 clusters)

Recruitment

-Recruit pregnant mothers with gestational age less than 20 wks. (n =341)

- Baseline data collection

base line data collection

**-**Recruit pregnant with mothers with gestational age less than 20 wks. (n =341)

-Baseline data collection

Intervention

Training for pregnant mothers by trained religious leaders

The usual care only

End line

End line data collection

End line data collection

Figure 2: Diagrammatic representation of the randomization of study cluster (kebeles)

## 4.12 Sampling techniques

Considering the number of kebeles that can meet the sample size, the study includes 12 kebeles. The kebeles (cluster at health post level) are randomly selected and assigned to the intervention and control groups). Distance among the kebeles is considered and some kebeles are left in between to serve as a corridor and avoid contamination from the religious leaders, and to minimize the likelihood of contact between religious leaders and thus the exchange of information among the groups.

## 4.13 Intervention

For 4 months, trial participants in the intervention group will receive behavioural change communication on maternal health, while those in the control group will receive usual care. In collaboration with HEWs, kebeles leaders and religious leaders, the researcher and field workers will arrange an appropriate training place in the intervention clusters to train sampled pregnant mothers on prenatal health behaviour. During the intervention period, Hadiyisa will be the language of communication (the local language).

**Recruiting and training of religious leaders**

Protestant, Orthodox, and Muslim are three religion domains in Hadiya Zone. First, the religious organizations in the study districts will be identified (table 1). Then the local religious leaders from each clustered kebele will be recruited based on religious educational status (educational status greater than or equal to diploma), acceptance by their followers and popularity, in collaboration with religious organization leaders, health extension workers, and kebele leaders. A total of 16 religious leaders will be recruited to be trainers of pregnant mothers. Then after the potential religious leaders are recruited, the two days training will be given for them. Recruited religious leaders are expected to give training on the topics (maternal health) for four sessions to promote healthy maternal behaviours for the members of their religion.

Table 1 Distribution of religious institutions in selected clusters (kebeles) in Lemo and Amaka districts

| s.no | Selected Clusters (kebeles) | Total population | Religious institutions | | | Total no. of Religious institutions |
| --- | --- | --- | --- | --- | --- | --- |
|  |  |  | Orthodox | protestant | Muslim |  |
| 1 | Gidasha | 5861 | 2 | 2 | 0 | 4 |
| 2 | Tinika | 5012 | 1 | 3 | 1 | 5 |
| 3 | Dima | 4428 | 1 | 3 | 1 | 5 |
| 4 | Geja | 3121 | 1 | 3 | 0 | 4 |
| 5 | Ushusha Bonka | 5457 | 1 | 2 | 1 | 4 |
| 6 | Borara | 3742 | 2 | 1 | 0 | 3 |
| 7 | Lambuda | 2567 | 1 | 2 | 1 | 4 |
| 8 | Lareba | 4378 | 1 | 3 | 1 | 5 |
| 9 | Bobicho | 2756 | 1 | 3 | 1 | 5 |
| 10 | Kalisha | 3492 | 0 | 3 | 1 | 4 |
| 11 | Shurmo | 3156 | 0 | 2 | 1 | 3 |
| 12 | Ambicho gode | 2867 | 1 | 3 | 1 | 5 |

The first training session will be conducted at the beginning of the intervention whereas the second session of similar content will be repeated after one month of the first intervention session and the third and fourth training sessions will be after one months of the second intervention session. After training sessions, every participant will receive a copy of the visual materials (posters) containing the key messages for promoting prenatal health behaviours. Direct, interactive and participatory learner and activity oriented instructional strategies will be delivered. Talks, group discussions, group work exercises, demonstrations, role plays, storytelling, simulation, case studies and problem-solving will be used to enhance knowledge, attitude, and behaviours on maternal and neonatal health (Table 2 shows over protocol of intervention).

Overall project coordination: by PI and co-PIs

Step 1:

Formative assessment: perception of mothers’ on engagement of religious leaders in promoting maternal health, and role of religious leaders in promoting maternal and child health: a qualitative exploration of religious leaders’ perspective

Baseline study: Intervention protocol development, data collection and analysis

Developing strategies and SBCC material flip chart, information card and poster and recruiting religious leaders

Randomization of the clusters in to intervention and control groups

Step 3: Pregnant mothers’ training by trained religious leaders

Training contents: Maternal health message contents, flip chart, poster, group education and group discussion

**Session 1:** Major causes of maternal and new-born Illness and death

**Session 2:** Focused Antenatal Care and danger signs during pregnancy

**Session 3:** BPCR and danger signs during labor and childbirth

**Session 4:** Components of PNC and Postnatal danger signs and neonatal danger signs

**Step 4: End line data collection, analysis and report writing**

Step 2: Religious leaders’ training by project PI

Training contents: Maternal health message contents, flip chart, poster and group education

Trained religious leaders led SBCC to improve maternal health behavior

Figure 3. Presents the intervention processes and package of trained religious leaders led SBCC.

| Session | **Content of the message** | **Dose** | **Strategy of delivery** | **Frequency** | **Compliance parameters** | **Responsible person** |
| --- | --- | --- | --- | --- | --- | --- |
| Session 1 | - Major causes of maternal and new-born Illness and death | 1:00 hour | • Brain storming • Group education/ Group work • Case study/ case scenario • Question and Answer (Q&A) • Agree/ Disagree exercises • Group Presentation | Once at their 2^nd^ trimester period | -% pregnant mothers participated | Researcher and trained religious leaders |
| Session 2 | - Focused Antenatal Care - About danger signs during pregnancy | 1:00 hours | Brain storming • Group education/ Group work • Case study/ case scenario • Question and Answer (Q&A) • Agree/ Disagree exercises • Group Presentation | Once at their 2nd trimester period | -% pregnant mothers participated | Researcher and trained religious leaders |
| Session 3 | - Birth preparedness and complication readiness - Danger signs, labor and childbirth, - Components of postnatal care and Postnatal danger signs and neonatal danger signs | 1:00 hour | Brain storming • Group education / Group work • Case study/ case scenario • Question and Answer (Q&A) • Agree/ Disagree exercises • Group Presentation | Once at their 3^rd^ trimester period | -% pregnant mothers participated | Researcher and trained religious leaders |
| Session 4 |  | 1:00 hour | Brain storming • Group education / Group work • Case study/ case scenario • Question and Answer (Q&A) • Agree/ Disagree exercises • Group Presentation | Once at their 3^rd^ trimester period | -% pregnant mothers participated | Researcher and trained religious leaders |
|  | **Providing specific take-home print materials**   - ANC practices - SDS practice | -Poster  -Information card  -Flip chart | - | Delivered during in each training session | -% of pregnant mothers received print materials | Researcher and trained religious leaders |
|  | **End line data collection** | - | - | at 4^th^ month of intervention | % pregnant mother interviewed | Researcher, supervisors data collectors. |

Table 2 protocol interevention

**The focuses of training (intervention key messages) on:**

**Antenatal care:** (taking TT injection, taking Calcium Consumed 100+ IFA tablets , eating more food during pregnancy, eating Green vegetables , eating Fruits during pregnancy , drinking milk during pregnancy , eating pulses and beans during pregnancy , taking nap/rest, regularly during pregnancy, Husband accompanying for ANC (regularly /mostly), Human immunodeficiency virus (**HIV)**  **and syphilis** test, tobacco use and substance use. **Benefits of early and exclusive breastfeeding (**Provides the best nutrition for the new-born Is easily digested and efficiently used by the baby’s body, Protects against infection and other illnesses, Offers some protection against allergies, Is cost-effective and affordable, Promotes mother-baby bonding. **Unhealthy beliefs and practices about feeding new-borns**, **Hygiene during pregnancy**, **Breastfeeding and contraception, Benefits of birth spacing: (**Maternal mortality, fetal death (miscarriage or stillbirth), neonatal mortality, Anaemia in the mother during subsequent pregnancies, Postpartum inflammation of the endometrium lining the uterus, Premature rupture of the amniotic membranes surrounding the fetus, Premature birth, Intrauterine growth retardation and a low birth-weight baby and Malnutrition of new-borns and infants due to insufficient breast-milk**).**

**Prenatal Danger signs: (**Severe headache, Blurred vision, Fatal movement absent, High blood pressure, Edema of the face/swelling, Oedema of the hands/leg swelling, Convulsions, Excessive vaginal bleeding, Severe lower abdominal pain and Leaking fluid (meconium stained).

**Labor/delivery** **Danger signs: (**Excessive vaginal bleeding, Foul-smelling discharge, High fever, Baby’s hand or feet coming out first, Baby is in abnormal position, Prolong labor (.12 hours), Retained placenta, Rupture uterus, Cord prolapse, Cord around neck and Convulsion).

**Postpartum Danger signs:** (Excessive vaginal bleeding**,** Foul-smelling discharge**,** High fever**,** Inverted nipples**,** Tetanus**,** Retained placenta**,** severe abdominal pain**,** Convulsions **and** Engorged breasts/swelling of breasts)

**Neonatal Danger signs: (**Poor feeding or unable to suck**,** Diarrheal**,** Redness around the cord**,** Red eye/discharging eyes**,** Difficult breathing**,** Yellow coloration of the skin/jaundice**,** Hypothermia/shivering**,** Blisters on skin/Skin lesion**,** Baby doesn’t cry**,** Fever**,** Unconscious**,** Fast breathing**,** Chest in drawing**,** Doesn’t pass urine**,** Doesn’t pass stool and Convulsions.

**Consulting doctor or others health professionals:** if they have danger signs (during pregnancy, labor and childbirth, 42 days after delivery, and neonatal danger signs).

**Birth preparedness and complication readiness:** (plan for where to give birth, plan for a skilled birth attendant, plan to save money, plan for transportation and identification of compatible blood donors in case of emergency).

**Delivery at a health facility with a skilled provider** and **postnatal care:** (Women discuss FP method with husband Women adopted FP method after delivery Mother fed colostrum to the baby).

**Group training of pregnant mothers by Religious leaders**

Each trained religious leader will be assigned to deliver group training for pregnant mothers residing in cluster. The trained religious leaders will deliver a total of four group training sessions for the pregnant mothers they are assigned with the same training procedures provided by the researcher during training of religious leaders. Religious leaders will be trained to conduct culturally appropriate training sessions with pregnant mothers using facilitator manual and posters prepared in the local language.

**Control group (standard care)**

The control group in this study will be on the existing routine maternal service without a provision of religious leaders’ training intervention. In this arm, there will be no intervention by the researchers, rather baseline and end line data will be collected.

## 4.14 Cluster and individual selection

Health posts are eligible for trial. All 40 health post units in the selected districts are eligible and 12 health posts are randomly selected for the trial using a computer random number generator. Pregnant mothers less than or equal to 20 gestational age are eligible to participate in the trial if they are living in the villages within the selected health post village areas. Baseline surveys will be commenced in May 2022 and end line surveys are scheduled to begin after four months of intervention.

The pregnancy surveillance for this study will be conducted among currently married women of reproductive age (15–49 years) permanently living in the selected districts. A pregnancy test will be done for the study women at the beginning of the surveillance. Identifying pregnant women will be a two-step process. First, all women will be screened using pregnancy screening questions adapted from the Kenya family planning enrolment questionnaire as published in the Lancet (Stanback J et al, 199) (Table 2). If the answer to at least one of the six pregnancy screening questions is ‘yes’, pregnancy will be ruled out. Women who respond in a way that suggested the possibility of being pregnant is asked to provide a urine sample for a pregnancy test, which will be done using a dipstick in the respondent’s home. Once pregnancy is detected, the women will be recruited for the trial study.

Table 2 Pregnancy screening questions adapted from Kenya family planning screening questions

| S/No | Items | Yes or No |
| --- | --- | --- |
| 1 | Have you given birth in the past 4 weeks? |  |
| 2 | Are you less than 6 months post-partum or fully breastfeeding and free from menstrual bleeding since you had your child? |  |
| 3 | Did your last menstrual period start with in the past 7 days? |  |
| 4 | 4 Have you had a miscarriage or an abortion in the past 7 days? |  |
| 5 | Have you abstained from sexual intercourse since your last menses? |  |
| 6 | Have you been using a reliable contraceptive (pills, injectable, and Norplant) method consistently and correctly? |  |

## 4.15 Intervention assignment and masking

Randomisation is stratified by kebeles, with 12 clusters in the district allocated to either control or intervention in a public randomisation meeting. From each clusters (kebele), local stakeholders (HEWs, kebele leaders and religious organization leaders) are invited, a number is allocated to each cluster, the numbers are written on small plastic balls, and placed the balls in a dark bag. Each participant is asked to draw one ball from the bag and read out the cluster numbers out until all balls are picked. The cluster numbers are written on a sheet of paper in order of selection. Participants then place 12 pieces of paper numbered 1 to 12—each corresponding to a unique allocation sequence generated by an independent statistician—in the dark bag, and asked a participant to select a paper and read out the number. The corresponding sequence is then used to publicly allocate each cluster to one of two groups. Because of the nature of the intervention being tested, the intervention team could not be masked to allocation. The data collection team will be masked to allocation, both at the cluster and at the individual level. Blinding is done to control for bias as follows. The researcher does not inform the study interventions and hypothesis for the participants who participate during the randomization of the kebeles into study groups.

**Participant timeline**

Clusters will be enrolled and randomized to trial arms in June 1, 2022. Baseline recruitment and interviewing of women within study clusters began in June 5, 2022 and will be completed after four months of intervention.

**Intervention fidelity**

Fidelity of the intervention will be maintained based on the National Institutes of Health Behavioural Change Consortium developed best practice recommendations (Bellg et al., 2004). The intervention design has conceptual framework. Non adjacent clusters will be selected to prevent information contamination. Equal numbers of clusters will be taken for the intervention and control groups from each district to balance variations. The intervention process will be pretested before the implementation of the trial. Each pregnant mother will receive equal numbers and frequencies of training, and the lengths of contacts within an intervention group will be similar to make the process standardized. Group education training will be given in a group using a training manual, role-playing, and mock counselling practice. The competency of recruited religious leaders will be monitored by pre-post training test. The test focuses on knowledge and attitudes towards intervention key messages. If the post-training test score of the trained religious leaders is below acceptable range, retraining will be given on identified gaps of intervention messages. Training sessions will be randomly selected for process evaluation and all selected sessions will be evaluated by one process evaluator. The process observer rated the educator using a ‘yes/no’ rating system on items such as using a training guide, provision of the whole content, duration and frequency of training, preparedness, accuracy, and ability to properly respond to questions. Intervention receipt will be assessed using checklists on knowledge of the pregnant mothers on maternal health behaviour through interviewing about their understanding of the core contents of the intervention. Intervention enactment will be assessed on utilizations of antenatal care and skilled delivery.

Routes of **persuasive message**

*Central route:* The ability of a person to comprehend information depends on the quality of the information which can be described into two elements: information completeness and information accuracy(Chang et al., 2020, Filieri and McLeay, 2014). This study adopted both elements as central route factors. Information completeness is referred to when there is sufficient depth and breadth of information in communication (Chang et al., 2020). Information accuracy for this study is defined as the degree to which information is correct, accurate, and unambiguous (Chang et al., 2020, Lee et al., 2002). Accurate information needs to show interrelation and consistency between provided data and reality (Thoroddsen et al., 2013). In the context of maternal health education through religious leaders, information accuracy requires all the discussed information to be accurate and consistent. Scholars argue that to increase the effectiveness and accuracy of information, attention needs to be given to the source of communication.

***Peripheral Route:*** According to the elaboration of likelihood model, besides argument quality representing the central route in influencing attitude, the peripheral route is best explained by other “cues” that affect the individual attitude through bypassing the argument processing (Petty and Cacioppo, 1986). Scholars argue that the cue is operative when the individual is being “unmotivated by the subjects or is unable to process the issue-relevant arguments”, abundantly found in the central route (Petty and Cacioppo, 1986). Therefore, the individual looks for simpler cues, such as source credibility, aesthetics, and popularity (Chang et al., 2020). In this study, these peripheral routes of persuasion of maternal health education messages will be considered during the recruitment of the religious leaders.

**4.16 Measurements and indicators**

Trained interviewers will conduct face-to-face interviews using structured questionnaires. Questionnaires contain sections on socio-demographics, Reproductive history of respondent, maternal health service utilization, birth preparedness and complication readiness, knowledge of danger signs during pregnancy, labor and child birth, new-born and postpartum period, perception about pregnancy, childbirth, and the period immediately after childbirth, attitude towards safe delivery utilization and religious leaders’ engagement scale measurement tool. Antenatal care service utilization will be measured based on “at least one ANC attendance” (Women who have attended at least one ANC check-up during their current pregnancy) and “four or more ANC attendance” (Women who attended four or more ANC visits) during their current pregnancy as reported by the participant. Skilled delivery service utilization will be measured based on women who gave birth in health center and hospital by assistance of health professionals that have midwifery skills including Midwife nurse, Nurse, Health Officers and Doctors *as* reported by the participant.

Knowledge of danger signs during pregnancy has 14 items, Knowledge of danger signs of labor and childbirth has 9 items and knowledge of neonatal danger signs has 11 items. All Knowledge score will be computed by adding the total number of correct spontaneous responses to maximum number of items with a minimum score of 0 and maximum of number of items (0 when a mother mentioned none of the key danger signs and maximum number when the mother mentioned all the danger signs). Spontaneous response is respondents’ naming of danger signs without giving option of the respective signs. Accordingly, two categories will be developed for knowledge of danger signs (Good and poor categories). Women who mention at least three neonatal danger signs will be considered to have good knowledge whereas those who mentioned less than three of the danger signs will be labelled to have poor knowledge as stated by several studies. Questionnaire on BPCR is adopted and modified from monitoring BPCR tools for maternal and new-born health (Maternal, 2004). Attitude towards skilled delivery service utilization has 12 items that will be measured by using Likert scale where respondents are supposed to strongly agree, agree, neutral, disagree and strongly disagree. The overall attitude score will computed by summing up the items after performing the reverse scoring for negatively worded sentences. A higher composite score indicated a more favourable attitude.

*Perception of Pregnancy Risk Questionnaire (PPRQ****)*** was developed by Heaman and Gopton (Heaman and Gupton, 2009). It is a self-report questionnaire consisting of 9 visual analogue scales designed to measure a pregnant woman’s perception of her pregnancy risks. This questionnaire consists of two subscales that include four questions about the risk to self (mother), (for example “is a risk of having a cesarean section”); and five questions about risk to the baby, (for example “is a risk of baby having a birth defect”). “Respondents will be asked to put a vertical mark through the line to indicate their assessment of risk for each Item,” (yielding a score ranging from 0-100). A total PPRQ score will be obtained by adding the score for each of the 9 items, and then dividing by 9, to obtain a score out of 100. Higher scores will indicate higher levels of perceived risk.

## 4.17 Data collection and management

The principal investigator will train the data collectors and supervisors for two
consecutive days on instruction; in the quantitative method, study guides, role-plays
(demonstration), informed consent, how to approach participants, how to collect data using
mobile health, ethical procedure and general information and the objective of
the study. The data will be collected by using a mobile-based application called open data kit
(ODK). In the initial step, a pre-intervention survey will be conducted after the randomization and allocation. And there will be two times data collection including the pre-intervention and post-intervention data. The data will be collected by 4 months interval to have a good longitudinal data. The type of data will be panel data with repeated cross-sectional. Experienced data collectors with health background and Hadiyisa speaking will be recruited to collect the data. Besides, a master holder with health profession will be employed to supervise the whole data collection process.

**Baseline Data Collection**

Six clinical nurses for data collection and two supervisors with master holder in public health will be used. Intensive training will be given for data collectors and supervisors on the item. The data collectors and the supervisors will be assigned to a different cluster of a given district. Interviews are expected to last approximately 1 h and will be conducted in a quiet, private space at the homes of the women. If selected women are absent from their homes, interviewers will visit households up to three different days/times to attempt to interview them before the woman will be replaced with another randomly selected woman. The data will be collected for a total of 15 days.

**End line Data collection**

The same procedure with the baseline data will be repeated to collect the end line data after one month of delivery. Six data collectors with health background will be used to collect the data. Two supervisors will be assigned to check for the daily activity, consistency, and completeness of the questionnaire and to give appropriate support during the data collection process. The data collectors and the supervisors will be assigned to a different cluster of a given district.

Qualitative Data collection

Focus group discussions and in-depth interviews among religious leaders and pregnant mothers will be conducted. Trained graduate research assistants will conduct Focus group discussions and in-depth interviews. Data will be collected by recording interviews by audiotape. Brief field notes will be taken during interviews. Field notes will be carefully taken during data collection and analysis. These notes will collect observations and assumptions about what will be heard or observed and personal narratives about what will be felt by the researcher during an interview.

## 4.18 Data Quality Control

To assure the quality of the data, data collectors and supervisors will be trained and a regular supervision and follow-up will be made by supervisors and the principal investigator. In addition, a regular check-up for completeness and consistency of the data will be made on daily basis. The questionnaire is translated into Amharic language and back-translated into English by translators who are blind to the original questionnaire. To make the questionnaire standard, reliability and validity test will be done for scale variables. Pilot test and Pre-test of the tool will be carried out in the area which has similar characteristics with the study population in Anlemo district will be done before two weeks of the actual data collection to ensure clarity, wordings, logical sequence and skip patterns of the questions. The pre-tested sample will be not included in the study and modification will be made.

For the qualitative study: Silent and comfortable places and convenient times will be selected and arranged to conduct, in-depth interview with religious leaders so as to allow maximum concentration. Study participants will also be made to give a true answer through explaining the purpose and importance of the study and assuring the confidentiality of data they are going to provide. To enhance inter-coder reliability, the coders each will independently apply the code book to a selected and rich transcript and review any differences in their coding, which will be discussed and resolved.

**Operational definition**

**Skilled delivery service utilization**

Skilled delivery service utilization will be measured based on women who gave birth in health center and hospital by assistance of health professionals that have midwifery skills including Midwife nurse, Nurse, Health Officers and Doctors *as* reported by the participant.

**Antenatal care service utilization**

Antenatal care service utilization will be measured based on “at least one ANC attendance” (Women who have attended at least one ANC check-up during their current pregnancy) and “four or more ANC attendance” (Women who attended four or more ANC visits) during their current pregnancy as reported by the participant.

**Birth preparedness and complication readiness**

Birth preparedness: A woman will be classified as “well birth prepared” in the most recent pregnancy if she has accomplished three of the following practices: identified skilled health professional, saved money, identified transport or had delivery kit/materials. A woman who makes arrangements for birth in less than three of the four ways will be classified as “not well birth prepared”.

**Knowledge of obstetric danger signs**

Women who mention at least three neonatal danger signs will be considered to have good knowledge whereas those who mentioned less than three of the danger signs will be labelled to have poor knowledge as stated by several studies.

**Perception of pregnancy risk**

Risk perception is operationalized using the adapted Perception of Pregnancy Risk Questionnaire (PPRQ) developed by Heaman & Gupton. Higher scores will indicate higher levels of perceived risk.

**Attitude towards skilled delivery service utilization**

Attitude will be measured by using five point Likert scale. Positive attitude will be scored by participants who respond above the mean of the attitude assessment questions and if below the mean they were categorized as having negative attitude

## 4.19 Statistical analysis

Data of baseline and end-line surveys will be combined and analyzed using Stata 14.1. Analyses will be based on the intention-to-treat principle and compared differences of the outcomes between the intervention and control at both individual and cluster levels. First, Univariate analyses will be performed to explore the characteristics of respondents. We then will use logistic regression with random effects to estimate the effect of the intervention on prenatal behaviour, adjusting for clustering. Generalized estimated equations (GEE) regression analyses adjusted for clustering will be used to test the effect of the intervention on promoting maternal health behaviour. We will repeat analyses for the primary and secondary outcomes adjusted for baseline differences by fitting an interaction term between study period (baseline vs intervention) and allocation in each model. Mixed-effects multilevel logistic regression model will be used to identify determinants of birth preparedness and complication readiness.

**For qualitative study:** The interviews will be recorded and transcribed verbatim. The transcripts will be thematically analyzed using the qualitative software program Atlas.ti 6.0. Two analysts will independently code the transcripts and subsequently review, discuss, and refine the coding schemes until consensus will be reached. Emerging concepts will be assessed using the constant comparative method from grounded theory. This means that when this concept will be identified, previously analyzed interviews will be reviewed in order to check if their content fitted into this concept. A final version of the code book will be then developed leading to identification of themes, and sub-themes. Coded transcripts will be further analyzed and summarized in narratives for each theme and sub-theme. Study findings will be presented, discussed, and validated in a stakeholder meetings conducted in regional and district health offices.

| S.no | Research objectives | Study populations | Study design | Data source | Major outcome variables | Analytic model |
| --- | --- | --- | --- | --- | --- | --- |
| 1 | To assess effectiveness of trained religious leaders’ engagement in maternal health education on knowledge of danger signs during pregnancy and child birth | Pregnant mothers | Cluster randomized control trial | Household survey | knowledge of danger signs during pregnancy and child birth | Generalized estimated equations (GEE) regression analyses |
| 2 | To assess effectiveness of trained religious leaders’ engagement in maternal health education on antenatal care service utilization | Pregnant mothers | Cluster randomized control trial | Household survey | antenatal care service utilization | Generalized estimated equations (GEE) regression analyses |
| 3 | To assess effectiveness of trained religious leaders’ engagement in maternal health education on skilled delivery service utilization | Pregnant mothers | Cluster randomized control trial | Household survey | skilled delivery service utilization | Generalized estimated equations (GEE) regression analyses |
| 4 | To develop and validate religious’ leaders maternal health education engagement scale: elicitation study on mothers’ perception on religious leaders based maternal health education then scale development | Pregnant mothers | Cross-sectional study | Household survey | Religious leaders engagement in maternal health education | exploratory factor analysis |
| 5 | To explore role of religious leaders in promoting maternal and child health: a religious leaders’ view in rural setting of Hadiya Zone, Southern Ethiopia.  **Research questions:**   1. Do religious leaders address the topic of maternal health in their contacts with members of their congregations? If so, when and how do they address the topic? 2. To what extent religious leaders willing to enter into a dialogue with authorities on the topic of maternal health? | Religious leaders | Grounded theory | In-depth interviews and FGDs among religious leaders | NA | Thematic analysis |

## Table 4: Summary of study design

## 4.20 Dissemination plan

At the end of the interventional study, a study finding will be compiled. The finding will be disseminated to key stakeholders involving in the study. Scale upping and follow up will be made to ensure the sustainability of the intervention to promote prenatal health behaviour. The principal investigator will take the leading role of the finding dissemination activities. Short communications (poster, policy brief, abstract presentations) will be carried out. Efforts will be made to publish the finding in international peer reviewed and reputed journals.

## 4.21 Ethical consideration

All procedures involving the research will be approved by Jimma University institute of Health ethical review board. Permission to undertake the study will be obtained from the regional, Zonal and district administration and health offices of the study area. After the identification of eligible pregnant mothers, the nature and purpose of the study will be explained along with their right to refuse. Written and informed consent will be obtained from all study participants. The right of the participant to withdraw from the study at any time will be respected. The data will not be accessed by a third person, except investigators, and will be kept confidential. The study will be registered at clinicaltrials.gov.

| S. No | Activities | 15/03/21 -20/03/21 | 01/04/21 – 20/08/2021 | 02/09/21 – 02/10/21 | 09/11/201 | 23-30/12/21 | 1-20/01/22 | 20/02/23 – 23/03/23 | 20/05/23 – 25/09/23 | 22/10/23- 23/11/23 | 30/12/23 – 1/03/203 | Responsible body | Remark |
| --- | --- | --- | --- | --- | --- | --- | --- | --- | --- | --- | --- | --- | --- |
|  | Discussing on title and fixing with my promoters |  |  |  |  |  |  |  |  |  |  | Abinet A. & Promoters |  |
|  | Preparation of dissertation proposal |  |  |  |  |  |  |  |  |  |  | Abinet A. |  |
|  | Preparation of data collection tools |  |  |  |  |  |  |  |  |  |  | Abinet A. |  |
|  | Sending proposal to promoters |  |  |  |  |  |  |  |  |  |  | Abinet A. |  |
|  | Accepting promoters’ comments and suggestions, modifying proposal accordingly |  |  |  |  |  |  |  |  |  |  | Abinet A. & Promoters |  |
|  | Submission of proposal to department and Doctoral School |  |  |  |  |  |  |  |  |  |  | Abinet A. & Promoters |  |
|  | Proposal defence |  |  |  |  |  |  |  |  |  |  | Abinet A. |  |
|  | Receiving ethical clearance |  |  |  |  |  |  |  |  |  |  | Abinet A. |  |
|  | Base line data collection |  |  |  |  |  |  |  |  |  |  | Abinet A., data collectors and supervisors |  |
| 11 | Intervention for study subjects |  |  |  |  |  |  |  |  |  |  | Abinet A. and trained religious leaders |  |
| 12. | End line data collection |  |  |  |  |  |  |  |  |  |  | Abinet A., data collectors and supervisors |  |
| 13 | Analysis of data |  |  |  |  |  |  |  |  |  |  | Abinet A. & Promoters |  |
| 14 | Manuscript preparation |  |  |  |  |  |  |  |  |  |  | Abinet A. & Promoters gust & student |  |
| 16 | Manuscript submission to Journals |  |  |  |  |  |  |  |  |  |  | Abinet A. & Promoters |  |
| 17 | PhD thesis defence |  |  |  |  |  |  |  |  |  |  | Abinet A. & Promoters |  |

Table 5: Tentative work pla

Table 3: Budget summary

| **S/no** | **Item** | **Total requested money in ETB** | **Remark** |
| --- | --- | --- | --- |
| 1 | Personnel fee | 163,314 | Per-diem for religious leaders giving training to the period of intervention, data collectors; supervisors; and baseline to end-line study |
| 2 | Refreshment | 14,765.00 | To be used during training of religious leaders |
| 3 | Health Learning Materials | 62,891.00 | For the intervention of behavioural change leaflet, brochures, poster and banner |
| 4 | Communications | 1,750.00 | Mobile cards to communicate with couples and HEWs |
| 5 | Stationery | 13,280.00 | Training, data collection, pregnant mothers, religious leaders and HEWs |
| **Grand total** | | **256,000 ETB** |  |

# References

ABOSSE, Z., WOLDIE, M. & OLOLO, S. 2010. Factors influencing antenatal care service utilization in hadiya zone. *Ethiopian Journal of Health Sciences,* 20.

ABOUZAHR, C. & WARDLAW, T. 2003. Antenatal care in developing countries: promises, achievements and missed opportunities-an analysis of trends, levels and differentials, 1990-2001. *Antenatal care in developing countries: promises, achievements and missed opportunities-an analysis of trends, levels and differentials, 1990-2001.*

ADEDINI, S. A., BABALOLA, S., IBEAWUCHI, C., OMOTOSO, O., AKIODE, A. & ODEKU, M. 2018. Role of religious leaders in promoting contraceptive use in Nigeria: evidence from the Nigerian urban reproductive health initiative. *Global Health: Science and Practice,* 6**,** 500-514.

AHMED, S., CREANGA, A. A., GILLESPIE, D. G. & TSUI, A. O. 2010. Economic status, education and empowerment: implications for maternal health service utilization in developing countries. *PloS one,* 5**,** e11190.

ALEMI KEBEDE, K. H. & TEKLEHAYMANOT, A. N. 2016. Factors associated with institutional delivery service utilization in Ethiopia. *International journal of women's health,* 8**,** 463.

AMENTIE, M., ABERA, M. & ABDULAHI, M. 2015. Utilization of family planning services and influencing factors among women of child bearing age in Assosa district, Benishangul Gumuz regional state, West Ethiopia. *Sci J Clin Med,* 4**,** 52.

AMOUZOU, A., HAZEL, E., VAZ, L., YAYA, S. & MORAN, A. 2020. Discordance in postnatal care between mothers and newborns: Measurement artifact or missed opportunity? *Journal of global health,* 10.

AMZAT, J. 2020. Faith effect and voice on early marriage in a Nigerian state. *SAGE Open,* 10**,** 2158244020919513.

ANSHEL, M. H. & SMITH, M. 2014. The role of religious leaders in promoting healthy habits in religious institutions. *Journal of religion and health,* 53**,** 1046-1059.

BAROT, S. 2013. A common cause: faith-based organizations and promoting access to family planning in the developing world. *Guttmacher Policy Review,* 16**,** 18-23.

BENOVA, L., OWOLABI, O., RADOVICH, E., WONG, K. L., MACLEOD, D., LANGLOIS, E. V. & CAMPBELL, O. M. 2019. Provision of postpartum care to women giving birth in health facilities in sub-Saharan Africa: a cross-sectional study using demographic and health survey data from 33 countries. *PLoS medicine,* 16**,** e1002943.

BERHAN, Y. & BERHAN, A. 2014. Review of maternal mortality in Ethiopia: a story of the past 30 years. *Ethiopian journal of health sciences,* 24**,** 3-14.

BLACK, R. E., COUSENS, S., JOHNSON, H. L., LAWN, J. E., RUDAN, I., BASSANI, D. G., JHA, P., CAMPBELL, H., WALKER, C. F. & CIBULSKIS, R. 2010. Global, regional, and national causes of child mortality in 2008: a systematic analysis. *The lancet,* 375**,** 1969-1987.

BOHREN, M. A., HUNTER, E. C., MUNTHE-KAAS, H. M., SOUZA, J. P., VOGEL, J. P. & GÜLMEZOGLU, A. M. 2014. Facilitators and barriers to facility-based delivery in low-and middle-income countries: a qualitative evidence synthesis. *Reproductive health,* 11**,** 1-17.

BORMET, M., KISHOYIAN, J., SIAME, Y., NGALANDE, N., ERB, K., PARKER, K., HUBER, D. & HARDEE, K. 2021. Faith-Based Advocacy for Family Planning Works: Evidence From Kenya and Zambia. *Global Health: Science and Practice*.

CAMPBELL, M. K., HUDSON, M. A., RESNICOW, K., BLAKENEY, N., PAXTON, A. & BASKIN, M. 2007. Church-based health promotion interventions: evidence and lessons learned. *Annu. Rev. Public Health,* 28**,** 213-234.

CAMPBELL, O. M., GRAHAM, W. J. & GROUP, L. M. S. S. S. 2006. Strategies for reducing maternal mortality: getting on with what works. *The lancet,* 368**,** 1284-1299.

CANADA, P.-U. A. P. I. 2020. Engaging Religious Leaders in Reducing Maternal and Child Mortality, and Gender Equality Field Experiences from the SHOW Project in Sokoto State, Nigeria. Washington, DC and Toronto, Ontario: Promundo-US and

Plan International Canada.

CHAKA, E. E., ABDURAHMAN, A. A., NEDJAT, S. & MAJDZADEH, R. 2019. Utilization and determinants of postnatal care services in Ethiopia: a systematic review and meta-analysis. *Ethiopian journal of health sciences,* 29.

CHILONGOZI, M. N. 2017. The role of the church with regards to maternal health: a case study of the Church of Central Africa Presbyterian, Synod of Livingstonia.

CLARK, S. L. Strategies for reducing maternal mortality. Seminars in perinatology, 2012. Elsevier, 42-47.

CSA 2001. Central Statistical Authority [Ethiopia] and ORC Macro. 2001. Ethiopia Demographic and Health Survey 2000. Addis Ababa, Ethiopia and Calverton, Maryland, USA: Central Statistical Authority and ORC Macro.

CSA 2012. Ethiopia Demographic and Health Survey 2011. Addis Ababa, Ethiopia and Calverton, Maryland, USA: Central Statistical Agency and ICF International.

CSA 2016. Central Statistical Agency (CSA) [Ethiopia] and ICF. 2016. Ethiopia Demographic and Health Survey 2016. Addis Ababa, Ethiopia, and Rockville, Maryland, USA: CSA and ICF.

DAMTEW, Z. A., KARIM, A. M., CHEKAGN, C. T., ZEMICHAEL, N. F., YIHUN, B., WILLEY, B. A. & BETEMARIAM, W. 2018. Correlates of the Women’s Development Army strategy implementation strength with household reproductive, maternal, newborn and child healthcare practices: a cross-sectional study in four regions of Ethiopia. *BMC pregnancy and childbirth,* 18**,** 5-17.

DESTA, F. A., SHIFA, G. T., DAGOYE, D. W., CARR, C., VAN ROOSMALEN, J., STEKELENBURG, J., NEDI, A. B., KOLS, A. & KIM, Y. M. 2017. Identifying gaps in the practices of rural health extension workers in Ethiopia: a task analysis study. *BMC health services research,* 17**,** 1-9.

DIALOGUE, W. F. D. 2014. A Report by the World Faiths Development Dialogue with Support from the United Nations Foundation, Universal Access Project.

DIAMOND-SMITH, N. & SUDHINARASET, M. 2015. Drivers of facility deliveries in Africa and Asia: regional analyses using the demographic and health surveys. *Reproductive health,* 12**,** 1-14.

DUTAMO, Z., ASSEFA, N. & EGATA, G. 2015. Maternal health care use among married women in Hossaina, Ethiopia. *BMC Health Services Research,* 15**,** 1-9.

EPHI 2014. Ethiopian Public Health Institute. Ethiopia Service Provision Assessment Plus Survey. Addis Ababa; 2014.

EPHI 2019. Ethiopian Public Health Institute [Ethiopia] and ICF. 2019. Ethiopia Mini Demographic and Health Survey 2019: Key Indicators. Rockville, Maryland, USA: EPHI and ICF.

ESHETE, T., LEGESSE, M. & AYANA, M. 2019. Utilization of institutional delivery and associated factors among mothers in rural community of Pawe Woreda northwest Ethiopia, 2018. *BMC research notes,* 12**,** 1-6.

FETENE, N., CANAVAN, M. E., MEGENTTA, A., LINNANDER, E., TAN, A. X., NADEW, K. & BRADLEY, E. H. 2019. District-level health management and health system performance. *PloS one,* 14**,** e0210624.

FIKRE, A. A. & DEMISSIE, M. 2012. Prevalence of institutional delivery and associated factors in Dodota Woreda (district), Oromia regional state, Ethiopia. *Reproductive health,* 9**,** 1-6.

FMOH 2015. FMOH. HSTP Health Sector Transformation Plan. 2015.

FOULKES, A. A. A. E. 2014. ENGAGING FAITH LEADERS IN FAMILY PLANNING: A Review of the Literature plus Resources, World Vision US.

GEORGETOWN UNIVERSITY, I. F. R. H. I. 2011. Faith-Based Organizations as Partners in Family Planning: Working Together to Improve Family Well-being.

GIRMAYE, M. & BERHAN, Y. 2016. Skilled antenatal care service utilization and its association with the characteristics of women’s health development team in Yeky District, south-west Ethiopia: A multilevel analysis. *Ethiopian Journal of Health Sciences,* 26**,** 369-380.

GRABENSTEIN, J. 2014. LOCAL FAITH COMMUNITIES AND IMMUNIZATION FOR COMMUNITY AND HEALTH SYSTEMS STRENGTHENING.

GREDE, N., DE PEE, S. & BLOEM, M. 2014. Economic and social factors are some of the most common barriers preventing women from accessing maternal and newborn child health (MNCH) and prevention of mother-to-child transmission (PMTCT) services: a literature review. *AIDS and Behavior,* 18**,** 516-530.

GULIANI, H., SEPEHRI, A. & SERIEUX, J. 2014. Determinants of prenatal care use: evidence from 32 low-income countries across Asia, Sub-Saharan Africa and Latin America. *Health policy and planning,* 29**,** 589-602.

HARVEY, S. A., BLANDÓN, Y. C. W., MCCAW-BINNS, A., SANDINO, I., URBINA, L., RODRÍGUEZ, C., GÓMEZ, I., AYABACA, P. & DJIBRINA, S. 2007. Are skilled birth attendants really skilled? A measurement method, some disturbing results and a potential way forward. *Bulletin of the World Health Organization,* 85**,** 783-790.

HEMBLING, J., MCEWAN, E., ALI, M., PASSANITI, A., ARYEE, P. A. & SAAKA, M. 2017. Mobilising faith-based and lay leaders to address antenatal care outcomes in northern Ghana. *Development in Practice,* 27**,** 634-645.

HOLT, C. L., LEWELLYN, L. A. & RATHWEG, M. J. 2005. Exploring religion-health mediators among African American parishioners. *Journal of Health Psychology,* 10**,** 511-527.

JACKSON, R. & HAILEMARIAM, A. 2016. The role of health extension workers in linking pregnant women with health facilities for delivery in rural and pastoralist areas of Ethiopia. *Ethiopian journal of health sciences,* 26**,** 471-478.

JIRA, C. & BELACHEW, T. 2005. Determinants of antenatal care utilization in Jimma Town, SouthWest Ethiopia. *Ethiopian journal of health Sciences,* 15.

JOHNSON, L. & WALL, B. M. 2014. Women, religion, and maternal health care in Ghana, 1945-2000. *Family & community health,* 37**,** 223-230.

KILLIP, S., MAHFOUD, Z. & PEARCE, K. 2004. What is an intracluster correlation coefficient? Crucial concepts for primary care researchers. *The Annals of Family Medicine,* 2**,** 204-208.

KOBLINSKY, M., TAIN, F., GAYM, A., KARIM, A., CARNELL, M. & TESFAYE, S. 2010. Responding to the maternal health care challenge: The Ethiopian Health Extension Program. *Ethiopian Journal of Health Development,* 24.

LANGLOIS, É. V., MISZKURKA, M., ZUNZUNEGUI, M. V., GHAFFAR, A., ZIEGLER, D. & KARP, I. 2015. Inequities in postnatal care in low-and middle-income countries: a systematic review and meta-analysis. *Bulletin of the World Health Organization,* 93**,** 259-270G.

LASATER, T. M., BECKER, D. M., HILL, M. N. & GANS, K. M. 1997. Synthesis of findings and issues from religious-based cardiovascular disease prevention trials. *Annals of Epidemiology,* 7**,** S46-S53.

LAU, L. L., DODD, W., QU, H. L. & COLE, D. C. 2020. Exploring trust in religious leaders and institutions as a mechanism for improving retention in child malnutrition interventions in the Philippines: a retrospective cohort study. *BMJ open,* 10**,** e036091.

LAWN, J. E., MWANSA-KAMBAFWILE, J., HORTA, B. L., BARROS, F. C. & COUSENS, S. 2010. ‘Kangaroo mother care’to prevent neonatal deaths due to preterm birth complications. *International journal of epidemiology,* 39**,** i144-i154.

LUMPKINS, C. Y., GREINER, K. A., DALEY, C., MABACHI, N. M. & NEUHAUS, K. 2013. Promoting healthy behavior from the pulpit: Clergy share their perspectives on effective health communication in the African American church. *Journal of religion and health,* 52**,** 1093-1107.

MAIWADA, A. M., RAHMAN, N. A. A., ABDURRAHAMAN, S., MAMAT, N. M. & WALKER, J. 2016. The Islamic religious leaders as health promoters: Improving maternal health in selected communities of Zamfara State, Nigeria. *J Reprod Infertil,* 7**,** 8-14.

MAKATE, M. & MAKATE, C. 2017. Prenatal care utilization in Zimbabwe: Examining the role of community-level factors. *Journal of epidemiology and global health,* 7**,** 255-262.

MATERNAL, J. 2004. neonatal health: Monitoring birth preparedness and complication readiness, tools and indicators for maternal and newborn health. Johns Hopkins, Bloomberg school of Public Health. *Center for communication programs, Family Care International*.

MEDHANYIE, A., SPIGT, M., DINANT, G. & BLANCO, R. 2012. Knowledge and performance of the Ethiopian health extension workers on antenatal and delivery care: a cross-sectional study. *Human resources for health,* 10**,** 1-8.

MEKONNEN, T., DUNE, T., PERZ, J. & OGBO, F. A. 2019. Trends and determinants of antenatal care service use in Ethiopia between 2000 and 2016. *International journal of environmental research and public health,* 16**,** 748.

MEKONNEN, W. & GEBREMARIAM, A. 2018. Causes of maternal death in Ethiopia between 1990 and 2016: systematic review with meta-analysis. *Ethiopian Journal of Health Development,* 32.

MEKONNEN, Y. & MEKONNEN, A. 2003. Factors influencing the use of maternal healthcare services in Ethiopia. *Journal of health, population and nutrition***,** 374-382.

MOYER, C. A., DAKO-GYEKE, P. & ADANU, R. M. 2013. Facility-based delivery and maternal and early neonatal mortality in sub-Saharan Africa: a regional review of the literature. *African journal of reproductive health,* 17**,** 30-43.

MUKONKA, P. S., MUKWATO, P. K., KWALEYELA, C. N., MWEEMBA, O. & MAIMBOLWA, M. 2018. Household factors associated with use of postnatal care services. *African Journal of Midwifery and Women's Health,* 12**,** 189-193.

NIGUSIE, A., AZALE, T. & YITAYAL, M. 2020. Institutional delivery service utilization and associated factors in Ethiopia: a systematic review and META-analysis. *BMC pregnancy and childbirth,* 20**,** 1-25.

OKEDO-ALEX, I. N., AKAMIKE, I. C., EZEANOSIKE, O. B. & UNEKE, C. J. 2019. Determinants of antenatal care utilisation in sub-Saharan Africa: a systematic review. *BMJ open,* 9**,** e031890.

OLIVIER, J. 2016. Interventions with local faith communities on immunization in development contexts. *The Review of Faith & International Affairs,* 14**,** 36-50.

ONONOKPONO, D. N., ODIMEGWU, C. O., IMASIKU, E. & ADEDINI, S. 2013. Contextual determinants of maternal health care service utilization in Nigeria. *Women & health,* 53**,** 647-668.

OSTROWSKI, C. 2011. Engaging Faith-Based Organizations in the Response to Maternal Mortality.

OUSMAN, S. K., MDALA, I., THORSEN, V. C., SUNDBY, J. & MAGNUS, J. H. 2019. Social determinants of antenatal care service use in Ethiopia: changes over a 15-year span. *Frontiers in public health,* 7**,** 161.

PATTERSON, S. C. A. J. 2007. Faith-Based Models for improving Maternal and Newborn Health.

PELL, C., MEÑACA, A., WERE, F., AFRAH, N. A., CHATIO, S., MANDA-TAYLOR, L., HAMEL, M. J., HODGSON, A., TAGBOR, H. & KALILANI, L. 2013. Factors affecting antenatal care attendance: results from qualitative studies in Ghana, Kenya and Malawi. *PloS one,* 8**,** e53747.

PETERSON, J., ATWOOD, J. R. & YATES, B. 2002. Key elements for church‐based health promotion programs: outcome‐based literature review. *Public Health Nursing,* 19**,** 401-411.

PLUS., F. C. 2017. Engaging Religious Leaders in Support of Maternal Health in Uganda. New York: EngenderHealth/Fistula Care Plus.

PRATA, N., BELL, S. & WEIDERT, K. 2013. Prevention of postpartum hemorrhage in low-resource settings: current perspectives. *International journal of women's health,* 5**,** 737.

PROBANDARI, A., ARCITA, A., KOTHIJAH, K. & PAMUNGKASARI, E. P. 2017. Barriers to utilization of postnatal care at village level in Klaten district, central Java Province, Indonesia. *BMC health services research,* 17**,** 1-9.

RADOVICH, E., BENOVA, L., PENN-KEKANA, L., WONG, K. & CAMPBELL, O. M. R. 2019. ‘Who assisted with the delivery of (NAME)?’Issues in estimating skilled birth attendant coverage through population-based surveys and implications for improving global tracking. *BMJ global health,* 4**,** e001367.

REPORT, U. C. H. S. 2009. Muslim Religious Leaders as Partners in Fostering Positive Reproductive Health and Family Planning Behaviors in Yemen: A Best Practice.

RONSMANS, C., GRAHAM, W. J. & GROUP, L. M. S. S. S. 2006. Maternal mortality: who, when, where, and why. *The lancet,* 368**,** 1189-1200.

RUARK, A., KISHOYIAN, J., BORMET, M. & HUBER, D. 2019. Increasing family planning access in Kenya through engagement of faith-based health facilities, religious leaders, and community health volunteers. *Global Health: Science and Practice,* 7**,** 478-490.

SAY, L., CHOU, D., GEMMILL, A., TUNÇALP, Ö., MOLLER, A.-B., DANIELS, J., GÜLMEZOGLU, A. M., TEMMERMAN, M. & ALKEMA, L. 2014. Global causes of maternal death: a WHO systematic analysis. *The Lancet global health,* 2**,** e323-e333.

SHARIF, S. 2005. The Lancet's neonatal survival series. *The Lancet,* 365**,** 1845.

SIMKHADA, B., TEIJLINGEN, E. R. V., PORTER, M. & SIMKHADA, P. 2008. Factors affecting the utilization of antenatal care in developing countries: systematic review of the literature. *Journal of advanced nursing,* 61**,** 244-260.

TEKELAB, T., CHOJENTA, C., SMITH, R. & LOXTON, D. 2019. Factors affecting utilization of antenatal care in Ethiopia: a systematic review and meta-analysis. *PloS one,* 14**,** e0214848.

TESSEMA, Z. T. & TESEMA, G. A. 2020. Pooled prevalence and determinants of skilled birth attendant delivery in East Africa countries: a multilevel analysis of Demographic and Health Surveys. *Italian Journal of Pediatrics,* 46**,** 1-11.

TEY, N.-P. & LAI, S.-L. 2013. Correlates of and barriers to the utilization of health services for delivery in South Asia and Sub-Saharan Africa. *The Scientific World Journal,* 2013.

U.N. 2019. U.N. Interagency Group on Child Mortality Estimates (IGME), Levels and Trends in Child Mortality Report 2019, 2019; WHO, Trends in maternal mortality: 1990 to 2017, 2019.

UN 2015a. The Millennium Development Goals Report 2015.

UN 2015b. Transforming our world: the 2030 Agenda for Sustainable Development, 2015.

UNDERWOOD, C., HENDRICKSON, Z., VAN LITH, L. M., KUNDA, J. E. L. & MALLALIEU, E. C. 2014. Role of community-level factors across the treatment cascade: a critical review. *JAIDS Journal of Acquired Immune Deficiency Syndromes,* 66**,** S311-S318.

UNDERWOOD, C., KAMHAWI, S. & NOFAL, A. 2013. Religious leaders gain ground in the Jordanian family‐planning movement. *International Journal of Gynecology & Obstetrics,* 123**,** e33-e37.

USAID 2020. ETHIOPIA FACT SHEET: MATERNAL AND CHILD HEALTH – OCTOBER 2020.

VOGEL, J. P., BETRÁN, A. P., WIDMER, M., SOUZA, J. P., GÜLMEZOGLU, A. M., SEUC, A., TORLONI, M. R., MENGESTU, T. K. & MERIALDI, M. 2012. Role of faith-based and nongovernment organizations in the provision of obstetric services in 3 African countries. *American journal of obstetrics and gynecology,* 207**,** 495. e1-495. e7.

WANG, H., TESFAYE, R., RAMANA, G. N. & CHEKAGN, C. T. 2016. *Ethiopia health extension program: an institutionalized community approach for universal health coverage*, World Bank Publications.

WARREN, C., DALY, P., TOURE, L. & MONGI, P. 2006. Postnatal care. *Opportunities for Africa’s newborns. Cape Town, South Africa: Partnership for maternal, newborn and child health***,** 79-90.

WHO 2006. World Health Organization. Reproductive Health Indicators. Guidelines for their generation, interpretation and analysis for global monitoring. Geneva; 2006.

WHO 2010. World Health Organization. World Health Statistics 2010 Indicator compendium Interim version.

WHO 2011. The Partnership for Maternal, Newborn & Child Health. 2011. Strategic Framework 2012 to 2015. Geneva, Switzerland: PMNCH.

WHO 2013. WHO Recommendations on Postnatal Care of the Mother and Newborn. Geneva: World Health Organization; 2013 Oct. Available from: <https://www.ncbi.nlm.nih.gov/books/NBK190086/>.

WHO 2015. World Health Organization, Health in 2015, From MDGs to SDGs.

WHO 2016. WHO recommendations on antenatal care for a positive pregnancy experience. Geneva, Switzerland; 2016.

WHO 2019a. Trends in maternal mortality 2000 to 2017: estimates by WHO, UNICEF, UNFPA, World Bank Group and the United Nations Population Division. Geneva: World Health Organization; 2019. Licence: CC BY-NC-SA 3.0 IGO.

WHO 2019b. Trends in maternal mortality: 2000 to 2017: estimates by WHO, UNICEF, UNFPA, World Bank Group and the United Nations Population Division. Geneva:.

WILLIAMS, R. M., GLANZ, K., KEGLER, M. C. & DAVIS, E. 2012. A study of rural church health promotion environments: Leaders’ and members’ perspectives. *Journal of religion and health,* 51**,** 148-160.

WORKU, A. G., YALEW, A. W. & AFEWORK, M. F. 2013. Factors affecting utilization of skilled maternal care in Northwest Ethiopia: a multilevel analysis. *BMC international health and human rights,* 13**,** 1-11.

ZELALEM AYELE, D., BELAYIHUN, B., TEJI, K. & ADMASSU AYANA, D. 2014. Factors affecting utilization of maternal health Care Services in Kombolcha District, eastern Hararghe zone, Oromia regional state, eastern Ethiopia. *International scholarly research notices,* 2014.

# APPENDICES

**PARTICIPANT INFORMATION SHEET AND INFORMED VOLUNTARY CONSENT FORM**

My name is----------------------------------------------. I am working as a data collector for the study being conducted in this community by Abinet Arega for Fulfilment of PhD in Public Health/Health Communication and Health Behaviour at Jimma University Institute of Health, Faculty of Public Health. I kindly request you to lend me your attention to explain you about the study and being selected as the study participant.

The study title is: Effectiveness of trained religious leaders’ engagement in maternal health education on maternal health service utilizations: cluster randomized controlled trial in Hadiya zone, Southern Ethiopia.

**Purpose of the study**

The Aim of this study is to identify Effectiveness of trained religious leaders’ engagement in maternal health education on maternal health service utilizations in Hadiya zone, Southern Ethiopia**,** knowing this have great importance for the woreda health office to plan intervention programs.

**Procedure and duration:**

I will be interviewing you using a questionnaire to provide me with pertinent data that is helpful for the study. There are **53** questions to answer where I will fill the questionnaire by interviewing you. The interview will take about **45** minutes, so I kindly request you to spare me this time for the interview.

**Risks and benefits**

The risk of being participating in this study is very minimal, but only taking few minutes from your time. There would not be any direct payment for participating in this study. But the findings from this research may reveal important information for the local health planners. And health education related to the study will be given for study participants.

**Confidentiality**

The information you will provide us will be confidential. There will be no information that will identify you in particular. The findings of the study will be general for the study community and will not reflect anything particular of individual persons or housing. The questionnaire will be coded to exclude showing names. No reference will be made in oral or written reports that could link participants to the research.

**Rights**

Participation for this study is fully voluntary. You have the right to declare to participate or not in this study. If you decide to participate, you have the right to withdraw from the study at any time and this will not label you for any loss of benefits which you otherwise are entitled. You do not have to answer any question that you do not want to answer.

**Contact address**

If there are any questions or enquires any time about the study or the procedures, please contact in this address.

Principal investigator: Abinet Arega, email abinetarege@gmail.com or

Mobile phone: 0916100682

Are the information/ objective clear? 1) Yes 2) No

If the answer is “no” elaborate again.

Are you willing to participate in the interview? It is up to you to decide

1) Yes 2) No

Thank you!

**Declaration of informed voluntary consent**

I have read/ was read to me the participant information sheet. I have clearly understood the purpose of the research, the procedures, the risks and benefits, issues of confidentiality, the rights of participating and the contact address for any queries. I have been given the opportunity to ask questions for things that may have been unclear. I was informed that I have the right to withdraw from the study at any time or not to answer any question that I do not want. Therefore, I declare my voluntary consent to participate in this study with my signature as indicated below.

Name of the participant: ..........................

Signature of participant: -------------------- Signature of data collector ---------------------------

# **Annex 1: Questionnaire** for quantitative part

D**eveloped to collect baseline and end-line data for the project entitled** Effectiveness of trained religious leaders’ engagement in maternal health education on maternal health service utilizations: Evidence from cluster randomized controlled trial study in Misha District, Hadiya Zone, and Southern Ethiopia

General Information

Name of Kebele: ________________

Name of Gote __________________

House number ____________________

Family Id ____________________

Individual Id __________________

Date of interview: ____________time interview started _____________ ended at ____________

Respondent available on: 1. 1st visit.

2^nd^ visit.

3^rd^ visit.

Status of interview: 1. Completed. 2. Partially completed (refused in the middle).

Refused: 4. Candidate was absent in 3 visits:

11. Interviewer‘s name: --------------------------------------------Signature: -------------------

12. Supervisor who checked questionnaire for completeness and accuracy:

Name: --------------------------------------------Signature: -------------------------

Date-------------------------

**PART I: SOCIO-DEMOGRAPHIC CHARACTERISTICS OF RESPONDENT**

| **Sir. No.** | **Questions** | **Responses** | **Skip to** |
| --- | --- | --- | --- |
| 101 | Age in complete year | _____________ Years |  |
| 102 | What is your ethnicity? | 1. Hadiya 2. Gurage 3. Kambata 4. Silte 5. Other, specify |  |
| 103 | What is your religion? | 1. Protestant 2. Orthodox 3. Muslim 4. Catholic 5. Other specify |  |
| 104 | Source of income | --------- |  |
| 105 | Monthly income | --------Birr |  |
| 106 | Average distance from health center | In meter------------- |  |
| 107 | Average distance from hospital | In meter------------- |  |
|  | Did you pay for maternal health care services? | 1. Yes 2. No |  |
| 108 | What is the highest education level you have attained? | 1. Illiterate 2.Only read and write (no formal education) 3.Grade 1-4 4. grade 5-8  5. Grade 9-10 6. 11 – 12 grade 7. Above 12th grade |  |
| 109 | What is your occupation? | 1. Housewife 2. Farmer  3. Governmental employee 4. Merchant  5. Housemaid 6.Other (specify) |  |
| 110 | What is your marital status? | 1.Not ever married  2. Married  3.Divorced  4.Separated  5.Widowed  6.Other (specify) _______ |  |
| 111 | Your husband‘s age in complete years |  |  |
| 112 | Your husband‘s occupation | 1. Governmental employee 2. Merchant  3. Farmer 4. Other (specify) |  |
| 113 | Your husband‘s educational status | 1.Illiterate 2.Only read and write (no formal  education)  3.Grade 1-4 4.grade 5-8  5. Grade 9-10 6. Above 10 – 12 grade  7.above 12th grade |  |
| 114 | Family size |  |  |

**PART II: REPRODUCTIVE HISTORY OF RESPONDENT**

| **Sir. No.** | **Questions** | **Responses** | **Skip to** |
| --- | --- | --- | --- |
| **201** | How many months pregnant are you?  Record number of completed months |  |  |
| **202** | Is this your first pregnancy? |  |  |
| **203** | Number of pregnancies |  |  |
| **204** | What was your age at your first pregnancy? | 1. _________ Yrs  2. I don‘t remember |  |
| **205** | Number of children born alive | Female ______ male ______ |  |
| **206** | Number of still birth | Female ______ male ______ |  |
| **207** | Number of abortion |  |  |
| **208** | How many of them were delivered at home? | 1. Live birth number______  2. Still birth number ____ |  |
| **209** | How many of them were delivered in health institution? | 1. Live birth number______  2. Still birth number ____ |  |
| **210** | Have you experienced death of neonate (age less than seven days)? | 1. Yes  2. No  3. I don‘t remember |  |
| **211** | If yes to Q, 210 how many times? |  |  |
| **212** | If yes to Q 210, where was the neonate born? | 1. Home  2. Health institution  3. Other (specify) ___________ |  |
| **213** | How many times have you given birth to a child  in the past five years? |  |  |
| **214** | When was your last child born? | ___________/_________  Month year |  |
| **215** | Where does the last baby belong in your birth order? |  |  |

**PART III MATERNAL HEALTH SERVICE UTILIZATION**

| **Sir. No.** | **Questions** | **Responses** | **Skip to** |
| --- | --- | --- | --- |
| 301 | Is your current pregnancy planned? | 1. Yes 2. No |  |
| 302 | What was your feeling when you recognized your last pregnancy | 1. Happiness  2. Sadness  3. Nothing  4. Other (specify)_________________ |  |
| 303 | Did you face any health problem in this pregnancy? |  |  |
| 304 | If yes to Q 303, what were they? (*Do not read the choices*) | 1. Vaginal bleeding  2. Sever head ache  3. Face/ hand swelling  4. Persistent vomiting  5. Hypertension  6. Fit  7. Other (specify)_______________ |  |
| 305 | Did you visit a health facility during in this pregnancy? | 1. Yes  2. No |  |
| 306 | If yes to Q305, reason for visit | 1. Pregnancy related health problem  2. Health problems not related to pregnancy  3. For antenatal care  4. Other (specify) -------------------------- |  |
| 307 | Did you receive any antenatal care in this pregnancy? | 1. Yes  2. No |  |
| 308 | If you did not attend antenatal care, can you tell me the reasons? (Multiple answer is possible)  **(Do not read the choice)** | 1.No or little Knowledge about ANC  2. No health problem encountered  3. Health institution is too far from my home  4. Expense to ANC is unaffordable.  5.long waiting time  6.Poor handling by health care providers  7. Lack of transportation  8. Lack of time to go to health institution  9. Other (specify)---------------------- |  |
| 309 | If yes to Q 308, at what gestational age did you start pregnancy check-up? | 1. One to three months  2. Four to six months  3. Seven to nine months  4. I don‘t remember |  |
| 310 | How many times did you go for pregnancy check-up? | 1.One time  2.Two times  3.Three times  4.More than three times  5.Don't remember |  |
| 311 | To which institution did you go for  Antenatal care service? | 1. Hospital 2. Health center  3. Health station  4. Other (specify) ________________ |  |
| 312 | Why did you prefer this institution? | 1. Close to my house  2. Competent health worker  3. Fair price  Other (specify) _________ |  |
| 313 | Was health education given during each visit? | 1. Yes, always  2. Yes, sometimes  3. Not at all  4. Don‘t remember |  |
| 314 | If yes to Q 313, were you informed about danger  signs related to pregnancy | 1.Yes  2. No  3. Don‘t remember |  |
| 315 | If Yes to Q 314, Which danger signs were you informed about?  (Don‘t read the choices) | 1. Vaginal bleeding  2. Sever head ache  3. Face/ hand swelling  4. Persistent vomiting  5. Hypertension  6. Other (specify)_________________ |  |
| 316 | Were you informed about where to deliver your baby? | 1. Yes  2. No |  |
| 317 | If yes to Q 316, where were you recommended to deliver? | 1. Home  2. Health facility  3. Other (specify) _________________ |  |
| 318 | Were you informed about who should attend you during delivery? | 1. Yes  2. No |  |
| 319 | If yes to Q 320, who was recommended to attend your delivery? | 1. Trained traditional birth attendant  2. Relative  3. Health professional  4. Other (specify) |  |
| 320 | Were you informed to prepare the following  items before your delivery date | 1. Transport  2. Save money  3. Identify blood donor  4. nothing |  |
| 321 | Were you given an injection in the arm to  prevent you getting tetanus (USE LOCAL  TERM FOR TETANUS)? | 1. Yes  2. No |  |
| 322 | If yes to Q 321, how many times? |  |  |
| 323 | Do you have the card  (From the card register date of injection) | 1. TT1 ____/____/________  Date month year  2. TT2 ____/____/________  Date month year  3. TT3 ____/____/________  Date month year  4. TT4 ____/____/________  Date month year  5. TT5 ____/____/________  Date month year |  |
| 324 | Where did you deliver your last child? | 1. Home  2. Hospital  3. Health center  4. Health station  Other specify _________ |  |
| 325 | Have you smoked or been exposed to second-hand smoke during pregnancy? | 1. Yes  2. No |  |
| 326 | Have you been exposed to pesticides or other toxic chemicals during pregnancy? | 1. Yes  2. No |  |
| 327 | Have you been exposed to X-rays or other non-medical radiological substances during pregnancy? | 1. Yes  2. No |  |
| 328 | Have you taken folic acid during pregnancy? | 1. Yes  2. No |  |
| 329 | Have you consumed alcohol during pregnancy? | 1. Yes  2. No |  |

**Part IV Birth preparedness and complication readiness**

| **Sir. No.** | **Questions** | **Responses** |  |
| --- | --- | --- | --- |
| 401 | Have you ever heard the term “birth preparedness”? | 1. Yes  2. No  3. Do not remember |  |
| 402 | In your opinion, what are some things a woman can do to prepare for birth | 1. IDENTIFY MODE OF TRANSPORT, 1. Yes, 2. No 2. SAVE MONEY, 1. Yes, 2. No 3. IDENTIFY BLOOD DONOR, 1. Yes, 2. No 4. IDENTIFY SKILLED PROVIDER, 1. Yes, 2. No 5. Support person, 1. Yes, 2. No 6. Other specify |  |
| 404 | Does your community provide services to assist women in preparing for birth? For instance, are there:  Transportation services for women?  Ways to get money to help families pay for birth?  Ways to get blood donated during pregnancy or complications?  Any other services | 1. IDENTIFY MODE OF TRANSPORT, 1. Yes, 2. No 2. SAVE MONEY, 1. Yes, 2. No 3. IDENTIFY BLOOD DONOR, 1. Yes, 2. No 4. IDENTIFY SKILLED PROVIDER, 1. Yes, 2. No 5. Support person, 1. Yes, 2. No 6. Other specify--------- |  |

**Part V Knowledge of obstetric danger signs**

| **Sir. No.** | **Questions** | | **Responses** | **Skip to** |
| --- | --- | --- | --- | --- |
| 501 | In your opinion, can unforeseen problems related to pregnancy occur during any pregnancy or childbirth that could endanger the life of a woman? | | 1. Yes 2. No 3. I don’t know |  |
| 502 | In your opinion, what are some serious | | 1. Bleeding , 1. Yes, 2. No 2. Severe headache, 1. Yes, 2. No 3. Blurred vision,1. Yes, 2. No 4. Convulsions, 1. Yes, 2. No 5. Swollen hands/face, 1. Yes, 2. No 6. High fever, 1. Yes, 2. No 7. Loss of consciousness, 1. Yes, 2. No 8. Difficulty breathing, 1. Yes, 2. No 9. Severe weakness , 1. Yes, 2. No 10. Severe abdominal pain, 1. Yes, 2. No 11. Accelerated/ reduced, 1. Yes, 2. No 12. Fetal movement, 1. Yes, 2. No 13. Water breaks without labor, 1. Yes, 2. No 14. other specify 15. None 16. I don’t know |  |
| 503 | In your opinion, what are some serious health problems that can occur during labor and childbirth that could endanger the life of a pregnant woman?  PROBE: Any others? | | 1. Severe bleeding, 1. Yes, 2. no 2. Severe headache, 1. Yes, 2. no 3. Convulsions, 1. Yes, 2. no 4. High fever, 1. Yes, 2. no 5. Loss of consciousness, 1. Yes, 2. no 6. Labor lasting >12 hours, 1. Yes, 2. no 7. Placenta not delivered 30 minutes after baby, 1. Yes, 2. no 8. other___ (specify) 9. none 10. don’t know |  |
| 504 | In your opinion, could a woman die from [this problem] any of these problems? | 1. Yes 2. No | |  |
| 505 | In your opinion, what are some serious health problems that can occur during the first 2 days after birth that could endanger the life of the woman?  PROBE: Any others? | 1. Difficult or fast breathing, 1. Yes, 2. No 2. Yellow skin/eye color (jaundice), 1. Yes, 2. No 3. Poor sucking or feeding, 1. Yes, 2. No 4. Pus, bleeding, or discharge from around the umbilical cord, 1. Yes, 2. No 5. Baby very small, 1. Yes, 2. No 6. Skin lesions or blisters, 1. Yes, 2. No 7. Convulsions/spasms/ rigidity, 1. Yes, 2. No 8. Lethargy/ unconsciousness, 1. Yes, 2. No 9. Red or swollen eyes with pus, 1. Yes, 2. No 10. other (specify)____________ 11. none | |  |
| **506** | In your opinion, could a newborn baby die from [this problem] any of these  Problems? | 1. Yes 2. No 3. I don’t know | |  |
| **507** | Could you name some types of basic care that can be provided to a newborn baby  Immediately after birth? | 1. Exclusive breastfeeding, 1. Yes, 2. No 2. Dry and wrap 1. Yes, 2. No 3. Eye care, 1. Yes, 2. No 4. Cord care, 1. Yes, 2. No 5. other (specify)_______ 6. don’t know | |  |

**Part VI Perception of Pregnancy Risk**

1. The risk for myself during this pregnancy is:

No Risk Extremely

At All High Risk

2. The risk for my unborn baby during this pregnancy is:

No Risk Extremely

At All High Risk

3. My risk of haemorrhaging (losing too much blood) during this pregnancy is:

No Risk Extremely

At All High Risk

4. My risk of having a caesarean section is:

No Risk Extremely

At All High Risk

5. My risk of dying during this pregnancy is:

No Risk Extremely

At All High Risk

6. My baby’s risk of being born prematurely is:

No Risk Extremely

At All High Risk

7. My baby’s risk of having a birth defect is:

No Risk Extremely

At All High Risk

8. My baby’s risk of needing to go to the Neonatal Intensive Care Unit is:

No Risk Extremely

At All High Risk

9. My baby’s risk of dying during this pregnancy is:

No Risk Extremely

At All High Risk

**Part VII General Self-Efficacy Scale**

|  | Not at  all true | Hardly  true | Moderately  true | Exactly  true |
| --- | --- | --- | --- | --- |
| 1. I can always manage to solve difficult problems if I try hard enough | □ | □ | □ | □ |
| 2. If someone opposes me, I can find the means and ways to get what I want. | □ | □ | □ | □ |
| 3. It is easy for me to stick to my  aims and accomplish my goals. | □ | □ | □ | □ |
| 4. I am confident that I could deal efficiently with unexpected events. | □ | □ | □ | □ |
| 5. Thanks to my resourcefulness, I know how to handle unforeseen situations. | □ | □ | □ | □ |
| 6. I can solve most problems if I invest the necessary effort. | □ | □ | □ | □ |
| 7. I can remain calm when facing difficulties because I can rely on my coping abilities. | □ | □ | □ | □ |
| 8. When I am confronted with a problem, I can usually find several solutions. | □ | □ | □ | □ |
| 9. If I am in trouble, I can usually think of a solution | □ | □ | □ | □ |
| 10. I can usually handle whatever comes my way. | □ | □ | □ | □ |

**Part VIII Attitude towards safe delivery utilization**

| 701 | Some people believe that any pregnant woman can develop delivery complication | 1. Strongly disagree; 2. Disagree; 3.Neutral; 4. Agree; 5. Strongly agree |  |
| --- | --- | --- | --- |
| 702 | Some people feel that delivery complications can be dangerous to the health of a woman | 1. Strongly disagree; 2. Disagree; 3.Neutral; 4. Agree; 5. Strongly agree |  |
| 703 | It is believed that delivery complications can‘t be dangerous to the health of the new born | 1. Strongly disagree; 2. Disagree; 3.Neutral; 4. Agree; 5. Strongly agree |  |
| 706 | According to some people‘s belief a woman should plan ahead of time where she will give birth to her baby. | 1. Strongly disagree; 2. Disagree; 3.Neutral; 4. Agree; 5. Strongly agree |  |
| 707 | Some women feel that they shouldn‘t plan ahead of time how they will get to the place where they will give birth. | 1. Strongly disagree; 2. Disagree; 3.Neutral; 4. Agree; 5. Strongly agree |  |
| 708 | Some women feel that every pregnant woman  need a skilled care at delivery | 1. Strongly disagree; 2. Disagree; 3.Neutral; 4. Agree; 5. Strongly agree |  |
| 709 | Few women feel that being attended by male  health personnel during delivery is unethical and shame | 1. Strongly disagree; 2. Disagree; 3.Neutral; 4. Agree; 5. Strongly agree |  |
| 710 | According to the feeling of some pregnant  women it is very shameful to deliver on  delivery bed in labor ward | 1. Strongly disagree; 2. Disagree; 3.Neutral; 4. Agree; 5. Strongly agree |  |
| 711 | Many women believe that women do not go to  a health facility for delivery, mainly because it is too expensive. | 1. Strongly disagree; 2. Disagree; 3.Neutral; 4. Agree; 5. Strongly agree |  |
| 712 | Many women believe that women do not go to  a health facility for delivery because health personnel do not treat them respectfully  . | 1. Strongly disagree; 2. Disagree; 3.Neutral; 4. Agree; 5. Strongly agree |  |

**Part IX Religious leaders’ engagement scale measurement tool (end-line)**

| 801 | My religious leader was consistent reminder so that I keep ANC follow up based on schedule or appointment | 1. Strongly disagree; 2. Disagree; 3.Neutral; 4. Agree; 5. Strongly agree |  |
| --- | --- | --- | --- |
| 802 | My religious leaders frequently reminded to complete taking iron-folate without interruption | 1. Strongly disagree; 2. Disagree; 3.Neutral; 4. Agree; 5. Strongly agree |  |
| 803 | My religious leaders repeatedly notified us about tetanus toxoid vaccination | 1. Strongly disagree; 2. Disagree; 3.Neutral; 4. Agree; 5. Strongly agree |  |
| 804 | My religious leaders repeatedly notified us about STI and HIV screening and counselling | 1. Strongly disagree; 2. Disagree; 3.Neutral; 4. Agree; 5. Strongly agree |  |
| 805 | My religious leaders commonly prompted us in identifying skilled provider | 1. Strongly disagree; 2. Disagree; 3.Neutral; 4. Agree; 5. Strongly agree |  |
| 806 | My religious leaders commonly encouraged us to give birth at health facility | 1. Strongly disagree; 2. Disagree; 3.Neutral; 4. Agree; 5. Strongly agree |  |
| 807 | My religious leaders commonly encouraged us to prepare transportation during labor | 1. Strongly disagree; 2. Disagree; 3.Neutral; 4. Agree; 5. Strongly agree |  |
| 808 | My religious leaders commonly encouraged us to identify sources of support for me and my family during the birth and the immediate postnatal period | 1. Strongly disagree; 2. Disagree; 3.Neutral; 4. Agree; 5. Strongly agree |  |
| 809 | My religious leaders commonly encouraged us to prepare any additional costs associated with the birth, preparing supplies for my care and the care of my new-born baby. | 1. Strongly disagree; 2. Disagree; 3.Neutral; 4. Agree; 5. Strongly agree |  |
| 810 | My religious leaders repeatedly notified us about danger signs during pregnancy | 1. Strongly disagree; 2. Disagree; 3.Neutral; 4. Agree; 5. Strongly agree |  |
| 811 | My religious leaders repeatedly notified us about danger signs during labour and child birth | 1. Strongly disagree; 2. Disagree; 3.Neutral; 4. Agree; 5. Strongly agree |  |
| 812 | My religious leaders repeatedly notified us about danger signs during post-partum period | 1. Strongly disagree; 2. Disagree; 3.Neutral; 4. Agree; 5. Strongly agree |  |
| 813 | My religious leaders repeatedly notified us about new-born danger signs | 1. Strongly disagree; 2. Disagree; 3.Neutral; 4. Agree; 5. Strongly agree |  |
| 814 | My religious leaders reminded us to early seek healthcare when any danger signs occurred during pregnancy, labour and child birth, and new-born, post-partum period | 1. Strongly disagree; 2. Disagree; 3.Neutral; 4. Agree; 5. Strongly agree |  |
| 815 | My religious leaders commonly encouraged us to follow postnatal care services | 1. Strongly disagree; 2. Disagree; 3.Neutral; 4. Agree; 5. Strongly agree |  |
| 816 | My religious leaders commonly notified us on family planning and immunization of baby | 1. Strongly disagree; 2. Disagree; 3.Neutral; 4. Agree; 5. Strongly agree |  |
| 817 | My religious leaders commonly notified us on new-born care | 1. Strongly disagree; 2. Disagree; 3.Neutral; 4. Agree; 5. Strongly agree |  |
| **818** | My religious leaders commonly disseminated MCH messages on congregation | 1. Strongly disagree; 2. Disagree; 3.Neutral; 4. Agree; 5. Strongly agree |  |
| **819** | Religious provided support that is important to maternal and child health promotion | 1. Strongly disagree; 2. Disagree; 3.Neutral; 4. Agree; 5. Strongly agree |  |
| **820** | I feel helped by religious leaders during my pregnancy and child birth | 1. Strongly disagree; 2. Disagree; 3.Neutral; 4. Agree; 5. Strongly agree |  |
| **821** | I feel helped by religious leaders by encouraging to receive MCH care regularly during my pregnancy and child birth | 1. Strongly disagree; 2. Disagree; 3.Neutral; 4. Agree; 5. Strongly agree |  |
| **822** | I feel supported by religious leaders as they initiate to seek healthcare whenever any danger signs is present | 1. Strongly disagree; 2. Disagree; 3.Neutral; 4. Agree; 5. Strongly agree |  |
| **823** | I feel to have been offered support by religious leaders regarding MCH | 1. Strongly disagree; 2. Disagree; 3.Neutral; 4. Agree; 5. Strongly agree |  |
| **824** | I believe that religious leaders are knowledgeable about MCH | 1. Strongly disagree; 2. Disagree; 3.Neutral; 4. Agree; 5. Strongly agree |  |
| **825** | I perceive that religious leaders are welcomed by pregnant mothers as they transmit MCH messages  I believe that religious leaders are credible contributors to maternal and child health promotion | 1. Strongly disagree; 2. Disagree; 3.Neutral; 4. Agree; 5. Strongly agree |  |
| **826** | I believe that religious leaders are trusted while teaching & giving information maternal and child health | 1. Strongly disagree; 2. Disagree; 3.Neutral; 4. Agree; 5. Strongly agree |  |
| **827** | I perceive that religious leaders were following whether or not maternal health services were utilized properly and intended | 1. Strongly disagree; 2. Disagree; 3.Neutral; 4. Agree; 5. Strongly agree |  |
| **828** | I perceived that religious leaders took responsibility to follow-up about regular MCH service utilization | 1. Strongly disagree; 2. Disagree; 3.Neutral; 4. Agree; 5. Strongly agree |  |
| **829** | I perceive that religious leaders felt accountable to monitor taking iron-folate drugs as prescribed | 1. Strongly disagree; 2. Disagree; 3.Neutral; 4. Agree; 5. Strongly agree |  |
| **830** | I am impressed and influenced watching my religious leader disseminating MCH messages | 1. Strongly disagree; 2. Disagree; 3.Neutral; 4. Agree; 5. Strongly agree |  |
| **831** | I am convinced to receive MCH care services regularly following what I saw from my religious leaders’ family | 1. Strongly disagree; 2. Disagree; 3.Neutral; 4. Agree; 5. Strongly agree |  |
| **832** | I feel religious leaders can be role model in seeking care when they have an illness | 1. Strongly disagree; 2. Disagree; 3.Neutral; 4. Agree; 5. Strongly agree |  |
| **833** | Religious leaders were observed mobilized by BCC materials to disseminate MCH messages | 1. Strongly disagree; 2. Disagree; 3.Neutral; 4. Agree; 5. Strongly agree |  |
| **834** | In our church/mosque religious leaders were mostly observed in that they kept promoting maternal and child health | 1. Strongly disagree; 2. Disagree; 3.Neutral; 4. Agree; 5. Strongly agree |  |
| **835** | Religious leaders were mostly observed in that they discourage harmful traditional practices related to pregnancy, Labor and Child Birth | 1. Strongly disagree; 2. Disagree; 3.Neutral; 4. Agree; 5. Strongly agree |  |

# Annex 2 Amharic Version

ጅማ ዩኒቨርሲቲ

የጤና እንስትቱት

የህብረተሳብ ጤና ኮሌጅ

የ ጤና, ባህሪ እና ማህበረሰብ ትምህርት ክፍል

በደቡብ ኢትዮጵያ ሀዲያ ዞን በእናቶች ጤና አገልግሎት አጠቃቀም ላይ በእናቶች ጤና ትምህርት ላይ የሰለጠኑ የሃይማኖት መሪዎች ተሳትፎ ውጤታማነት መገምገም ነው::

የስምምነት ቅጽ

ለጥናቱ ተሳታፊዎች ያንብቡ

ውድ እናት

ስሜ ---------------------------------------------ይበላል:: በጅማ ዩኒቨርሲቲ የጤና ግንኙነት እና የጤና ባህሪ የሦስተኛ ዲግሪ ተማሪ የሆነዉ አብነት አረጋ በሚስራው ጥናት ላይ መረጃ ሰብሳቢ ነኘ፡፡ የዚህ ጥናት አላማ በሀድያ ዞን ውስጥ ባለው በእናቶች ጤና አገልግሎት አጠቃቀም ላይ በእናቶች ጤና ትምህርት ላይ የሰለጠኑ የሃይማኖት መሪዎች ተሳትፎ ውጤታማነት መገምገም ነው። በእናቶች ጤና አገልግሎት አጠቃቀም ውስጥ ላሉ ፕሮግራም አውጪዎች በጣም አስፈላጊ የሆኑ አንዳንድ ጥያቄዎችን ልጠይቅህ ነው። ስምዎ በዚህ ቅጽ አይጻፍም እና የሚሰጡት መረጃ በሚስጥር ይጠበቃል። መልስ መስጠት ካልፈለጉ፣ ሁሉም ወይም የተወሰኑት እርስዎ ያቀረቧቸው ጥያቄዎች ይህንን ለማድረግ መብት አሎት። ነገር ግን፣ ሁሉንም ጥያቄዎች ለመመለስ ፈቃደኛነትዎ እና ድጋፍዎ እናመሰግናለን እናም በሁሉም ተሳታፊዎች ስም እናመሰግናለን። ጥያቄ ወይም ችግር ካለ በስልክ ቁጥር 0916-`10-0682 መደወል ይችላሉ፡፡

በጥናቱ ላይ ለመሳተፍ ተሳሞምተዋል? አዎ ------ አልተሰማማሁም ------

ካልተስማሙ እናመሰግናለን (ወደሚቀጥለው እለፍ)

የመጠይቁ መለያ ቁጥር
ቀበሌ

የቤት ቁጥር

የጠያቂ ስምና ፊርማ

ክፍል አንድ፡- ማህበረ-ዲሞክራሲያዊ ምላሽ ሰጪ ባህሪያት

| **Sir. No.** | **ጥያቄዎች** | **ምላሾች** | **Skip to** |
| --- | --- | --- | --- |
| 101 | ሙሉ ዕድሜ ዓመት |  |  |
| 102 | ዘርህ የቱ ነው? | 1. 1.ሀዲያ2.ጉራጌ3.ከምባታ4.ስልጤ5.ሌላ ይግለጹ |  |
| 103 | ሃይማኖትህ ምንድን ነው? | 1. 1.ፕሮቴስታንት2.ኦርቶዶክስ3.ሙስሊም4.ካቶሊክ5.ሌላ ይግለጹ። |  |
| 104 | የገቢ ምንጭ | --------- |  |
| 105 | ወርሃዊ ገቢ | --------Birr |  |
| 106 | ከጤና ጣቢያ አማካኝ ርቀት | በሜትር ------------- |  |
| 107 | ከሆስፒታል አማካይ ርቀት | በሜትር ------------- |  |
|  | ለእናቶች ጤና አገልግሎት ከፍለዋል? | 1. 1.አዎ2.አይ |  |
| 108 | ያገኙት ከፍተኛ የትምህርት ደረጃ ስንት ነው? | 1. መሃይም 2. ማንበብና መጻፍ ብቻ (መደበኛ ትምህርት የለም) 3. ክፍል 1-4 4. 5-8. 5. 9-10 6. 11 - 12 ክፍል 7. ከ12ኛ ክፍል በላይ |  |
| 109 | ሥራህ ምንድን ነው? | 1. የቤት እመቤት 2. ገበሬ 3. የመንግስት ሰራተኛ 4. ነጋዴ 5. የቤት ሰራተኛ 6.ሌላ (ይግለጹ)) |  |
| 110 | የጋብቻ ሁኔታዎ ምን ያህል ነው? | 1. በጭራሽ አላገባም  2. ያገባ  3.የተፋታ  4.የተለየ  5.የሞተባት  6.ሌላ (ይግለጹ) |  |
| 111 | የባልሽ ዕድሜ ሙሉ ዓመታት |  |  |
| 112 | የባልሽ ስራ | 1. የመንግስት ሰራተኛ 2. ነጋዴ 3.ገበሬ 4.ሌላ (ይግለዥ) |  |
| 113 | የባልሽ የትምህርት ደረጃ | 1. መሃይም 2. ማንበብና መጻፍ ብቻ (መደበኛ ትምህርት የለም) 3. ክፍል 1-4 4. 5-8. 5. 9-10 6. 11 - 12 ክፍል 7. ከ12ኛ ክፍል በላይ |  |
| 114 | የቤተሰብ መጠን |  |  |

ክፍል II፡ reproductive ምላሽ ሰጪ ታሪክ

| **Sir. No.** | **ጥያቄዎች** | **ምላሾች** | **Skip to** |
| --- | --- | --- | --- |
| **201** | ስንት ወር ነፍሰ ጡር ነህ?የተጠናቀቁ ወራት ብዛት ይመዝግቡ |  |  |
| **202** | ይህ የመጀመሪያ እርግዝናዎ ነው? |  |  |
| **203** | የእርግዝና ብዛት |  |  |
| **204** | በመጀመሪያው እርግዝናዎ ዕድሜዎ ስንት ነበር? | 1. ________ ዓመቶች 2. **አላስታውስም** |  |
| **205** | በህይወት የተወለዱ ልጆች ቁጥር | ሴት____ ወንድ ______ |  |
| **206** | ገና የተወለዱ ሰዎች ቁጥር | ሴት____ ወንድ ______ |  |
| **207** | የፅንስ ማስወረድ ቁጥር |  |  |
| **208** | ከነሱ ውስጥ ስንቶቹ በቤት ውስጥ ተወለዱ? | 1. የቀጥታ የልደት ቁጥር ______  2. አሁንም የልደት ቁጥር ____ |  |
| **209** | ስንቶቹ በጤና ተቋም ተወለዱ? | 1. የቀጥታ የልደት ቁጥር ______  2. አሁንም የልደት ቁጥር ____ |  |
| **210** | የአራስ ሞት አጋጥሞዎታል (ዕድሜ ከሰባት ቀን በታች)? | 1. አዎ  2. No  3. አላስታውስም። |  |
| **211** | ለ Q 210 አዎ ከሆነ ስንት ጊዜ? |  |  |
| **212** | ለ Q 210 አዎ ከሆነ , አዲስ የተወለደው ሕፃን የት ነው የተወለደው? | 1. ቤት  2. የጤና ተቋም  3. ሌላ (ይግለጹ)___________ |  |
| **213** | ባለፉት አምስት ዓመታት ውስጥ ስንት ጊዜ ልጅ ወለዱ? |  |  |
| **214** | የመጨረሻ ልጅህ መቼ ነው የተወለደው? | ___________/_________  Month year |  |
| **215** | በትውልድ ቅደም ተከተልዎ ውስጥ የመጨረሻው ልጅ የት ነው ያለው? |  |  |

ክፍል III የእናቶች ጤና አገልግሎት አጠቃቀም

| **Sir. No.** | **ጥያቄዎች** | **ምላሾች** | **Skip to** |
| --- | --- | --- | --- |
| 301 | የአሁኑ እርግዝናዎ የታቀደ ነው? | 1. አዎ 2. አይደለም |  |
| 302 | የመጨረሻ እርግዝናዎን ሲያውቁ ምን ስሜት ነበር | 1. ደስታ 2. ሀዘን 3. ምንም 4. ሌላ (ይግለጹ)_____ |  |
| 303 | በዚህ እርግዝና ውስጥ የጤና ችግር አጋጥሞዎታል? | 1. አዎ 2. አይደለም |  |
| 304 | ለ Q 303 አዎ ከሆነ, ምን ነበሩ? (*ምርጫዎቹን አታንብብ*) | 1. የሴት ብልት ደም መፍሰስ  2. የጭንቅላት ሕመምን ያስወግዱ  3. የፊት / የእጅ እብጠት  4. የማያቋርጥ ትውከት  5. የደም ግፊት መጨመር  6. ሌላ (ይግለጹ)_______________ |  |
| 305 | በዚህ እርግዝና ወቅት የጤና ተቋምን ጎበኙ? | 1. አዎ 2. አይደለም |  |
| 306 | ለ Q 305 አዎ ከሆነ, የመጎብኘት ምክንያት | 1. ከእርግዝና ጋር የተያያዘ የጤና ችግር  2. ከእርግዝና ጋር ያልተያያዙ የጤና ችግሮች  3. ለቅድመ ወሊድ እንክብካቤ  4. ሌላ (ይግለጹ) ------------------- |  |
| 307 | በዚህ እርግዝና ውስጥ ምንም አይነት የቅድመ ወሊድ እንክብካቤ አግኝተዋል? | 1. አዎ 2. አይደለም |  |
| 308 | በቅድመ ወሊድ እንክብካቤ ካልተከታተልክ ምክንያቶቹን ንገረኝ? **(ምርጫውን አታንብብ)** | 1. ስለ ቅድመ ወሊድ እንክብካቤ ምንም ወይም ትንሽ እውቀት  2. ምንም የጤና ችግር አጋጥሞታል  3. የጤና ተቋም ከቤቴ በጣም የራቀ ነው  4. ለቅድመ ወሊድ እንክብካቤ የሚወጣው ወጪ ተመጣጣኝ አይደለም  5. ለአገልግሎት ረጅም የጥበቃ ጊዜ  6. በጤና እንክብካቤ አቅራቢዎች ደካማ አያያዝ  7. የመጓጓዣ እጥረት  8. ወደ ጤና ተቋም ለመሄድ ጊዜ ማጣት  9. ሌላ (ይግለጹ) ------------------ |  |
| 309 | ለ Q 305, የመጎብኘት ምክንያት ለቅድመ ወሊድ እንክብካቤ ከሆነ, በየትኛው የእርግዝና እድሜ ላይ የእርግዝና ምርመራ ማድረግ ጀመሩ? | 1. ከአንድ እስከ ሶስት ወር  2. ከአራት እስከ ስድስት ወር  3. ከሰባት እስከ ዘጠኝ ወራት  4. አላስታውስም |  |
| 310 | ለእርግዝና ምርመራ ስንት ጊዜ ሄዱ? | 1. አንድ ጊዜ  2. ሁለት ጊዜ  3. ሦስት ጊዜ  4. ከሶስት እጥፍ በላይ  5. አላስታውስም |  |
| 311 | ለቅድመ ወሊድ አገልግሎት ወደ የትኛው ተቋም ሄዱ? | 1. ሆስፒታል 2. ጤና ጣቢያ  3. ጤና ጣቢያ 4. ሌላ (ይግለጹ)) ________________ |  |
| 312 | ይህንን ተቋም ለምን መረጡት? | 1. ወደ ቤቴ ቅርብ 2. ብቃት ያለው የጤና ሰራተኛ 3. ትክክለኛ ዋጋ፣ ሌላ (ይግለጹ)_________ |  |
| 313 | በእያንዳንዱ ጉብኝት የጤና ትምህርት ተሰጥቷል? | 1. አዎ ሁሌም 2. አዎ አንዳንዴ 3. በፍፁም 4. አታስታውስም። |  |
| 314 | ለ Q 313 አዎ ከሆነ, ከእርግዝና ጋር የተያያዙ አደገኛ ምልክቶችን ይነግሩዎታል | 1.አዎ 2. አይ 3. አላስታውስም |  |
| 315 | ለ Q 314 አዎ ከሆነ, ስለ የትኞቹ የአደጋ ምልክቶች ተነግሯችኋል? (ምርጫዎቹን አታንብብ) | 1. የሴት ብልት ደም መፍሰስ  2. የጭንቅላት ህመም  3. የፊት/የእጅ እብጠት  4. የማያቋርጥ ትውከት  5. የደም ግፊት  6. ሌላ (ይግለጹ)_ ________________ |  |
| 316 | ልጅዎን የት እንደሚወልዱ ተነግሮዎታል? | 1. አዎ 2. አይደለም |  |
| 317 | ለ Q 316 አዎ ከሆነ, የት ለመውለድ ተመክረዋል? | 1. ቤት 2. የጤና ተቋም 3. ሌላ (ይግለጹ)_________________ |  |
| 318 | በወሊድ ጊዜ ማን መገኘት እንዳለበት ተነግሮታል? | 1. አዎ 2. አይደለም |  |
| 319 | ለ Q 316 አዎ ከሆነ, በወሊድዎ ላይ እንዲገኝ የተመከረው ማን ነው? | 1. የሰለጠነ ባህላዊ ወሊድ 2. ዘመድ 3. የጤና ባለሙያ 4. ሌላ (ይግለጹ) |  |
| 320 | የማድረሻ ቀንዎ በፊት የሚከተሉትን ዕቃዎች እንዲያዘጋጁ ተነግሮዎታል | 1. ትራንስፖርት 2. ገንዘብ መቆጠብ 3. ደም ለጋሾችን መለየት 4. ምንም |  |
| 321 | ቴታነስ እንዳንያዝክ ክንድ ላይ መርፌ ተሰጥተሃል (ለቲታነስ የአካባቢ ጊዜን ተጠቀም)? | 1. አዎ 2. አይደለም |  |
| 322 | ለ Q 321አዎ ከሆነ, ስንት ጊዜ? |  |  |
| 323 | ካርዱ አለህ? (ከካርድ መመዝገቢያ ቀን ጀምሮ) | 1. TT1 ____/____/________  ቀን ወር አመት  2. TT2 ____/____/________  ቀን ወር አመት  3. TT3 ____/____/________  ቀን ወር አመት  4. TT4 ____/____/________  ቀን ወር አመት  5. TT5 ____/____/________  ቀን ወር አመት |  |
| 324 | የመጨረሻ ልጅህን የት ነው የወለድከው? | 1. ቤት  2. ሆስፒታል  3. ጤና ጣቢያ  4. ጤና ጣቢያ ሌላ ይግለጹ_________ |  |
| 325 | በእርግዝና ወቅት አጨስ ወይም ለሁለተኛ እጅ ማጨስ ተጋልጧል? | 1. አዎ 2. አይደለም |  |
| 326 | በእርግዝና ወቅት ለፀረ-ተባይ ወይም ለሌሎች መርዛማ ኬሚካሎች ተጋልጠዋል? | 1. አዎ 2. አይደለም |  |
| 327 | በእርግዝና ወቅት ለኤክስሬይ ወይም ለሌሎች ለሕክምና ያልሆኑ ራዲዮሎጂካል ንጥረ ነገሮች ተጋልጠዋል? | 1. አዎ 2. አይደለም |  |
| 328 | በእርግዝና ወቅት ፎሊክ አሲድ ወስደዋል? | 1. አዎ 2. አይደለም |  |
| 329 | በእርግዝና ወቅት አልኮል ጠጥተዋል? | 1. አዎ 2. አይደለም |  |

ክፍል IV የልደት ዝግጁነት እና ውስብስብነት ዝግጁነት

| **Sir. No.** | **ጥያቄዎች** | **ምላሾች** | **Skip to** |
| --- | --- | --- | --- |
| 401 | "የልደት ዝግጁነት" የሚለውን ቃል ሰምተህ ታውቃለህ? | 1. አዎ  2. አይደለም  3. አላስታውስም |  |
| 402 | በእርስዎ አስተያየት አንዲት ሴት ለመውለድ ለማዘጋጀት ምን ማድረግ ትችላለች? | 1) የመጓጓዣ ሁነታን መለየት,  1. አዎ 2. አይደለም   1. ገንዘብ ይቆጥቡ, 2. አዎ 2. አይደለም   3) ደም ለጋሾችን መለየት  1. አዎ 2. አይደለም  4) የሰለጠነ የወሊድ ረዳትን መለየት  1. አዎ 2. አይደለም   1. ደጋፊ ሰው   1. አዎ 2. አይደለም   1. ሌሎች ይግለጹ |  |
| 404 | የእርስዎ ማህበረሰብ ሴቶች ለመውለድ በሚዘጋጁበት ጊዜ ለመርዳት አገልግሎት ይሰጣል? ለምሳሌ፣ አሉ፡- | 1) የመጓጓዣ ሁነታን መለየት,  1. አዎ 2. አይደለም   1. ገንዘብ ይቆጥቡ, 2. አዎ 2. አይደለም   3) ደም ለጋሾችን መለየት  1. አዎ 2. አይደለም  4) የሰለጠነ የወሊድ ረዳትን መለየት  1. አዎ 2. አይደለም   1. ደጋፊ ሰው 2. አዎ 2. አይደለም 3. ሌሎች ይግለጹ |  |

ክፍል V በእርግዝና, በምጥ እና በወሊድ ጊዜ, አዲስ የተወለደ እና የድህረ ወሊድ ጊዜ አደገኛ ምልክቶችን ማወቅ

| **Sir. No.** | **ጥያቄዎች** | | **ምላሾች** | **Skip to** |
| --- | --- | --- | --- | --- |
| 501 | በእርስዎ አስተያየት ከእርግዝና ጋር የተያያዙ ያልተጠበቁ ችግሮች በማንኛውም እርግዝና ወይም ልጅ መውለድ የሴቶችን ሕይወት አደጋ ላይ ሊጥሉ ይችላሉ? | | 1. አዎ  2. አይደለም  3. አላስታውስም |  |
| 502 | በእርስዎ አስተያየት አንዳንድ ከባድ ችግሮች ምንድናቸው? | | 1. የደም መፍሰስ   1. አዎ 2. አይደለም   1. ከባድ ራስ ምታት   1. አዎ 2. አይደለም   1. የደበዘዘ እይታ   1. አዎ 2. አይደለም   1. መንቀጥቀጥ   1. አዎ 2. አይደለም   1. እብጠት እጆች / ፊት   1. አዎ 2. አይደለም   1. ከፍተኛ ትኩሳት   1. አዎ 2. አይደለም   1. የንቃተ ህሊና ማጣት   1. አዎ 2. አይደለም   1. የመተንፈስ ችግር   1. አዎ 2. አይደለም   1. ከባድ ድክመት   1. አዎ 2. አይደለም   1. ከባድ የሆድ ህመም   1. አዎ 2. አይደለም   1. የተፋጠነ / የተቀነሰ የፅንስ እንቅስቃሴ   1. አዎ 2. አይደለም   1. Water breaks without labor   1. አዎ 2. አይደለም   1. ሌሎች ይግለጹ 2. የለም 3. አላውቅም |  |
| 503 | በእርስዎ አስተያየት፣ ነፍሰ ጡር ሴትን ህይወት አደጋ ላይ የሚጥሉ ምጥ እና በወሊድ ወቅት ሊከሰቱ የሚችሉ ከባድ የጤና ችግሮች ምን ምን ናቸው?  PROBE: ሌላስ? | | 1. የደም መፍሰስ   1. አዎ 2. አይደለም   1. ከባድ ራስ ምታት   1. አዎ 2. አይደለም   1. መንቀጥቀጥ   1. አዎ 2. አይደለም   1. ከፍተኛ ትኩሳት   1. አዎ 2. አይደለም   1. የንቃተ ህሊና ማጣት   1. አዎ 2. አይደለም   1. Labor የሚቆይ>12 ሰዓታት   1. አዎ 2. አይደለም   1. Placenta ከተወለደ ከ 30 ደቂቃዎች በኋላ አልተወለደም   1. አዎ 2. አይደለም   1. ሌሎች ይግለጹ 2. የለም 3. አላውቅም |  |
| 504 | በእርስዎ አስተያየት አንዲት ሴት ከእነዚህ ችግሮች ውስጥ በአንዱ ልትሞት ትችላለች? | 1. አዎ 2. አይደለም 3. አላውቅም | |  |
| 505 | እንደ እርስዎ አስተያየት ከተወለዱ በኋላ ባሉት 2 ቀናት ውስጥ የሴቲቱን ህይወት አደጋ ላይ የሚጥሉ አንዳንድ ከባድ የጤና ችግሮች ምን ምን ናቸው?  PROBE: ሌላስ? | 1. አስቸጋሪ ወይም ፈጣን መተንፈስ   1. አዎ 2. አይደለም   1. ቢጫ የቆዳ/የአይን ቀለም (ጃንዲስ)   1. አዎ 2. አይደለም   1. ደካማ መጥባት ወይም መመገብ   1. አዎ 2. አይደለም   1. በእምብርት ገመድ አካባቢ የሚፈስ፣የደም መፍሰስ ወይም ፈሳሽ ፈሳሽ   1. አዎ 2. አይደለም   1. Baby በጣም ትንሽ   1. አዎ 2. አይደለም   1. የቆዳ ቁስሎች ወይም አረፋዎች   1. አዎ 2. አይደለም   1. መንቀጥቀጥ / spasms / ግትርነት   1. አዎ 2. አይደለም   1. ልቅነት / ንቃተ-ህሊና ማጣት   1. አዎ 2. አይደለም   1. ቀይ ወይም ያበጠ አይኖች መግል   1. አዎ 2. አይደለም   1. ሌሎች ይግለጹ_______ 2. የለም 3. አላውቅም | |  |
| **506** | በእርስዎ አስተያየት አዲስ የተወለደ ሕፃን ከእነዚህ ችግሮች በአንዱ ሊሞት ይችላል? | 1. አዎ 2. አይደለም 3. አላውቅም | |  |
| **507** | አዲስ ለተወለደ ሕፃን ወዲያውኑ ከተወለደ በኋላ ሊሰጡ የሚችሉ አንዳንድ መሠረታዊ እንክብካቤ ዓይነቶችን መጥቀስ ይችላሉ? | 1. ልዩ ጡት ማጥባት   1. አዎ 2. አይደለም   1. ደረቅ እና መጠቅለል 1. 1. አዎ 2. አይደለም 2. የአይን እንክብካቤ   1. አዎ 2. አይደለም   1. የገመድ እንክብካቤ 2. 1. አዎ 2. አይደለም 3. ሌሎች ይግለጹ_______ 4. አላውቅም | |  |

**ክፍል VI የእርግዝና ስጋት ግንዛቤ**

1. በዚህ እርግዝና ወቅት ለራሴ ያለው አደጋ፡-

ምንም ስጋት የለም እጅግ በጣም ከፍተኛ ስጋት

1. በዚህ እርግዝና ወቅት የማኅፀን ልጅ ስጋት፡-

ምንም ስጋት የለም እጅግ በጣም ከፍተኛ ስጋት

1. በዚህ እርግዝና ወቅት የደም መፍሰስ ችግር (ብዙ ደም የማጣት) ስጋት፡-

ምንም ስጋት የለም እጅግ በጣም ከፍተኛ ስጋት

1. በቀዶ ሕክምና የመውለድ ዕድሌ፡-

ምንም ስጋት የለም እጅግ በጣም ከፍተኛ ስጋት

1. በዚህ እርግዝና ወቅት የመሞት ዕድሌ፡-

ምንም ስጋት የለም እጅግ በጣም ከፍተኛ ስጋት

1. ልጄ ያለጊዜው የመወለድ አደጋ፡-

ምንም ስጋት የለም እጅግ በጣም ከፍተኛ ስጋት

1. የልጄ የወሊድ ችግር ያለበት አደጋ፡-

ምንም ስጋት የለም እጅግ በጣም ከፍተኛ ስጋት

1. ልጄ ወደ አራስ ሕፃን ከፍተኛ እንክብካቤ ክፍል የመሄድ አደጋ የሚከተለው ነው፡-

ምንም ስጋት የለም እጅግ በጣም ከፍተኛ ስጋት

1. በዚህ እርግዝና ወቅት ልጄ የመሞት አደጋ፡-

እጅግ በጣም ከፍተኛ ስጋት እጅግ በጣም ከፍተኛ ስጋት

ክፍል VII ደህንነቱ የተጠበቀ የወሊድ አገልግሎት አጠቃቀም ላይ ያለ አመለካከት

| 701 | አንዳንድ ሰዎች ማንኛውም ነፍሰ ጡር ሴት የመውለድ ችግር ሊፈጠር ይችላል ብለው ያምናሉ | 1. በጣም አልስማማም, 2. አልስማማም, 3. ገለልተኛ, 4. እስማማለሁ, 5. በጣም እስማማለሁ |  |
| --- | --- | --- | --- |
| 702 | አንዳንድ ሰዎች የወሊድ ችግሮች ለሴት ጤንነት አደገኛ ሊሆኑ እንደሚችሉ ይሰማቸዋል | 1. በጣም አልስማማም, 2. አልስማማም, 3. ገለልተኛ, 4. እስማማለሁ, 5. በጣም እስማማለሁ |  |
| 703 | የወሊድ ችግሮች አዲስ ለተወለደ ሕፃን ጤና አደገኛ ሊሆኑ እንደማይችሉ ይታመናል | 1. በጣም አልስማማም, 2. አልስማማም, 3. ገለልተኛ, 4. እስማማለሁ, 5. በጣም እስማማለሁ |  |
| 706 | በአንዳንድ ሰዎች እምነት አንዲት ሴት ልጇን የምትወልድበትን ቦታ አስቀድመህ ማቀድ አለባት | 1. በጣም አልስማማም, 2. አልስማማም, 3. ገለልተኛ, 4. እስማማለሁ, 5. በጣም እስማማለሁ |  |
| 707 | አንዳንድ ሴቶች ወደሚወልዱበት ቦታ እንዴት እንደሚደርሱ አስቀድመው ማቀድ እንደሌለባቸው ይሰማቸዋል. | 1. በጣም አልስማማም, 2. አልስማማም, 3. ገለልተኛ, 4. እስማማለሁ, 5. በጣም እስማማለሁ |  |
| 708 | አንዳንድ ሴቶች እያንዳንዱ ነፍሰ ጡር ሴት የሰለጠነ የወሊድ እንክብካቤ እንደሚያስፈልገው ይሰማቸዋል | 1. በጣም አልስማማም, 2. አልስማማም, 3. ገለልተኛ, 4. እስማማለሁ, 5. በጣም እስማማለሁ |  |
| 709 | ጥቂት ሴቶች በወሊድ ጊዜ የወንድ ጤና ባለሙያዎች መገኘት ሥነ ምግባር የጎደለው እና አሳፋሪ እንደሆነ ይሰማቸዋል | 1. በጣም አልስማማም, 2. አልስማማም, 3. ገለልተኛ, 4. እስማማለሁ, 5. በጣም እስማማለሁ |  |
| 710 | እንደ አንዳንድ ነፍሰ ጡር እናቶች ስሜት በወሊድ ክፍል ውስጥ አልጋ ላይ መውለድ በጣም አሳፋሪ ነው. | 1. በጣም አልስማማም, 2. አልስማማም, 3. ገለልተኛ, 4. እስማማለሁ, 5. በጣም እስማማለሁ |  |
| 711 | ብዙ ሴቶች ሴቶች ለመውለድ ወደ ጤና ተቋም አይሄዱም ብለው ያምናሉ፣ ይህም በዋነኝነት በጣም ውድ ስለሆነ ነው። | 1. በጣም አልስማማም, 2. አልስማማም, 3. ገለልተኛ, 4. እስማማለሁ, 5. በጣም እስማማለሁ |  |
| 712 | ብዙ ሴቶች ሴቶች ለመውለድ ወደ ጤና ተቋም የማይሄዱት የጤና ባለሙያዎች በአክብሮት ስለማያዩአቸው እንደሆነ ያምናሉ  . | 1. በጣም አልስማማም, 2. አልስማማም, 3. ገለልተኛ, 4. እስማማለሁ, 5. በጣም እስማማለሁ |  |

ክፍል VIII የሃይማኖት መሪዎች የተሳትፎ ልኬት መለኪያ መሣሪያ

| 801 | በቀጠሮ ወይም በቀጠሮ ላይ ተመስርቼ የቅድመ ወሊድ ክትትል እንዳደርግ የሃይማኖት መሪዬ የማያቋርጥ ማሳሰቢያ ነበር። | 1. በጣም አልስማማም, 2. አልስማማም, 3. ገለልተኛ, 4. እስማማለሁ, 5. በጣም እስማማለሁ |  |
| --- | --- | --- | --- |
| 802 | የሀይማኖት መሪዎቼ ብረት ፎሌት ያለማቋረጥ መውሰድ እንዲጨርሱ ደጋግመው ያሳስባሉ | 1. በጣም አልስማማም, 2. አልስማማም, 3. ገለልተኛ, 4. እስማማለሁ, 5. በጣም እስማማለሁ |  |
| 803 | የሃይማኖት መሪዎቼ ስለ ቴታነስ ቶክሳይድ ክትባት ደጋግመው አሳውቀውናል። | 1. በጣም አልስማማም, 2. አልስማማም, 3. ገለልተኛ, 4. እስማማለሁ, 5. በጣም እስማማለሁ |  |
| 804 | የሃይማኖት መሪዎቼ ስለ ግብረ ሥጋ ግንኙነት የሚተላለፉ በሽታዎች እና ስለ ኤችአይቪ ምርመራ እና ምክር ደጋግመው አሳውቀውናል። | 1. በጣም አልስማማም, 2. አልስማማም, 3. ገለልተኛ, 4. እስማማለሁ, 5. በጣም እስማማለሁ |  |
| 805 | የሃይማኖት መሪዎቼ የሰለጠነ የወሊድ አገልግሎትን እንድንለይ ገፋፍተውናል። | 1. በጣም አልስማማም, 2. አልስማማም, 3. ገለልተኛ, 4. እስማማለሁ, 5. በጣም እስማማለሁ |  |
| 806 | የሃይማኖት መሪዎቼ በጤና ተቋም እንድንወልድ ያበረታቱናል። | 1. በጣም አልስማማም, 2. አልስማማም, 3. ገለልተኛ, 4. እስማማለሁ, 5. በጣም እስማማለሁ |  |
| 807 | የሃይማኖት መሪዎቼ በምጥ ጊዜ መጓጓዣ እንድናዘጋጅ ያበረታቱናል። | 1. በጣም አልስማማም, 2. አልስማማም, 3. ገለልተኛ, 4. እስማማለሁ, 5. በጣም እስማማለሁ |  |
| 808 | የሃይማኖት መሪዎቼ በወሊድ ጊዜ እና በድህረ ወሊድ ወቅት ለእኔ እና ለቤተሰቤ የድጋፍ ምንጮችን እንድንለይ በተለምዶ ያበረታቱናል። | 1. በጣም አልስማማም, 2. አልስማማም, 3. ገለልተኛ, 4. እስማማለሁ, 5. በጣም እስማማለሁ |  |
| 809 | የሃይማኖት መሪዎቼ ከወሊድ ጋር የተያያዙ ተጨማሪ ወጪዎችን እንድናዘጋጅ፣ ለእኔ እንክብካቤ እና አዲስ ለተወለደው ልጄ እንክብካቤ የሚያስፈልጉትን ነገሮች እንድናዘጋጅ በተለምዶ ያበረታቱናል። | 1. በጣም አልስማማም, 2. አልስማማም, 3. ገለልተኛ, 4. እስማማለሁ, 5. በጣም እስማማለሁ |  |
| 810 | የሃይማኖት መሪዎቼ በእርግዝና ወቅት ስለ አደገኛ ምልክቶች ደጋግመው አሳውቀውናል። | 1. በጣም አልስማማም, 2. አልስማማም, 3. ገለልተኛ, 4. እስማማለሁ, 5. በጣም እስማማለሁ |  |
| 811 | የሃይማኖት መሪዎቼ በምጥ እና ልጅ በሚወልዱበት ወቅት ስለ አደገኛ ምልክቶች ደጋግመው አሳውቀውናል። | 1. በጣም አልስማማም, 2. አልስማማም, 3. ገለልተኛ, 4. እስማማለሁ, 5. በጣም እስማማለሁ |  |
| 812 | የሃይማኖት መሪዎቼ ከወሊድ በኋላ ባሉት ጊዜያት ስለ አደገኛ ምልክቶች ደጋግመው አሳውቀውናል። | 1. በጣም አልስማማም, 2. አልስማማም, 3. ገለልተኛ, 4. እስማማለሁ, 5. በጣም እስማማለሁ |  |
| 813 | የሃይማኖት መሪዎቼ አዲስ ስለተወለዱ የአደጋ ምልክቶች ደጋግመው አሳውቀውናል። | 1. በጣም አልስማማም, 2. አልስማማም, 3. ገለልተኛ, 4. እስማማለሁ, 5. በጣም እስማማለሁ |  |
| 814 | የሃይማኖት መሪዎቼ በእርግዝና ወቅት፣ ምጥ እና ልጅ በሚወልዱበት ወቅት፣ እና አዲስ በተወለደ፣ ከወሊድ በኋላ በሚከሰት ጊዜ ማንኛውም የአደጋ ምልክቶች ሲከሰቱ ጤናን ቀድመን እንድንፈልግ አስታውሰውናል። | 1. በጣም አልስማማም, 2. አልስማማም, 3. ገለልተኛ, 4. እስማማለሁ, 5. በጣም እስማማለሁ |  |
| 815 | የሀይማኖት መሪዎቼ የድህረ ወሊድ አገልግሎት እንድንከተል ያበረታቱናል። | 1. በጣም አልስማማም, 2. አልስማማም, 3. ገለልተኛ, 4. እስማማለሁ, 5. በጣም እስማማለሁ |  |
| 816 | የኃይማኖት መሪዎቼ ስለ ቤተሰብ ምጣኔ እና ስለ ሕፃን ክትባቶች በተለምዶ ያሳውቁን ነበር። | 1. በጣም አልስማማም, 2. አልስማማም, 3. ገለልተኛ, 4. እስማማለሁ, 5. በጣም እስማማለሁ |  |
| 817 | የሃይማኖት መሪዎቼ አዲስ ስለተወለደው እንክብካቤ ብዙ ጊዜ ያሳውቁን ነበር። | 1. በጣም አልስማማም, 2. አልስማማም, 3. ገለልተኛ, 4. እስማማለሁ, 5. በጣም እስማማለሁ |  |
| **818** | የሀይማኖት መሪዎቼ የእናቶች እና የህፃናት ጤና መልእክቶችን በጉባኤው ላይ ያሰራጫሉ። | 1. በጣም አልስማማም, 2. አልስማማም, 3. ገለልተኛ, 4. እስማማለሁ, 5. በጣም እስማማለሁ |  |
| **819** | የሃይማኖት አባቶች ለእናቶች እና ህጻናት ጤና እድገት ጠቃሚ የሆነ ድጋፍ ይሰጣሉ | 1. በጣም አልስማማም, 2. አልስማማም, 3. ገለልተኛ, 4. እስማማለሁ, 5. በጣም እስማማለሁ |  |
| **820** | በእርግዝና እና ልጅ በምወለድበት ጊዜ የሃይማኖት መሪዎች እንደረዱኝ ይሰማኛል | 1. በጣም አልስማማም, 2. አልስማማም, 3. ገለልተኛ, 4. እስማማለሁ, 5. በጣም እስማማለሁ |  |
| **821** | በእርግዝና እና በወሊድ ጊዜ የእናቶች እና የህፃናት ጤና አጠባበቅ እንድገኝ በማበረታታት የሃይማኖት መሪዎች እንደረዱኝ ይሰማኛል። | 1. በጣም አልስማማም, 2. አልስማማም, 3. ገለልተኛ, 4. እስማማለሁ, 5. በጣም እስማማለሁ |  |
| **822** | የአደጋ ምልክቶች በሚታዩበት ጊዜ ሁሉ የጤና እንክብካቤ ለመፈለግ ሲነሱ የሃይማኖት መሪዎች ድጋፍ እንደሚሰጡኝ ይሰማኛል። | 1. በጣም አልስማማም, 2. አልስማማም, 3. ገለልተኛ, 4. እስማማለሁ, 5. በጣም እስማማለሁ |  |
| **823** | የእናቶች እና ህፃናት ጤናን በተመለከተ ከሀይማኖት አባቶች ድጋፍ እንደተደረገልኝ ይሰማኛል። | 1. በጣም አልስማማም, 2. አልስማማም, 3. ገለልተኛ, 4. እስማማለሁ, 5. በጣም እስማማለሁ |  |
| **824** | የሃይማኖት መሪዎች ስለ እናቶች እና ህፃናት ጤና ጠንቅቀው ያውቃሉ ብዬ አምናለሁ። | 1. በጣም አልስማማም, 2. አልስማማም, 3. ገለልተኛ, 4. እስማማለሁ, 5. በጣም እስማማለሁ |  |
| **825** | የሃይማኖት መሪዎች የእናቶች እና የህፃናት ጤና መልዕክቶችን ሲያስተላልፉ ነፍሰ ጡር እናቶች እንደሚቀበሏቸው ተረድቻለሁ | 1. በጣም አልስማማም, 2. አልስማማም, 3. ገለልተኛ, 4. እስማማለሁ, 5. በጣም እስማማለሁ |  |
| **826** | የሃይማኖት መሪዎች ለእናቶች እና ህጻናት ጤና እድገት ታማኝ አስተዋፅዖ አበርክተዋል ብዬ አምናለሁ። | 1. በጣም አልስማማም, 2. አልስማማም, 3. ገለልተኛ, 4. እስማማለሁ, 5. በጣም እስማማለሁ |  |
| **827** | የሃይማኖት መሪዎች ስለ እናቶች እና ህፃናት ጤና ሲያስተምሩ እና ሲሰጡ ይታመናሉ ብዬ አምናለሁ። | 1. በጣም አልስማማም, 2. አልስማማም, 3. ገለልተኛ, 4. እስማማለሁ, 5. በጣም እስማማለሁ |  |
| **828** | የእናቶች ጤና አገልግሎት በአግባቡ ጥቅም ላይ መዋሉን እና አለመሆኑን የሀይማኖት መሪዎች እየተከታተሉት እንደሆነ እገነዘባለሁ። | 1. በጣም አልስማማም, 2. አልስማማም, 3. ገለልተኛ, 4. እስማማለሁ, 5. በጣም እስማማለሁ |  |
| **829** | የሃይማኖት መሪዎች ስለ መደበኛ የእናቶች እና ህጻናት ጤና አገልግሎት አጠቃቀምን የመከታተል ሃላፊነት እንደሚወስዱ ተረድቻለሁ | 1. በጣም አልስማማም, 2. አልስማማም, 3. ገለልተኛ, 4. እስማማለሁ, 5. በጣም እስማማለሁ |  |
| **830** | የሃይማኖት መሪዎች በታዘዘው መሠረት የብረት-ፎሌት መድኃኒቶችን የመቆጣጠር ኃላፊነት እንዳለባቸው እንደሚሰማቸው ተገንዝቤያለሁ | 1. በጣም አልስማማም, 2. አልስማማም, 3. ገለልተኛ, 4. እስማማለሁ, 5. በጣም እስማማለሁ |  |
| **831** | የሀይማኖት መሪዬ የእናቶች እና የህፃናት ጤና መልእክቶችን ሲያሰራጭ በመመልከቴ ተደንቄያለሁ እና ተጽኖአለሁ። | 1. በጣም አልስማማም, 2. አልስማማም, 3. ገለልተኛ, 4. እስማማለሁ, 5. በጣም እስማማለሁ |  |
| **832** | ከሀይማኖት መሪዎቼ ቤተሰብ ያየሁትን በመከተል የእናቶች እና የህፃናት ጤና አጠባበቅ አገልግሎቶችን በመደበኛነት እንደማገኝ እርግጠኛ ነኝ | 1. በጣም አልስማማም, 2. አልስማማም, 3. ገለልተኛ, 4. እስማማለሁ, 5. በጣም እስማማለሁ |  |
| **833** | የሃይማኖት መሪዎች በህመም ጊዜ እንክብካቤን በመፈለግ ረገድ አርአያ ሊሆኑ እንደሚችሉ ይሰማኛል። | 1. በጣም አልስማማም, 2. አልስማማም, 3. ገለልተኛ, 4. እስማማለሁ, 5. በጣም እስማማለሁ |  |
| **834** | የእናቶችና ህጻናት ጤና መልእክቶችን ሲያሰራጩ የሃይማኖት አባቶች ተስተውለዋል። | 1. በጣም አልስማማም, 2. አልስማማም, 3. ገለልተኛ, 4. እስማማለሁ, 5. በጣም እስማማለሁ |  |
| **835** | በቤተ ክርስቲያናችን/መስጊድ የሀይማኖት አባቶች የእናቶችንና የህፃናትን ጤና በማስተዋወቅ ረገድ ባብዛኛው ይስተዋላል | 1. በጣም አልስማማም, 2. አልስማማም, 3. ገለልተኛ, 4. እስማማለሁ, 5. በጣም እስማማለሁ |  |
| **836** | የሃይማኖት አባቶች ከእርግዝና፣ ምጥ እና ልጅ መውለድ ጋር የተያያዙ ጎጂ ልማዳዊ ድርጊቶችን እንደሚያበረታቱ በመግለጽ ይስተዋላል | 1. በጣም አልስማማም, 2. አልስማማም, 3. ገለልተኛ, 4. እስማማለሁ, 5. በጣም እስማማለሁ |  |

# Annex 2 Interview guide for qualitative study

**The role of religious leaders in promoting maternal and child health: a qualitative exploration of religious leaders’ perspective/view in rural setting of Hadiya Zone, Southern Ethiopia.**

**Interview guide**

1. Introductory questions
2. How many people are in your congregation?
3. How long have you had your position here?
4. Where were you previously appointed?
5. Do you have an idea of what the maternal health service utilizations coverage in your congregation is?
6. Do you receive questions about maternal health (ANC, SDS and PNC) from the members of your congregation?
7. What kinds of questions?
   - Interpretation of the bible and other text
   - Personal advice with regard to decision-making
   - Unhealthy beliefs and practices about maternal health service utilizations (ANC, SDS and PNC)
   - Unhealthy beliefs and practices about feeding new-borns
8. From whom and when?
9. How do you handle such?
10. Other questions?
11. Do you have an idea of the decision-making process regarding maternal health service utilizations in the families in your congregation? What factors are, in your opinion, decisive?
12. Do you, yourself, raise the topic of maternal health (ANC, SKD and PNC) for discussion?
13. During home visits?
14. During confirmation classes?
15. In sermons?
16. Otherwise
17. During congregation or on spiritual conference
18. Do you have contact with other religious leaders on the topic of maternal health or other topics?
19. From your own denomination?
20. From other denominations?
21. Regularly? Or only during spiritual conference?
22. Have you had contact with the government about maternal health (ANC, SDS and PNC) or other topics?
23. With the Kebele leaders or Head of Health office?
24. With the Health extension workers?
25. Regularly? Or only during spiritual conference?
26. What is your position on possibly obligatory maternal health (ANC, SDS and PNC)?
27. Is there anything else that you think is of importance and would therefore like to add?

## Annex 3 Interview guide for qualitative study Amharic Version

የቃለ መጠይቅ መመሪያ

1. የመግቢያ ጥያቄዎች

ሀ) በጉባኤያችሁ ውስጥ ስንት ሰዎች አሉ?

ለ) እዚህ ቦታዎ ለምን ያህል ጊዜ ኖረዋል?

ሐ) ከዚህ በፊት የተሾሙት የት ነበር?

መ) በጉባኤያችሁ ውስጥ የእናቶች ጤና አገልግሎት አጠቃቀም ሽፋን ምን እንደሆነ ሀሳብ አላችሁ?

1. ስለ እናቶች ጤና (ኤኤንሲ፣ኤስዲኤስ እና ፒኤንሲ) ጥያቄዎችን ከጉባኤዎ አባላት ይቀበላሉ?

ሀ) ምን አይነት ጥያቄዎች?

- የመጽሐፍ ቅዱስ እና የሌላ ጽሑፍ ትርጓሜ

- የውሳኔ አሰጣጥን በተመለከተ የግል ምክር

-ስለ እናቶች ጤና አገልግሎት አጠቃቀሞች (ኤኤንሲ፣ኤስዲኤስ እና ፒኤንሲ) ጤናማ ያልሆኑ እምነቶች እና ልምዶች

- አዲስ የተወለዱትን ስለመመገብ ጤናማ ያልሆኑ እምነቶች እና ልምዶች

ለ) ከማን እና መቼ?

ሐ) እንደዚህ ያሉትን እንዴት ነው የምትይዘው?

መ) ሌሎች ጥያቄዎች?

1. በጉባኤዎ ውስጥ ባሉ ቤተሰቦች ውስጥ የእናቶች ጤና አገልግሎት አጠቃቀምን በሚመለከት የውሳኔ አሰጣጥ ሂደት ሀሳብ አለዎት? በእርስዎ አስተያየት ምን ምክንያቶች ወሳኝ ናቸው?
2. እርስዎ እራስዎ የእናቶችን ጤና (ኤኤንሲ, ኤስኬዲ እና ፒኤንሲ) ለውይይት ያነሳሉ?

ሀ) በቤት ጉብኝት ወቅት?

ለ) በማረጋገጫ ትምህርት ጊዜ?

ሐ) በስብከት?

መ) ያለበለዚያ) በጉባኤ ወይም በመንፈሳዊ ጉባኤ ላይ

1. በእናቶች ጤና ወይም በሌሎች ርዕሰ ጉዳዮች ላይ ከሌሎች የሃይማኖት መሪዎች ጋር ግንኙነት አለህ?

ሀ) ከራስህ ቤተ እምነት?

ለ) ከሌሎች ቤተ እምነቶች?

ሐ) በመደበኛነት? ወይስ በመንፈሳዊ ጉባኤ ወቅት ብቻ?

1. ስለ እናቶች ጤና (ኤኤንሲ፣ኤስዲኤስ እና ፒኤንሲ) ወይም ሌሎች ርዕሶችን በተመለከተ ከመንግስት ጋር ግንኙነት ነበራችሁ?

ሀ) ከቀበሌ አመራሮች ወይም ከጤና ጽ/ቤት ኃላፊ ጋር?

ለ) ከጤና ኤክስቴንሽን ሠራተኞች ጋር?

ሐ) በመደበኛነት? ወይስ በመንፈሳዊ ጉባኤ ወቅት ብቻ?

1. በምናልባት የግዴታ የእናቶች ጤና (ANC፣ SDS እና PNC) ላይ የእርስዎ አቋም ምንድን ነው?
2. አስፈላጊ ነው ብለው የሚያስቡት ሌላ ነገር አለ እና ስለዚህ ማከል ይፈልጋሉ?

## Key message Amharic Version

**የሥልጠና ቁልፍ መልእክቶች**

**የእናቶች እና አዲስ የተወለደ ህመም እና ሞት ዋና መንስኤዎች እና የሚመከር እርምጃ**

**የእናቶች ሞት ዋና መንስኤዎች**

የደም መፍሰስ (የቅድመ ወሊድ እና የድህረ ወሊድ ደም መፍሰስ)፣ ኤክላምፕሲያ (በአደገኛ ከፍተኛ የደም ግፊት ምክንያት የሚከሰት)፣ የፐርፐራል ሴፕሲስ (ኢንፌክሽን)፣ ረጅም/የታገደ ምጥ እና ደህንነቱ ያልተጠበቀ ፅንስ ማስወረድ::

**አነስተኛ የእናቶች ህመም እና ሞት መንስኤዎች**

የደም ማነስ፣ ሳንባ ነቀርሳ፣ ቴታነስ፣ ኤችአይቪ/ኤድስ፣ የልብና የደም ቧንቧ በሽታ፣ በእርግዝና ወቅት ወባ፣ በእርግዝና ወቅት ከመጠን በላይ ማስታወክ እና የተመጣጠነ ምግብ እጥረት::

**አዲስ የተወለደ በሽታ እና ሞት ዋና መንስኤዎች**

ያለጊዜው መወለድ - ከ 37 ሳምንታት እርግዝና በፊት የተወለደ, ዝቅተኛ ክብደት - ከ 2.5 ኪሎ ግራም ክብደት በታች የተወለደ, ሃይፖሰርሚያ, ኢንፌክሽን እና የወሊድ አስፊክሲያ::

**የሚመከር እርምጃ**

የቅድመ ወሊድ እንክብካቤ ክትትል፣ በሰለጠነ የጤና ባለሙያዎች ማድረስ፣ የቅድመ ወሊድ እንክብካቤ በ24 ሰአት ውስጥ፣ የቤተሰብ ምጣኔ አገልግሎት እና አጠቃላይ የፅንስ ማስወረድ እንክብካቤ (ሲኤሲ)፣ የነፍሰ ጡር ሴቶች ኮንፈረንስ ማካሄድ እና የእናቶች መጠበቂያ ቤቶች (MWHs)::

**የትኩረት የቅድመ ወሊድ እንክብካቤ አካላት-**

ለችግሮች እና ውስብስቦች አስቀድሞ ማወቅ እና ማከም።

ለችግሮች እና ውስብስቦች አስቀድሞ ማወቅ እና ማከም።•ለብዙዎቹ በቅድመ ወሊድ እንክብካቤ ውስጥ አስፈላጊ የሆኑ ተግባራትን ከስር ያሉ ሁኔታዎችን አስቀድሞ መለየት በጣም አስፈላጊ ነው - ለምሳሌ የትውልድ ቂጥኝን መከላከል፣ የደም ማነስን መቆጣጠር እና የወባ ችግሮችን መከላከል።

ውስብስቦችን እና በሽታዎችን መከላከል (የብረት ፎሌት የደም ማነስን ለመከላከል፣ቴታነስ ቶክሳይድ፣በነፍሳት ላይ የሚደረግ ሕክምና የተጣራ አቅርቦት፣በጾታ ግንኙነት የሚተላለፉ ኢንፌክሽኖች እና የኤችአይቪ ምርመራ እና የምክር አገልግሎት)

**የወሊድ ዝግጁነት እና ውስብስብነት ዝግጁነት**

የመውሊድ ዝግጁነት እና ውስብስብነት ዝግጁነት ማለት የሰለጠነ የእናቶች እና አዲስ የተወለዱ አገልግሎቶችን ወቅታዊ ተደራሽነት ለማስተዋወቅ የታለሙ አጠቃላይ ፓኬጆች ማለት ነው።

ነፍሰ ጡር እናቶች እና ቤተሰቦቻቸው ለመውለድ ንቁ ዝግጅት እና ውሳኔን ያበረታታል.

የልደት ዝግጁነት፡- ለመደበኛ ልደት የማቀድ ሂደት ነው:: ውስብስብ ዝግጁነት በድንገተኛ ጊዜ የሚያስፈልጉትን ድርጊቶች አስቀድሞ በመጠባበቅ ላይ ነው።

የአደጋ ጊዜ እቅድ በአደጋ ጊዜ በፍጥነት መከናወን ያለባቸውን ድርጊቶች ሁሉ የመለየት እና የመስማማት ሂደት ነው, እና ዝርዝሮቹ ሁሉም ሰው እንዲረዱት እና አስፈላጊው ዝግጅት ይደረጋል::

**የልደት ዝግጁነት የሚከተሉትን ያጠቃልላል::**

ምርጫዎቿን ማክበር ስለ ደህንነት እና ንጽህና አቅርቦት ሁሉንም አስፈላጊ መረጃዎችን መስጠት አለቦት ነገርግን በመጨረሻ አንዲት ሴት መውለድ የምትፈልግበትን ቦታ እና በወሊድ ጊዜ ከማን ጋር መሆን እንደምትፈልግ መምረጥ አለብህ።

እሷን እና ቤተሰቧን በወሊድ ጊዜ እና በድህረ ወሊድ ጊዜ ውስጥ የድጋፍ ምንጮችን እንድትለይ መርዳት።

ከወሊድ ጋር የተያያዙ ተጨማሪ ወጪዎችን ማቀድ, ለእሷ እንክብካቤ እና አዲስ ለተወለደ ሕፃን እንክብካቤ ቁሳቁሶችን ማዘጋጀት.

**የልደት ዝግጁነት እና ውስብስብ ዝግጁነት አካላት**

ችሎታ ያላቸው የጤና ባለሙያዎች፣ ተቋም ወይም የትውልድ ቦታ፣ መጓጓዣ፣ ፈንዶች፣ ደጋፊ ሰው፣ ውሳኔ ሰጪ እና በወሊድ ላይ ያሉ አደገኛ ምልክቶች::

**በእርግዝና ወቅት አደገኛ ምልክቶች እና በአራስ ሕፃናት ውስጥ አደገኛ ምልክቶች**

የአደጋ ምልክት የእናቲቱን ወይም ያልተወለደውን ልጅ ወይም ሁለቱንም የሚያሰጋ ከባድ ነዉ:፡ በሽታ ነፍሰ ጡር ሴት የአደጋ ምልክት ካላት የአደጋ ምልክቶችን ማወቅ እና ወዲያውኑ እንክብካቤ ማግኘት አለባት።

**ቅድመ ወሊድ አደገኛ ምልክቶች፡** (ከባድ ራስ ምታት፣ የዓይን ብዥታ፣ ገዳይ የሆነ እንቅስቃሴ የለም፣ የደም ግፊት መጨመር፣ የፊት እብጠት፣ የእጆች/እግር እብጠት፣ መናድ፣ ከፍተኛ የሆነ የሴት ብልት ደም መፍሰስ፣ በታችኛው የሆድ ክፍል ውስጥ ከባድ ህመም እና ፈሳሽ መፍሰስ (ሜኮኒየም የቆሸሸ)።

**ምጥ/መውለድ አስጊ ምልክቶች**፡ (ከሴት ብልት ውስጥ ብዙ ደም መፍሰስ፣ መጥፎ ሽታ ያለው ፈሳሽ፣ ከፍተኛ ትኩሳት፣ የሕፃኑ እጅ ወይም እግር መጀመሪያ ይወጣል፣ ሕፃኑ ባልተለመደ ቦታ ላይ ነው፣ ምጥ ማራዘም (12 ሰአታት)፣ የተቀመጠ የእንግዴ ልጅ፣ የማህፀን ስብራት፣ ኮርድ መራባት፣ ገመድ፡፡

**ከወሊድ በኋላ አደገኛ ምልክቶች፡** (ከሴት ብልት ውስጥ ከመጠን በላይ ደም መፍሰስ፣ መጥፎ ሽታ ያለው ፈሳሽ፣ ከፍተኛ ትኩሳት፣ የተገለበጠ የጡት ጫፍ፣ ቴታነስ፣ የቆመ የእንግዴ ቦታ፣ ከባድ የሆድ ህመም፣ መናወጥ እና የተጨማለቀ ጡት/የጡት ማበጥ)፡፡

**ለአራስ ሕፃናት አደገኛ ምልክቶች፡** (በደካማ መመገብ ወይም ማጥባት አለመቻል፣ ተቅማጥ፣ የገመድ አካባቢ መቅላት፣ ቀይ አይን/የሚፈሰሱ አይኖች፣ የመተንፈስ ችግር፣ የቆዳ/ጃንዲስ ቢጫ ቀለም፣ ሃይፖሰርሚያ/ መንቀጥቀጥ፣ በቆዳ ላይ ጉድፍ/ የቆዳ ጉዳት፣ ህጻን አይጎዳም፡፡

ዶክተር ወይም ሌሎች የጤና ባለሙያዎችን ማማከር፡ የአደጋ ምልክቶች ካላቸው (በእርግዝና፣ ምጥ እና ወሊድ፣ ከወሊድ ከ42 ቀናት በኋላ እና አዲስ የተወለደ የአደጋ ምልክቶች) ካለባቸው።

# Key message Hadiyisa Version

**Losa’n horoori asso**

**Amoo’ikaa hareechi qaraamu cilluwwika lehiki horoori maska’uwwaa horaakam googuwwa.**

**Horoori ihaakkko ammaa’nne lehoo eboo luwwi:**

Xiigi dunaamchcha, xiigi gafechchi exximma,jabbinne amadamchcha, xuuchi dassimma lamifoollano amani bee’e gundismma.

**Hoffani ihaakkko ammaa’nne lehoo eboo luwwi;**

- Xiigi hoffe’nna, Suqqo’I jabbo, teetanosa, HIV/AIDS, wodda’nni jabbo, kachchisi jabbo, lamifoollano amannene erisa goxaansimmaa hurbaaxxi hoffe’nna.
- Horoori ihaakko hareecho qaraamukki ciilluwwi xissikkii lehikkii maska’uwwi;
- Saadi lamari saanti afoo’ni qoxxu bee’i ciilluwwi qaramchi, 2.5 kiilograminsi hofaani ihakko ciilluwwi qaraamchi, fooshee’imma hoogimmi, mulli jabbuwwimi lehina mashkka;I ihookko.

**Amoo’ikaa hareechi qaraamu cilluwwika lehoo xissoo hooriminaa baxximmi hassisoo baxxuwwi;**

- Lamifoollani la’aamukkisam qaqissimminne fayya’oommi eegechchi mine marimba, ayya’oommi eegechi bikkina losu manninne qarrimma, abaroosa qoodakka’ a qarimmi awaado awwaxximma, harreechchi ciillichchi qaramukki mati balli woronne fayya’oomi awwado siiximma, Lamifoolluwwi konfiransanne dummichchanne baxxamiimma, qarriimmina guddukki mine awwaaxximma.

**Lamifoollani ammane awwaximmi hassisoo horoori luwwi:**

Lamifoollani ammanenne waroo hawwo gaassaka’a la’immaa awado siixximma

***Awwaaduwoomimi:***

- xiigi hoffechcha ege’llimma, kachchisi jabbiinsi ege’llimma, sifi’lli jabboo li’inni hoffe’nni hawoo eg’llimma.
- Areeni fooleti yakkami xiigi hoffechcha horoo kiniina awwaxximma, tetanoosi qaraare awaaximma, kachchis jabbiinsi egeroo shara’a awwaxxiimma, edaanchinne higoo jabbuwwa HIV gaganne yooda’e la’immi awwado siixximma.
- Cillichchi qaramukkisam qaaqisakka’a anuuna iciisimma odim ciilichchi qramukkaannii lohi again afeebe’e xale’i anuuna iicisimma.
- Hareechchi qaraamu ciilluwwina Awwaadoobe’i hurbaata ittisimma urimmaa hawoodoo ama’nnatinsi gaga qeessimma.
- Lamifoollani ammane orraachchi mucuroomaa egee’llimma
- Qarrimmina hassisso luwwa gudiissimmaa kee’maa’lli hawwi waroota’niimi gudimma
- Gagi faya’ooma erisa egee’llimma

**Qarrimmina hassisso luwwa gudiissimmaa kee’maa’lli hawwi waroota’niimi gudimma**

Kuu guddimmi qaraamoo ciillichchi fayya’oominam ihukkoo ami fayya’omina horemi erisa awwaadookko.

Ku Qarrimmina hassisso luwwa gudiissimmaa amaadoo luwwi:

- Qariimmi bikkina losaakkoo mancho la’imma
- Qarakkam fayaa’oommi egechchi mine la’imma
- Qariimina marakkam amanina tiransporta gudisimma
- Qarammi amane maqire maroo manchoo hara’moo manna la’imma

**Lamifoollani ammanenne hareechchi qaramoo ciilluwwanne siidameena xanoo maree’wwa**

Ku siidameena xanoo maree’wwi amanaamii cillichchinammii foorina badiisoo bikkina ku maree’I siidamulas qaqisimminne fayya’oomi egee’llimmi mine marimmi hasiisookko.

- **Lamifoollani ammanenne he’eena xanoo jabbi mare’uwwi: (**kee’mmaa’li horoo’li damuumi , illi mo’immi hawwi, godaphi woronne yookki ciillichchiki mikimikaati bee’immi, xiigi gafechchi edimma, illi kolli dashshimma, angi te’imi lokki dashshimma, kullulaa’imma, qa’li ma’nninne xiigi firimma, kee’maa’lisa godabi xissimma.
- **Xuuchi amane te’mi qarimmi amane he’eena xanoo hawwi mare’uwwi: (**qa’li ma’nninne xiigi firimma, boboo daaadali luwwi firiimma, orachchi erisa iibbimma, ciiillichchiki angi gasaa qaramchcha, mati ballii lophphaakko xuuchi hee’imma, maqeeri qaraamoo’ni gattimmi, qa’li ma’nni xooqimma.
- **Qarimmi lasage hee’ena xanoo hawwi mare’e:** (qa’li ma’nninne xiigi firimma**,** boboo daaadali luwwi firiimma**,** orachchi erisa iibbimma**,** anuu’ni woroonne aagimma**,** maqeeri qaraamoo’ni gattimmi**,** kee’maa’lisa godabi xissimma**,** kulluulaa’immaa anuuni dashshimmaa)
- **Hareechchi qaraamu ciilluwwanne he’eena xanoo hawwi mare’e: (**anuuna iicimma hoogimma, godabi aadissimma**,** suri kasharimma**,** illi kasharimma**,** foosheechcha hoogimma**,** orachchi baari hagari ihimma**,** huxiisimma**,** orachchonne qoshi firimma**,** cillichchi larubeelas**,** orachchi shokiisimma**,** baqimma hogimma**,** qaqiiso qaqiiso foshee’imma**,** shumi te’mi ciri firimma hoogimma.
